# MiRNA Polymorphisms and Cancer Prognosis: A Systematic Review and Meta-Analysis

**DOI:** 10.3389/fonc.2018.00596

**Published:** 2018-12-13

**Authors:** Han-xi Ding, Zhi Lv, Yuan Yuan, Qian Xu

**Affiliations:** Tumor Etiology and Screening Department of Cancer Institute and General Surgery, The First Hospital of China Medical University, Key Laboratory of Cancer Etiology and Prevention (China Medical University), Liaoning Provincial Education Department, Shenyang, China

**Keywords:** miRNAs, single nucleotide polymorphisms, cancers, systematic review, meta-analysis

## Abstract

**Background:** Accumulating studies have focused on the relationship between miRNAs polymorphisms and cancer prognosis. However, the results are conflicting and unconvincing. This systematic review and meta-analysis was conducted to explore the relationship between miRNAs polymorphisms and cancer prognosis, aiming to seek for markers with cancer prognostic function.

**Methods:** Hazard ratio of overall survival, disease-free survival (DFS) and recurrence-free survival were calculated to evaluate the association between miRNAs polymorphisms and cancer prognosis by using Stata software 11.0.

**Results:** We systematically reviewed the association of 17 miRNAs SNPs with cancer prognosis including 24,721 samples. It was shown that 6 miRNAs SNPs (miR-608 rs4919510, miR-492 rs2289030, miR-378 rs1076064, miR-499 rs4919510, miR-149 rs2292832, miR-196a2 rs11614913) were associated with better cancer overall survival (OS) while let-7i rs10877887 was associated with poor OS; the homozygous and heterozygote genotype of miR-423 were related to poor cancer relapse-free survival (RFS) when compared with the wild genotype; miR-146 rs2910164 was linked to favorable cancer DFS while miR-196a2 rs11614913 was associated with poor DFS.

**Conclusions:** In summary, let-7i rs10877887, miR-608 rs4919510, miR-492 rs2289030, miR-378 rs1076064, miR-423 rs6505162, miR-499 rs4919510, miR-149 rs2292832, miR-146 rs2910164, and miR-196a2 rs11614913 might serve as potential biomarkers for cancer prognosis.

## Introduction

Despite emerging advances in the researches and understanding in tumor biology, cancer incidence remains rising and this global challenge further exacerbates by the increasing human life expectancy (1). It has been estimated that there will be ~19 million new cancer cases by 2025 (2). The high cancer-related morbidity and mortality contribute to urgent needs for novel biomarkers to help to evaluate the clinical outcome of cancer patients and enhance therapeutic effects to prolong their survival.

MicroRNAs (miRNAs), a class of non-coding RNAs with 19–25 nucleotides length, are small and regulatory RNAs binding to the 3′-UTR region of mRNA molecules. They have been regarded as key regulators in many diseases, particularly relevant in cancer (3, 4). It has been suggested that miRNAs may play oncogenic drivers or tumor suppressor roles in various cancer (3). The possible mechanism might be that the variation of miRNAs expression promote carcinogenesis, metastasis and many other characteristics of cancer by regulating the expression patterns of key genes involved in tumor growth and progression (5–7).

It has been widely recognized that the functional polymorphisms in miRNAs are the most common form of variation present in the human genome and could affect cancer susceptibility and prognosis (7–12). MiRNAs polymorphisms have been reported to influence the expression of mature miRNAs (13, 14). For instance, Chen et al. have reported that micRNA30c-1 polymorphisms could regulate the expression of mature miRNA 30c-1 and thus affect cancer prognosis (15, 16). A research by Yu et al. suggested the rs4938723 polymorphism could reduce micRNA-34b expression and increase the recurrence of early gastric cancer (17). Currently, accumulating studies have focused on the function of miRNAs polymorphisms and their relationship with cancer prognosis. However, the results were conflicting and unconvincing.

In the present study, a systematically review was conducted to investigate the association of miRNAs polymorphisms with overall survival (OS) time, recurrence-free survival (RFS) time as well as disease-free survival (DFS) time of cancer patients. Based on that, available data was collected to perform a meta-analysis to give a comprehensive assessment for the relationship between miRNAs polymorphisms and cancer prognosis. Data of this meta-analysis could expand our understanding of the role of miRNAs polymorphisms in human cancer prognosis, which may provide more credible evidences for future research in this field as well as find possible prognostic biomarkers and make an effort to assistant clinical decisions in the future.

## Methods

### Literature Mining

This study was carried out on the basis of Preferred Reporting Items for Systematic Reviews and Meta-analysis (PRISMA) (18).

Studies published in English language up to 20 October 2018 reporting on the association between the miRNAs polymorphism and cancer prognosis were identified by entering the following search terms into PubMed and Web of Science: “miRNA/miRNAs”; “polymorphisms/variants/variation/single nucleotide polymorphism/SNP”; and “cancer/carcinoma/tumor/neoplasm” and “prognosis/prognostic/outcome/survival.” Two independent investigators (Hanxi Ding and Qian Xu) performed the literature search. Eligible studies met the following criteria: (1) Concerning the association between miRNAs SNPs and cancer prognosis; (2) Involving prognostic indicators such as OS, DFS or RFS; (3) Including available HR and 95% CI. Articles were excluded based on the followings: (1) Duplicated studies or data; (2) Not relevant with miRNAs SNPs and cancer prognosis; (3) Lacking of available data or figures.

### Data Extraction

Two investigators (Hanxi Ding and Qian Xu) extracted the data independently and reached consensus regarding all the items. Study descriptions were obtained from each full text including author's name, year of publication, country of the origin, type of cancer, total number of the study population, the polymorphism site, the genotype, hazard ratio (HR), and corresponding 95% confidence interval (CI). In the absence of adequate information for estimation of HR or 95% CI, we have made all efforts to contact the authors to obtain sufficient information or extracted data from the Kaplan-Meier survival curves using a method suggested by Tierney et al. (19).

### Methodology Quality Assessment

Two reviewers (Hanxi Ding and Qian Xu) independently evaluated the quality of selected studies according to Newcastle-Ottawa Scale (20). Eight items categorized of three dimensions were assessed, including selection, comparability and exposure were assessed. The quality scores ranged between 0 and 9 stars (Supplementary Table 2).

### Statistical Analysis

The association of miRNAs polymorphisms with cancer OS, DFS and RFS was estimated through forest plots. Pooled HR and 95%CI were calculated by fixed-effects model or random-effects model. Pooled HR >1 suggested poor prognosis and was considered statistically significant if the 95%CI did not contain 1(21). In the absence of inter-study heterogeneity for Q-statistic with *p* > 0.05 and *I*^2^ < 50%, fixed-effect model was chosen to conserve statistical power, otherwise random-effect model was used (22, 23). Publication bias was assessed by Begger' test and if *p* > 0.05 was considered to be lack of publication bias (24). Sensitivity analysis was conducted by removing studies one by one. All analyses were performed using Stata software 11.0. All tests were two-sided and the results were considered to be statistically significant when the *p* < 0.05.

## Results

### Characteristics of the Eligible Studies

**Figure 1:**
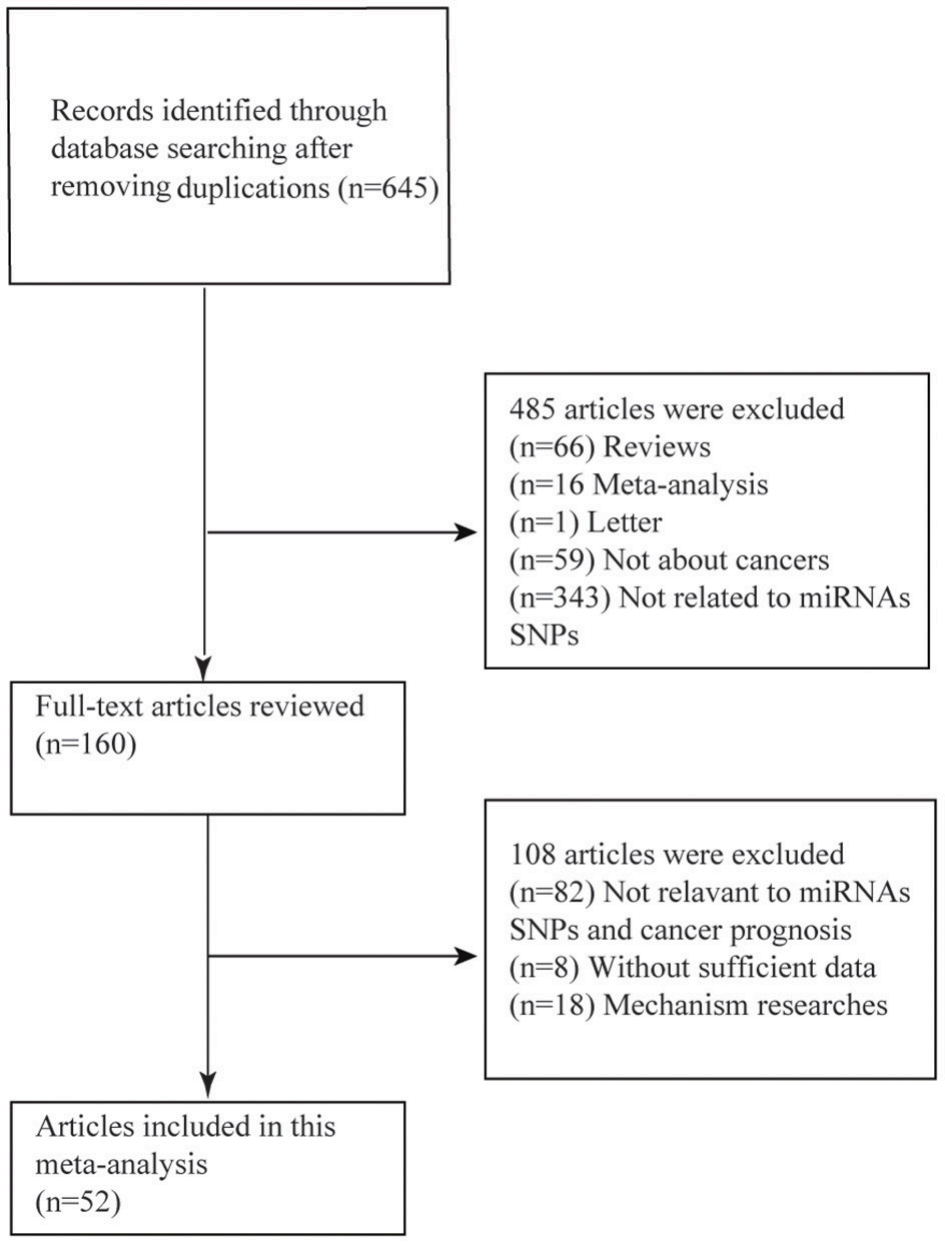
Flow diagram of the study selection process.

As was shown in the flow diagram, a total of 645 articles were enrolled in this systematic review. After multiple selections, 52 researches for 17 miRNAs SNPs including 24721 patients were involved in our meta-analysis (Figure 1) (4, 9–12, 25–68). Among the enrolled studies, 17 miRNAs were OS-related, 6 were RFS-related and 4 were DFS-related. The cancer type covered hepatocellular cancer (HCC), gastric cancer (GC), colorectal cancer (CRC), breast cancer, bladder cancer, non-small cell lung cancer (NSCLC), head and neck cancer, squamous cell carcinoma of the non-oropharynx (SCCOP). The original population came from Chinese, Korean, American, Canadian, Indian, and Polish. The characteristics of all the 52 studies were summarized in the Table 1 and the original data were shown in the Supplementary Tables 1, 4.

**Table 1:** Basic characteristics of the included articles in the meta-analysis.

**Author name**	**Publication year**	**Study population**	**miRNAs**	**Cancer**	**Samples size**	**Outcome**	**Citations**
Bri 'd M. Ryan	2012	American	mir-608(rs4919510)	CRC	245	OS	(63)
Brock C. Christensen	2010	American	mir-196a-2 (rs11614913)	HNSCC	323	OS	(27)
Chang Zheng	2017	Chinese	miR-219-1(rs107822, rs213210)	NSCLC	405	OS	(31)
Chengyuan Wang	2016	Chinese	mir-146a(rs2910164); mir-149(rs2292832); mir-196a2(rs11614913); mir-499(rs3746444)	SCCNOP	996	OS; DFS	(39)
Chung-Ji Liu	2013	Chinese	miR-196a2(rs11614913)	OSCC	315	OS	(9)
Dae Ho Ahn	2013	Korean	mir-146a(rs2910164); mir-149(rs2292832); mir-196a2(rs11614913); mir-499(rs3746444)	GC	160	OS	(45)
Fuzhen Qi	2014	Chinese	miR-106b-25(rs999885)	HCC	331	OS	(40)
Guopeng Yu	2016	Chinese	miR-492(rs2289030)	HCC	362	OS	(50)
Jeannette T. Bensen	2013	American	miR-16-1/15a(rs9535416); miR-206(rs6920648); miR-34b/c(rs4938723)	Breast cancer	1,946	OS	(30)
Jiali Xu	2013	Chinese	miR-27a(rs895819)	NSCLC	576	OS	(53)
Jiaze An	2014	Chinese	miR-378(rs1076064)	HCC	331	OS	(60)
Jing Jiang	2016	Chinese	miR-146a(rs2910164)	GC	838	OS	(12)
Jinliang Xing	2012	Chinese	miR-146a(rs2910164); miR-27a(rs895819); mir-423 (rs6505162); miR-492(rs2289030); miR-604(rs2368392); miR-605(rs2043556); miR-608 (rs4919510)	CRC	408	OS; RFS	(65)
Ji-Yong Ma	2015	Chinese	miR-27a(rs895819)	NSCLC	542	OS; RFS	(25)
Juan Li	2016	Chinese	miR-196a2(rs11614913)	HCC	109	OS	(49)
Kaipeng Xie	2013	Chinese	let-7 (rs10877887)	HCC	331	OS	(33)
Kaipeng Xie	2015	Chinese	miR-155(rs767649)	NSCLC	1,001	OS	(32)
Kyong-Ah Yoon	2012	Korean	mir-146a (rs2910164); miR-196a2(rs11614913); miR-219-1(rs213210); miR-26a-1(rs7372209); miR-27a(rs895819); miR-423 (rs6505162); miR-492(rs2289030)	NSCLC	388	RFS	(62)
Kyung Min Shin	2016	Korean	let-7a-1(rs10739971; rs1143770); let-7a-2(rs629367); let-7f-2(rs17276588)	NSCLC	761	OS; RFS	(58)
Lianghe Jiao	2014	Chinese	miR-125a(rs12976445); miR-218(rs11134527); miR-423(rs6505162); miR-608(s4919510)	Breast cancer	196	OS	(41)
Lin Jiang	2014	Chinese	miR-218(rs11134527)	ESCC	706	OS	(59)
Meenakshi Umar	2013	India	miR-146a(rs2910164);miR-196a2(rs11614913); miR-423(rs6505162); miR-499(rs3746444)	ESCC	153	OS	(56)
Meilin Wang	2012	Chinese	miR-146a(rs2910164)	Bladder cancer	74	RFS	(44)
Mi Jeong Hong	2013	Korean	mir-146a(rs2910164); miR-149(rs2292832); miR-196a2(rs11614913); miR-499(rs3746444)	NSCLC	363	OS; DFS	(55)
Moon JU Jang	2011	Korean	mir-146a(rs2910164); miR-149(rs2292832); miR-196a2(rs11614913); miR-499(rs3746444)	CRC	407	OS; RFS	(10)
Mulong Du	2014	Chinese	miR-196a(rs11614913)	RCC	311	OS	(48)
Myung Su Son	2013	Korean	miR-34 (rs4938723)	HCC	157	OS	(68)
Ning Zhan	2013	Chinese	miR-27a(rs895819)	Breast cancer	62	OS; RFS	(4)
Olusola O. Faluyi	2017	Canada	miR-124-1 (rs531564)	esophageal adenocarcinoma	369	OS	(37)
Pei-Wen Yang	2014	Chinese	mir-196a2(rs11614913); miR-26a-1(rs7372209); miR-30c-1(rs16827546); miR-423 (rs6505162); miR-608 (rs4919510)	ESCC	129	OS; RFS	(64)
Qian Xu	2014	Chinese	let-7a-2(rs629367)	GC	150	OS	(57)
Shizhi Wang	2013	Chinese	miR-196a2(rs11614913)	GC	940	OS	(11)
Shuangshuang Wu	2015	Chinese	miR-124-2(rs298206); miR-184(rs919968); miR-218-1(rs3775815)	NSCLC	1,001	OS	(36)
Soo Jung Lee	2014	Korean	miR-196a(rs11614913)	Early Breast cancer	452	OS; DFS	(42)
Won Hee Kim	2012	Korean	mir-146a(rs2910164); miR-149(rs2292832); miR-196a2(rs11614913); miR-499(rs3746444)	HCC	67	OS	(47)
Xia Lingzi	2016	Chinese	mir-146a(rs2910164); miR-149(rs2292832); miR-196a2(rs11614913); miR-423(rs6505162); miR-608(rs4919510)	NSCLC	584	OS	(46)
Xiao-Pin Ma	2016	Chinese	miR-608(rs4919510)	HCC	362	OS	(51)
Xiaoxiang Guan	2013	Chinese	mir-146a(rs2910164); miR-149(rs2292832); miR-196a2(rs11614913); miR-499(rs3746444)	SCCOP	281	OS; DFS	(38)
Xi-Dai Long	2016	Chinese	miR-1268a (rs28599926)	HCC	1,299	OS; RFS	(35)
Xingming Chen	2016	Chinese	mir-146a(rs2910164); miR-196(rs11614913); miR-423(rs6505162); miR-492(rs2289030)	SCCOP	1,008	DFS	(67)
Yang Zhao	2014	American	mir-182(rs129197463); miR-4302(rs11048315); miR-4422(rs17111728); miR-4741(rs7227168); miR-4742(rs7522956); miR-5197(rs2042253); miR-612(rs550894)	NSCLC	452	OS	(61)
Yanhua Wu	2017	Chinese	miR-218 (rs11134527); miR-219-1(rs213210); miR-34b/c(rs4938723); miR-938(rs2505901)	GC	735	OS	(66)
Yee Soo Cha	2013	Korean	miR-146a(rs2910164)	CRC	343	RFS	(43)
Yong-ping Mu	2012	Chinese	miR-30c (rs928508)	GC	92	OS	(54)
Z.Y. Sui	2016	Chinese	let-7 (rs10877887)	HCC	89	OS	(26)
Zhibin Hu	2008	Chinese	mir-146a(rs2910164); miR-149(rs2292832); miR-196a2(rs11614913); miR-499(rs3746444)	NSCLC	663	OS	(34)
Zhibin Hu	2011	Chinese	let-7a-2(rs629367); miR-1–2(rs9989532); miR-125b(rs2241490); miR-145(rs353291); miR-193b(rs30236); miR-29c(rs2724377); miR-30c-1(rs928508); miR-367(rs13136737); miR-378(rs1076064)	NSCLC	923	OS	(28)
Ying Li	2016	Chinese	let-7a-1(rs10739971)	GC	334	OS	(52)
Shizhi Wang	2014	Chinese	miR-107 (rs2296616); miR-107(rs78591545); miR-107(rs11185777)	GAC	940	OS	(29)
JAEJOON LIM	2018	Korean	miR-196a(rs1614913)	Brain cancer	179	OS	(69)
Na Cao	2018	Chinese	miR-379(rs61991156)	GC	217	OS	(70)
Marta Kotlarek	2018	Polish	miR-146(rs2910164)	PTC	315	OS	(71)

*HCC, hepatocellular carcinoma; NSCLC, non-small cell lung cancer; GC, gastric cancer; GAC, gastric adenocarcinoma; SCCOP, squamous cell carcinoma of the non-oropharynx; CRC, colorectal cancer; ESCC, esophageal squamous cell carcinoma; HNSCC, head and neck squamous cell carcinoma; OSCC, oral squamous cell carcinoma; PTC, papillary thyroid carcinoma*.

### Quantitative Data Synthesis of miRNAs SNPs

#### Rs10877887 in let-7i

Two relevant studies were included into this analysis to investigate the possible association between rs10877887 and cancer prognosis and the results suggested poor OS in CT+CC vs. TT model (HR = 1.32, 95%CI 1.09–1.60, *p* = 0.004). No heterogeneity was found in the meta-analysis process (*I*^2^ = 0.0%, *p* = 0.307, Table 2).

**Table 2:** Pooled HRs and 95%CIs from the meta-analysis for OS.

**Non-coding RNA**	**Model**	**No. of studies**	**No. of patients**	**HR (95%CI)**	***P*-value**	**Heterogeneity (%)**	**(I^**2**^, *P*-value)**
let-7i(rs10877887)	CT+CC vs. TT	2	420	**1.32 (1.09, 1.60)**	**0.004**	**0.0**	**0.307**
Let-7a-1(rs10739971)	GA vs. GG	2	1,095	1.01 (0.84, 1.21)	0.942	0.0	0.384
	AA vs. GG	2	1,095	0.87 (0.46, 1.64)	0.666	72.0	0.557
	GA+AA vs. GG	2	1,095	1.04 (0.86, 1.26)	0.655	0.0	0.557
let-7a-2(rs629367)	AC vs. AA	3	1,834	1.07 (0.76, 1.50)	0.694	70.90	0.016
	CC vs. AA	2	1,684	0.92 (0.64, 1.33)	0.666	0.0	0.747
	AC+CC vs. AA	2	1,684	0.93 (0.79, 1.09)	0.368	0.0	0.467
miR-218(rs11134527)	AG vs. AA	2	362	1.02 (0.82, 1.28)	0.856	0.0	0.356
	GG vs. AA	2	362	0.84 (0.61, 1.15)	0.284	46.9	0.170
	AG+GG vs. AA	2	249	0.89 (0.51, 1.54)	0.673	58.3	0.122
	GG vs. AG+AA	2	362	0.91 (0.54, 1.54)	0.733	60.5	0.112
mir-26a-1(rs7372290)	CT vs. CC	2	892	1.17 (0.96, 1.42)	0.117	0.0	0.975
	TT vs. CC	2	892	1.07 (0.77, 1.49)	0.677	0.0	0.874
miR-27a(rs895819)	CT vs. TT	3	1,526	1.28 (0.92, 1.79)	0.149	67.4	0.046
	CC vs. TT	3	1,526	1.25 (0.98, 1.59)	0.071	31.7	0.231
	CT+CC vs. TT	4	1,588	1.23 (0.86, 1.76)	0.254	51.1	0.105
	CC vs. CT+TT	2	950	1.09 (0.84, 1.41)	0.513	0.0	0.341
miR-34b/c(rs4938723)	TC+CC vs. TT	2	892	0.91 (0.75, 1.14)	0.338	0.0	0.845
	CC vs. TC+TT	2	2,103	**0.69 (0.48, 1.00)**	**0.047**	**0.0**	**0.656**
miR-423(rs6505162)	AC vs. CC	5	1,470	1.25 (0.91, 1.72)	0.176	56.1	0.059
	AA vs. CC	5	1,470	1.00 (0.73, 1.37)	0.990	0.0	0.406
	AC+AA vs. CC	2	604	1.20 (0.38, 3.76)	0.610	89.6	0.002
	AA vs. AC+CC	2	604	1.10 (0.53, 2.28)	0.789	0.0	0.485
miR-492(rs2289030)	GC vs. CC	2	768	**0.73 (0.56, 0.94)**	**0.014**	**0.0**	**0.963**
	GG vs. CC	2	768	0.88 (0.64, 1.20)	0.415	0.0	0.643
	GG vs. CG+CC	2	768	0.94 (0.69, 1.27)	0.676	0.0	0.532
	GC+GG vs. CC	2	768	**0.74 (0.58, 0.94)**	**0.014**	**0.0**	**0.848**
miR-499(rs3746444)	CT vs. TT	6	1,813	1.01 (0.85, 1.20)	0.896	0.0	0.554
	CC vs. TT	5	1,746	0.96 (0.61,1.51)	0.864	0.0	0.789
	CC vs. CT+TT	2	770	0.75 (0.33, 1.71)	0.498	0.0	0.484
	CT+CC vs. TT	2	567	1.14 (0.77, 1.68)	0.505	0.0	0.399
	TT vs. CT+CC	2	1,277	**0.65 (0.49, 0.85)**	**0.002**	**43.5**	**0.184**
miR-608(rs4919510)	CG vs. GG	4	1,858	**0.81 (0.70, 0.94)**	**0.004**	**24.1**	**0.267**
	CC vs. GG	4	1,858	0.84 (0.63, 1.13)	0.248	54.3	0.087
	CG vs. CC	2	441	1.14 (0.82,1.60)	0.434	0.0	0.542
	GG vs. CC	2	441	1.32 (0.77, 2.26)	0.317	0.0	0.794
	CC vs. CG+GG	3	966	**0.71 (0.55, 0.93)**	**0.013**	**0.0**	**0.978**
	CG+CC vs.GG	3	966	**0.74 (0.59, 0.93)**	**0.009**	**0.0**	**0.778**
miR-30c(rs928508)	AG+GG vs. AA	2	1,015	1.14 (0.43, 2.98)	0.793	88.0	0.004
miR-378(rs1076064)	AG vs. AA	2	1,254	**0.83 (0.69, 0.98)**	**0.032**	**0.0**	**0.378**
	GG vs. AA	2	1,254	0.72 (0.45, 1.16)	0.180	79.9	0.007
let-7a-1(rs10739971)	GA vs. GG	2	1,095	1.01 (0.73, 1.40)	0.934	0.0	0.567
	GA+AA vs. GG	2	1,095	1.04 (0.77, 1.42)	0.782	0.0	0.715
miR-146(rs2910164)	CG vs. CC	6	2,244	0.98 (0.86, 1.13)	0.820	0.0	0.805
	GG vs. CC	8	2,805	1.03 (0.86, 1.23)	0.758	23.8	0.240
	GC vs. GG	2	561	0.94 (0.68, 1.30)	0.728	0.0	0.377
	GC+GG vs. CC	5	1,357	0.84 (0.55, 1.30)	0.436	51.9	0.081
	GG vs. GC+CC	7	3,360	0.92 (0.70, 1.21)	0.549	57.4	0.029
miR-149(rs2292832)	CT vs. TT	5	1,581	**0.77 (0.66, 0.91)**	**0.002**	**0.0**	**0.516**
	CC vs. TT	6	2,244	**0.71 (0.57, 0.88)**	**0.002**	**42.3**	**0.123**
	CT+CC vs. TT	6	2,244	**0.76 (0.67, 0.86)**	**<0.001**	**9.5**	**0.355**
	CC vs. CT+TT	5	2,335	**0.62 (0.52, 0.74)**	**<0.001**	**0.0**	**0.472**
miR-196a2(rs11614913)	CT vs. TT	7	2,371	1.12 (0.89, 1.41)	0.335	40.6	0.121
	CC vs. TT	6	2,192	1.14 (0.75, 1.73)	0.548	67.8	0.008
	CT+CC vs. TT	6	1,439	1.28 (0.85, 1.93)	0.247	63.3	0.018
	CC vs. CT+TT	7	3,836	0.92 (0.67, 1.24)	0.572	79.4	< 0.001
	TC vs. CC	3	645	0.77 (0.60, 1.00)	0.048	0.0	0.568
	TT vs. CC	3	645	0.83 (0.58, 1.19)	0.304	0.0	0.848
	TC+TT vs. CC	2	644	**0.62 (0.46, 0.85)**	**0.003**	**49.8**	**0.158**
	TT vs. TC+CC	2	432	0.92 (0.50, 1.70)	0.787	76.5	0.029

*OS, overall survival; HR, hazard ratio; CI, confidence interval. Bolded values are expressed as values of statistical significance*.

#### Rs4919510 in miR-608

This polymorphism showed significant protective effects on cancer OS in CG vs. GG, CG+CC vs. GG and CC vs. CG+GG models (HR = 0.81, 95%CI 0.70–0.94, *p* = 0.004; HR = 0.74, 95%CI 0.59–0.93 *p* = 0.009; HR = 0.71, 95%CI 0.55–0.93, *p* = 0.013; respectively, Table 1). No heterogeneity was observed within the three models in the calculation processes (*I*^2^ = 24.1%, *p* = 0.267; *I*^2^ = 0.0%, *p* = 0.778; *I*^2^ = 0.0%, *p* = 0.978, respectively). Pooled data also suggested that heterozygote was associated with better RFS when compared with wild homozygote (HR = 0.73, 95%CI 0.60–0.88, *p* = 0.001). However, no significant relationship was indicated between rs4919510 and cancer DFS (Tables 2–4).

#### Rs2289030 in miR-492

For this polymorphism, only GC vs. CC and GC+GG vs. CC models were found to be associated with cancer OS (HR = 0.73, 95%CI 0.56–0.94, *p* = 0.014; HR = 0.74, 95%CI 0.58–0.94, *p* = 0.014, respectively, Table 2) but we failed to find any significant results about miR-429 SNP and RFS. And there was no meaningful heterogeneity among the molds in the meta-analysis.

#### Rs1076064 in miR-378

The heterozygote AG of rs1076064 in miR-378 was suggested to have a protective role in cancers OS when compared to the genotype AA (HR = 0.83, 95%CI 0.69–0.98, *p* = 0.032). No significant heterogeneity was found during calculation (*I*^2^ = 0.0%, *p* = 0.378, Table 2).

#### Rs6505162 in miR-423

The rs6505162 polymorphism was suggested to be associated with cancer RFS. The AC vs. CC and AC+AA vs. CC models indicated poor cancer prognosis (HR = 1.34, 95%CI 1.03–1.73, *p* = 0.026; HR = 1.37, 95%CI 1.01–1.86, *p* = 0.042, respectively) and we found no significant heterogeneity of these two SNPs (*I*^2^ = 24.1%, *p* = 0.268; *I*^2^ = 29.0%, *p* = 0.235, respectively Table 3). As for the relationship of rs6505162 with cancer OS and DFS, no related studies were involved in this meta-analysis.

**Table 3:** Pooled HRs and 95%CIs from the meta-analysis of RFS.

**Non-coding RNA**	**Model**	**No. of studies**	**No. of patients**	**HR (95%CI)**	***P*-value**	**Heterogeneity (%)**	**(I^**2**^, *P*-value)**
miR-27a(rs895819)	CT vs. TT	3	1,388	1.00 (0.83, 1.22)	0.963	23.7	0.270
	CC vs. TT	3	1,388	0.92 (0.72, 1.17)	0.483	0.0	0.827
	CT+CC vs.TT	4	1,400	0.99 (0.84, 1.18)	0.921	0.0	0.413
	CC vs. CT+TT	2	950	0.90 (0.71, 1.14)	0.373	0.0	0.933
miR-423(rs6505162)	AC vs. CC	3	925	**1.34 (1.03, 1.73)**	**0.026**	**24.1**	**0.268**
	AA vs. CC	3	925	0.89 (0.47, 1.70)	0.730	0.0	0.870
	AC+AA vs. CC	2	796	**1.37 (1.01, 1.86)**	**0.042**	**29.0**	**0.235**
miR-492(rs2289030)	GG vs. CC	2	796	1.10 (0.56, 2.15)	0.785	0.0	0.438
	GG vs. GC+CC	2	796	0.98 (0.65, 1.46)	0.910	0.0	0.894
miR-608(rs4919510)	CG vs. GG	2	912	**0.73 (0.60, 0.88)**	**0.001**	**0.0**	**0.401**
	CC vs. GG	2	912	0.82 (0.49, 1.35)	0.429	0.0	0.128
miR-146(rs2910164)	GC vs. CC	2	795	0.84 (0.57, 1.26)	0.400	60.4	0.080
	GG vs. CC	3	1,203	0.89 (0.66, 1.21)	0.462	37.1	0.189
	GG vs. GC+CC	3	889	0.89 (0.56, 1.40)	0.608	60.8	0.078
	GC+GG vs. CC	2	795	0.79 (0.35, 1.78)	0.570	83.0	0.015
	CC vs. GC+GG	2	751	1.37 (0.59, 3.20)	0.470	82.1	0.018
miR-196a2(rs11614913)	CT vs. TT	2	795	0.71 (0.49, 1.03)	0.074	0.0	0.715
	CC vs. TT	2	795	0.84 (0.54, 1.29)	0.423	0.0	0.369
	CT+CC vs. TT	2	795	0.76 (0.53, 1.08)	0.123	0.0	0.513

*RFS, relapse-free survival; HR, hazard ratio; CI, confidence interval. Bolded values are expressed as values of statistical significance*.

#### Rs3746444 in miR-499, rs2292832 in miR-149, rs2910164 in miR-146, and rs11614913 in miR-196a2

The TT vs. CT+CC model of rs3746444 polymorphism in miR-499 was associated with better OS (HR = 0.65, 95%CI 0.49–0.85, *p* = 0.002, Table 2). No association was suggested between miR-499 polymorphism and cancer RFS or DFS (Tables 3, 4). It was shown that miR-149 rs2292832 has significant protective effect on OS. Additionally, this effect was strengthened with the increasing variant C allele in each genetic model (CT vs. TT: HR = 0.77, 95%CI 0.66–0.91, *p* = 0.002; CC vs. TT: HR = 0.71, 95%CI 0.57–0.88, *p* = 0.002; CT+CC vs. TT: HR = 0.76, 95%CI 0.67–0.86, *p* < 0.001; CC vs. CT+TT: HR = 0.62, 95%CI 0.52–0.74, *p* < 0.001; respectively Table 2). No positive result was shown in it with cancer RFS or DFS. For the rs2910164 polymorphism in miR-146, only GG vs. GC+CC model suggested a protective role in DFS (HR = 0.70, 95%CI 0.51–0.97, *p* = 0.030; Table 4). No significant association was observed between rs2910164 and cancer OS or RFS in any genetic models. For miR-196a2 rs11614913 polymorphism, we also found that the TC+TT vs. CC model indicated better OS of cancer patients (HR = 0.62, 95%CI 0.46–0.85, *p* = 0.003; Table 2), and similar finding was indicated in cancer DFS (CC vs. CT+TT: HR = 1.71, 95%CI 1.02–2.84, *p* = 0.040; Table 4). However, it had no association with cancer RFS.

**Table 4:** Pooled HRs and 95%CIs from the meta-analysis of DFS.

**Non-coding RNA**	**Model**	**No. of studies**	**No. of patients**	**HR (95%CI)**	***P*-value**	**Heterogeneity (%)**	**(I^**2**^, *P*-value)**
miR-499(rs3746444)	TT vs. CT+CC	3	2,285	0.82 (0.37, 1.83)	0.635	91.2	< 0.001
miR-146(rs2910164)	GG vs. GC+CC	4	2,648	**0.70 (0.51, 0.97)**	**0.030**	**49.0**	**0.117**
miR-149(rs2292832)	CC vs. CT+TT	3	2,285	0.86 (0.62, 1.20)	0.385	63.5	0.065
miR-196a2(rs11614913)	CC vs. CT+TT	3	2,456	**1.71 (1.02, 2.84)**	**0.040**	**86.0**	**0.001**
	CT+TT vs. CC	2	644	0.51 (0.25, 1.05)	0.069	56.2	0.131

*DFS, disease-free survival; HR, hazard ratio; CI, confidence interval. Bolded values are expressed as values of statistical significance*.

### Other miRNAs SNPs and Cancers Prognosis

Other than the above-mentioned miRNAs SNPs, meta-analysis was also performed for the association of let-7a-2 (rs629367), miR-218 (rs11134527), miR-26a-1 (rs7372290), miR-27a (rs895819), miR-34b/c (rs4938723), miR-423 (rs6505162), miR-30c (rs928508), and let-7a-1 (rs10739971) with cancer OS and RFS. However, no statistical significance was demonstrated in them (Tables 2, 3).

### Stratified Data and Cancers Prognosis

Meta-analysis of all the included stratified data was conducted and the results indicated that CT+TT vs. CC model of miR-196a (rs11614913) was related to better OS as well as DFS in the never smoking subgroup (HR = 0.48, 95%CI 0.29–0.90, *p* = 0.005 for OS; HR = 0.54, 95%CI 0.35–0.84, *p* = 0.007 for DFS; Table 5).

**Table 5:** Pooled HRs and 95%CIs from the meta-analysis of stratified data.

**Variables**	**Non-coding RNA**	**Model**	**No. of studies**	**No. of patients**	**Outcome**	**HR (95%CI)**	**P-value**	**Heterogeneity (%)**	**(I^**2**^, *P*-value)**
**GENDER**									
Male	miR-196a(rs11614913)	CT+TT vs. CC	2	704	OS	1.75 (0.52, 2.65)	0.698	87.0	0.006
Female	miR-196a(rs11614913)	CT+TT vs. CC	2	239	OS	0.77 (0.17, 3.51)	0.730	85.3	0.009
**SMOKING**									
Ever	miR-196a(rs11614913)	CT+TT vs. CC	2	1,024	OS	0.90 (0.68, 1.19)	0.452	38.5	0.202
	miR-196a(rs11614913)	CT+TT vs. CC	2	335	DFS	0.88 (0.52, 1.44)	0.584	80.6	0.023
Never	miR-196a(rs11614913)	CT+TT vs. CC	2	1,024	OS	**0.48 (0.29, 0.90)**	**0.005**	**0.0**	**0.819**
	miR-196a(rs11614913)	CT+TT vs. CC	2	335	DFS	**0.54 (0.35, 0.84)**	**0.007**	**0.0**	**0.667**

*OS, overall survival; DFS, disease-free survival; HR, hazard ratio; CI, confidence interval. Bolded values are expressed as values of statistical significance*.

### Heterogeneity

In the overall comparisons, no inter-study heterogeneity was associated with cancer prognosis (OS, RFS, and DFS, Tables 2–4). With respect to the heterogeneity in other overall comparisons, sensitivity analysis was performed subsequently.

### Publication Bias

Begg's test was used to evaluate the potential publication bias of included studies. No statistically significant publication bias was indicated in any of the models for all involved miRNAs SNPs (Supplementary Table 3).

## Discussion

In this study, we systematically reviewed 645 relevant published articles. Meanwhile, 52 researches for 17 SNPs including 24,721 patients were involved in this meta-analysis. The results indicated that except that the variant genotype of let-7i rs10877887 (OS), miR-423 rs6505162 (RFS), miR-196 rs11614913 (DFS) showed worse outcomes for cancer survival, others (including miR-608 rs4919510, miR-492 rs2289030, miR-378 rs1076064, miR-499 rs3746444, miR-149 rs29101644, miR-196 rs11614913, and miR-146 rs2292823) all played protective role in cancer survival. This study would provide theoretical and research clues for future exploration.

### let-7i rs10877887: Association With Poor Cancer Prognosis

Let-7i rs10877887 is located in the promoter region of miRNA let-7, which was a well-known tumor suppressor of multiple cancers and incorporates a CpG island, TF biding sites, and DNase peak (72–74). Du et al. suggested miRNA let-7 acted as a tumor suppressor in RCC via down-regulating C-myc and C-myc's target gene (73). It has also been reported that low let-7i expression was independently associated with CRC distant metastasis and significantly linked to poor survival in CRC patients (75). Similar result was found in another research on lung cancer by Huang et al. (76).

On account of the location and incorporation of rs10877887, the T > C variant may influence the binding of transcription factors and let-7 expression (33, 77). Liu's research suggested that patients harboring rs10877887 CC genotype had a lower let-7i expression in CSCC tissues (78). In addition, the T > C variation of rs10877887 may have strong affinity with Myeloid zinc finger 1 (MAF1), a transcription factor which can promote the activity of Axl promoter, resulting in tumor cell migration, invasion and metastasis (33).

Our results suggested that the CT/CC genotype of rs10877887 plays a worse role in cancer prognosis when compared with wild homozygote. Probably it was because that deregulated let-7i expression associated with T > C mutation in cancer tissues. We could speculate that T > C variants may serve as biomarker of poor cancer prognosis as well as assistant clinical-decisions. Much more researches were needed to confirm this result as well as detect the potential mechanisms and functions in the future.

### miR-608 rs4919510: Association With Better Cancer Prognosis

MiRNA 608 harbors one SNP, rs4919510 C > G, located in +22bp of its mature 25 bp sequence. It is at the joint of the stem with the canonical hairpin loop, which has been reported to play a key role in cancer progression and be associated with prognosis of several cancer recently (41, 46, 51, 63–65, 79–81). Zheng et al. have found that rs4919510 SNP might influence the expression of miR-608 target genes including cell growth-related genes, tumor invasion and metastasis-related genes and cancer death-related genes (82).

Among the studies included in our meta-analysis, CG vs. GG, CC vs. CG+GG, and CG+CC vs. GG models of miR-608 were associated with overall cancer prognosis, all having protective effects. The possible mechanism might be that the CG, CC, and CG+CC genotypes of rs4919510 could influence the expression level of miR-608 target genes by regulating the miR-608 expression and exert positive roles in cancer prognosis. The reason why no statistical significant results were found to be related to cancer RFS may result from the relatively small number of articles researching about the miR-608 rs4919510 polymorphisms and RFS. In conclusion, miR-608 rs4919510 is associated with cancer OS and could be used for specific prediction of cancer prognosis and may direct clinical-decisions in the future with abundant mechanisms as well as functions evidences.

### miR-492 rs2289030: Association With Better Cancer Prognosis

As we know, mounting studies have shown that miR-492 plays an important role in cell tumorigenicity of multiple cancers (83–85). von Frowein et al found that up-regulation of miR-492 enhanced proliferation, anchorage-independent growth, migration, and invasion of hepatoblastoma by regulating CD44, which is a receptor for hyaluronan, the major component of the ECM and as well as a co-receptor for multiple cytokine signals and growth factors (83). Shen et al have revealed that ectopic expression of miR-492 contributed to deregulation of SOX7, leading to up-regulation of cyclin D1, c-Myc, and Rb phosphorylation, which could promote cell proliferation and cell cycle of breast cancer (84).

Single nucleotide polymorphisms of miRNAs have been identified to affect miRNA processing and alter miRNA expression (50). Some researchers have shown that miR-492 rs2289030 G > C was associated with various cancers (50, 62, 67, 86). Lee et al found CRC patients carried CG and GG genotype demonstrated worse RFS when compared with CC genotype. However, all enrolled studies in our meta-analysis showed that the GC and GC/GG genotypes played a positive role in cancers OS (87). Although our findings suggested that the GC and GG variation of miR-492 may improve cancers prognosis, more rounded investigations are needed to elucidate the association between miR-492 rs2289030 and cancer prognosis as well as the special mechanisms for the limited studies and inconsistent results.

### miR-378 rs1076064: Association With Better Cancer Prognosis

It has been reported that the A to G base change of SNP rs1076064 at 222 bp upstream from miR-378 may alter the expression of miR-378 (60). Accumulating researches have indicated that miR-378 was down-regulated in CRC, GC, and oral cancer (88–90). MiR-378 may exert tumor suppressor roles by deregulating the expression of CDK6 and VEGF in GC. Wang et al suggested that miR-378 inhibited cell proliferation by targeting CDC40 (88, 90). However, other studies have demonstrated that the up-regulation of miR-378 was related to several kinds of cancer including breast cancer, RCC and AML (91–93). Lee et al found that miR-378 could promote cell survival, tumor growth and angiogenesis by targeting at SuFu and Fus-1 (94).

An's research revealed that the variant genotype of rs1076064 acted as a transcription regulator of miR-378 and the G allele of rs1076064 may exert higher promoter activity in many cancer cell lines and was associated with a better prognosis in HCC (60). The meta-analysis results of the two involved articles also showed that AG vs. AA model was associated with better cancer OS, which was consistent with previous study. However, more studies were needed to confirm this result and translate the mechanisms in the future.

### miR-423 rs6505162: Association With Poor Cancer Prognosis

MiR-423, relevant to NSCLC, CRC, and breast cancer, is located in frequently amplified region of chromosome 17q11.2 (95–97). Zhao et al have revealed that miR-423 played a potentially oncogenic role in breast carcinogenesis by promoting cell proliferation of breast cancer cell lies (97). Similarly, Sun et al suggested the overexpression of miR-423 could decrease BRMS1 level obviously and promote cell invasion of HCC (98).

The study of Xing et al demonstrated that the variant-containing genotype AC/AA of rs6505162 in miR-423 was significantly associated with poor OS and RFS of CRC patients (65). However, another research by Lin et al suggested the A allele of miR-423 rs6505162 was associated with decreased RCC recurrence and better prognosis by weakening the capacity to target KLF2 mRNA, leading to inhibition of angiogenic pathways and cancer recurrence (99). The meta-analysis results of rs6505162 in our study showed that AC and AC/AA genotypes were associated with poor cancer RFS, which was consistent with Xing's research. However, only two related studies were enrolled in this meta-analysis, further relevant investigations are needed to obtain more reliable results.

### miR-146 rs2910164, miR-196a2 rs11614193, miR-149 rs2292832, and miR-499 rs3746444: Association With Cancer Prognosis

It has been well acknowledged that miRNAs SNPs could influence cancer prognosis by affecting miRNAs maturation or ability to combine with mRNAs target gene (100, 101).

Researches showed the variant G allele of miR-146 rs2910164 G > C, located in the 3′ miRNA passenger strand, may enhance the expression of mature miR-146 and the miR-146 overexpression has been found to suppress breast cancer metastasis (102, 103). This meta-analysis results of miR-146 rs2910164 showed that only the GG vs. GC+CC model was associated with better cancer DFS, which was consistent with Xia's meta-analysis (14). It could be inferred that the variant G allele might lead to increased expression of miR-146a and thus better prognosis.

MiR-196a has been regarded as an oncogene in cancers pathogenesis such as proliferation, migration and invasion (55). MiR-196a2 rs11619413 T > C, located in the 3′ messenger strand of miR-196a2, could influence the combination with target gene and the CC genotype was related to increased expression of miR-196a2 (34, 49, 104). The Meta-analysis showed that TC+TT vs. CC model was associated with better cancer OS while CC vs. CT+TT model was associated with poor cancer DFS, suggesting that CC allele might have risk effects on cancer prognosis. These results were contrary to Wang et al but consistent with Hu's research about lung cancer (11, 34). The differences may result from the different types of cancers or the baseline characteristics and more large number samples researches are needed to clarify the relationship between miR-196a2 rs11614913 and cancer prognosis.

MiR-149 is reported to be a pro-apoptotic miRNA, which can inhibit the expression of Akt1 and E2F1 and thus induce cancer cell lines apoptosis (99, 105). A function study of Xia et al suggested that C allele could increase the expression of miR-149, therefore leading to better prognosis in NSCLC (46). In this meta-analysis, we found four models of miR-149 rs2292832 including CT vs. TT, CC vs. TT CT+CC vs. TT, and CC vs. CT+TT were associated with better cancer OS, which was consistent with previous meta-analysis (106). We could infer that variant C allele may enhance miR-149 expression which was associated with better cancer prognosis and may play role as specific biomarker of cancer prognosis.

MiR-499 rs3736444 is located in the stem region opposite to the mature miR-499 sequence genetic and the T to C variation may influence miR-499 expression (39, 107). Ma et al have reported that the C allele could suppress miR-499 expression, resulting in decreased expression of Ets1, which therefore promotes HCC development and cause poor cancer outcome (108). The present study showed that TT vs. CT+CC model was associated with better cancer OS which was consistent with the results of Qiu's study that rs3746444 C to T variant could contribute to unfavorable cancer prognosis by regulating the expression of cancer-related genes (109). Therefore, we could speculate that variants containing C allele may play the role of miR-499 expression inhibitor and results in poor cancer outcome. Although there were statistically significant relationships, it should be noticed that only two relevant studies were involved, thus more convincing results need more studies on this field.

### Other miRNAs: Association With Cancer Prognosis

In addition to the above-mentioned 9 miRNAs polymorphisms, other miRNAs SNPs involved were also reviewed and processed in this meta-analysis. No significant association with cancer prognosis was discovered in our meta-analysis, but some of them were reported to influence the development and invasion of cancers and the corresponding mechanisms have been elaborated. For example, the transition from A to G of rs1113427 may alter the local secondary structure of miR-218 and then influence the expression of miR-218 which could inhibit the invasion and metastasis of GC by targeting the Robol receptor (66, 110, 111). Zhang et al. found rs895819 could affect the secondary structure of pre-miR-27a that subsequently influence the processing and maturation of miR-27a (4). Additionally, only two researches were available in the meta-analysis for multiple SNPs, so more relevant investigations should be included for updating the findings on the association between these polymorphisms and cancer prognosis in the future.

### Stratified Meta-Analysis: miRNAs SNPs and Cancer Prognosis

Further, we have collected all the available stratified data of included researches. It is suggested only the CT+TT genotype of miR-196a rs11614913 was associated to better cancer prognosis in the never smoking subgroup. We may speculate that CT +TT genotype of miR-196a rs11614913 maybe a potential biomarker for cancer prognosis in the specific subgroup. More evidences were needed to strengthen this conclusion in the future.

### Advantages and Limitations

Our study had some advantages. First, we collected all the published articles related to miRNAs SNPs and cancer prognosis, making the systematic review and meta-analysis comprehensive and complete. Second, this is the first meta-analysis concerning the association between miRNAs SNPs and cancer prognosis. Moreover, this study is reliable and stable due to the large number of enrolled patients (24,721) and the strict inclusion criteria.

Undoubtedly, some limitations should be acknowledged in our study. First, only English literature was searched, which may results in publication bias. Second, although the overall sample size were up to 24,721 patients enrolled, the number of some miRNAs SNP relevant studies was too small thus we could only preliminarily analyze the relationships between miRNAs polymorphisms and cancer prognosis with the currently published literatures. What's more, many of the included articles were without clear statistical power thus we could have no idea whether there were type I error and type II error in the original data. Finally, several original texts have no available data and we extracted data from figures which might lead to some bias.

## Conclusion and Future Directions

We systematically reviewed the researches about the association between miRNAs SNPs and cancer prognosis. Meanwhile, available data was used to perform a meta-analysis for SNPs with OS, RFS, and DFS of solid cancer. The relationships between miRNAs polymorphisms and cancer prognosis could be categorized into four types: (1) a better association, SNPs were linked to a better cancer prognosis such as miR-608 rs4919510 and miR-378 rs1076064; (2) a worse association, SNPs were associated with poor cancer prognosis including miR-423 rs6505162 and miR-196a2 rs1161913; (3) no association, no significant association was found between miRNAs SNPs and cancer survival; (4) failed to be quantitatively synthesized due to limited studies. Our study suggested quite a few miRNAs SNPs were associated with cancer prognosis, which would provide clues for further exploration on prognostic biomarkers.

Following aspects should be focused on in the future investigations. First, more miRNAs SNPs that may be associated with cancer prognosis (OS, RFS, and DFS) should be screened out to provide more alternative prognostic biomarkers. Second, more functional studies are needed to explore the mechanisms of SNPs within miRNAs in caner prognosis. Thirdly, in addition to the association between SNPs and cancer prognosis, other aspects such as chemotherapeutic susceptibility and drug tolerance are also needed to be illuminated. Finally, whether the exosome could carry some specific miRNA SNPs to or leave neoplastic foci may be explored to find some new clinical targets as well as therapeutic targets. Therefore, the clinical application of miRNAs polymorphisms has extremely extensive prospects and requiring further exploration.

## Author Contributions

HD and QX extracted and evaluated the data. HD calculated and wrote this paper. ZL revised this paper. QX and YY conceived the study. All authors read and approved the final manuscript.

### Conflict of Interest Statement

The authors declare that the research was conducted in the absence of any commercial or financial relationships that could be construed as a potential conflict of interest.

## Abbreviations

OS: overall survival
DFS: disease-free survival
RFS: relapse-free survival
HCC: hepatocellular cancer
GC: gastric cancer
CRC: colorectal cancer
NSCLC: non-small cell lung cancer
SCCOP: squamous cell carcinoma of the non-oropharynx
PTC: papillary thyroid carcinoma.
