# Supplementary material for: MiRNA Polymorphisms and Cancer Prognosis: A Systematic Review and Meta-Analysis
Source: Front Oncol. 2018 Dec 13;8:596. doi: 10.3389/fonc.2018.00596 (PMC6300499; doi:10.3389/fonc.2018.00596)
Supplement: Supplementary file 1 [file Table_1.DOCX]

# Supplementary Materials

# MiRNA Polymorphisms and Cancer Prognosis: a Systematic Review and Meta-analysis

Han-xi Ding^1^, Zhi Lv^1^, Yuan Yuan^1^, Qian Xu^1^*

^1^Tumor Etiology and Screening Department of Cancer Institute and General Surgery, the First Hospital of China Medical University, and Key Laboratory of Cancer Etiology and Prevention (China Medical University), Liaoning Provincial Education Department, Shenyang 110001, China

***Corresponding author:** Dr. Qian Xu, Tumor Etiology and Screening Department of Cancer Institute and General Surgery, the First Hospital of China Medical University, No.155 NanjingBei Street, Heping District, Shenyang, Liaoning Province, P.R. China 110001

Telephone：+86-024-83282153; fax: +86-024-83282383. Email：[qxu@cmu.edu.cn](mailto:qxu@cmu.edu.cn)

<Contents>

Supplementary Table 1. Original data of the included articles in this meta-analysis

Supplementary Table 2. Detailed information for NEWCASTLE - OTTAWA QUALITY ASSESSMENT SCALE

Supplementary Table 3. The results of Begg's and Egger's test for the publication bias

Supplementary Table 4. Stratified data of the included articles in this meta-analysis

| **Supplementary Table 1. Original data of the included articles in this meta-analysis** | | | | | | | | | | | |
| --- | --- | --- | --- | --- | --- | --- | --- | --- | --- | --- | --- |
| **miRNAs** | **cancer** | **model** | **Author Name** | **Publication Year** | **Study population** | **samples size** | **outcome** | **HR** | **95% UPPER** | **95% LOWER** | **Citation** |
| let-7i (rs10877887) | HCC | TT vs. CT+CC | Z.Y. Sui | 2016 | Chinese | 89 | OS | 0.68 | 0.94 | 0.52 | [[1](#_ENREF_1)] |
| let-7i (rs10877887) | HCC | CT vs. TT | Kaipeng Xie | 2013 | Chinese | 331 | OS | 1.15 | 1.51 | 0.88 | [[2](#_ENREF_2)] |
| let-7i (rs10877887) | HCC | CC vs. TT | Kaipeng Xie | 2013 | Chinese | 331 | OS | 1.58 | 2.37 | 1.05 | [[2](#_ENREF_2)] |
| let-7i (rs10877887) | HCC | CT+CC vs. TT (Dominant model) | Kaipeng Xie | 2013 | Chinese | 331 | OS | 1.23 | 1.58 | 0.96 | [[2](#_ENREF_2)] |
| let-7(rs13293512) | HCC | CT vs. TT | Kaipeng Xie | 2013 | Chinese | 331 | OS | 1.05 | 1.40 | 0.96 | [[2](#_ENREF_2)] |
| let-7(rs13293512) | HCC | CC vs. TT | Kaipeng Xie | 2013 | Chinese | 331 | OS | 0.73 | 1.04 | 0.96 | [[2](#_ENREF_2)] |
| let-7(rs13293512) | HCC | CT+CC vs. TT (Dominant model) | Kaipeng Xie | 2013 | Chinese | 331 | OS | 0.93 | 1.22 | 0.96 | [[2](#_ENREF_2)] |
| let-7a-1(rs10739971) | NSCLC | GA vs. GG | Kyung Min Shin | 2016 | Korean | 761 | OS | 0.99 | 1.39 | 0.96 | [[3](#_ENREF_3)] |
| let-7a-1(rs10739971) | NSCLC | AA vs. GG | Kyung Min Shin | 2016 | Korean | 761 | OS | 1.14 | 1.72 | 0.96 | [[3](#_ENREF_3)] |
| let-7a-1(rs10739971) | NSCLC | GA+AA vs. GG | Kyung Min Shin | 2016 | Korean | 761 | OS | 1.03 | 1.42 | 0.96 | [[3](#_ENREF_3)] |
| let-7a-1(rs10739971) | NSCLC | GG vs. GA vs. AA | Kyung Min Shin | 2016 | Korean | 761 | OS | 1.06 | 1.31 | 0.96 | [[3](#_ENREF_3)] |
| let-7a-1(rs10739971) | NSCLC | GA vs. GG | Kyung Min Shin | 2016 | Korean | 761 | RFS | 0.87 | 1.12 | 0.96 | [[3](#_ENREF_3)] |
| let-7a-1(rs10739971) | NSCLC | AA vs. GG | Kyung Min Shin | 2016 | Korean | 761 | RFS | 0.83 | 1.16 | 0.96 | [[3](#_ENREF_3)] |
| let-7a-1(rs10739971) | NSCLC | GA+AA vs. GG | Kyung Min Shin | 2016 | Korean | 761 | RFS | 0.86 | 1.10 | 0.96 | [[3](#_ENREF_3)] |
| let-7a-1(rs10739971) | NSCLC | GG vs. GA vs. AA | Kyung Min Shin | 2016 | Korean | 761 | RFS | 0.91 | 1.07 | 0.96 | [[3](#_ENREF_3)] |
| let-7a-2(rs1143770) | NSCLC | CT vs. CC | Kyung Min Shin | 2016 | Korean | 761 | OS | 0.74 | 1.01 | 0.96 | [[3](#_ENREF_3)] |
| let-7a-2(rs1143770) | NSCLC | TT vs. CC | Kyung Min Shin | 2016 | Korean | 761 | OS | 0.52 | 0.79 | 0.96 | [[3](#_ENREF_3)] |
| let-7a-2(rs1143770) | NSCLC | CT+TT vs. CC | Kyung Min Shin | 2016 | Korean | 761 | OS | 0.67 | 0.91 | 0.96 | [[3](#_ENREF_3)] |
| let-7a-2(rs1143770) | NSCLC | CC vs. CT vs. TT | Kyung Min Shin | 2016 | Korean | 761 | OS | 0.72 | 0.89 | 0.96 | [[3](#_ENREF_3)] |
| let-7a-2(rs1143770) | NSCLC | CT vs. CC | Kyung Min Shin | 2016 | Korean | 761 | RFS | 0.76 | 0.99 | 0.96 | [[3](#_ENREF_3)] |
| let-7a-2(rs1143770) | NSCLC | TT vs. CC | Kyung Min Shin | 2016 | Korean | 761 | RFS | 0.70 | 0.96 | 0.96 | [[3](#_ENREF_3)] |
| let-7a-2(rs1143770) | NSCLC | CT+TT vs. CC | Kyung Min Shin | 2016 | Korean | 761 | RFS | 0.74 | 0.95 | 0.96 | [[3](#_ENREF_3)] |
| let-7a-2(rs1143770) | NSCLC | CC vs. CT vs. TT | Kyung Min Shin | 2016 | Korean | 761 | RFS | 0.83 | 0.98 | 0.96 | [[3](#_ENREF_3)] |
| let-71-1(rs10739971) | GC | GA vs. GG | Ying Li | 2016 | Chinese | 334 | OS | 1.48 | 5.65 | 0.96 | [[4](#_ENREF_4)] |
| let-71-1(rs10739971) | GC | GA+AA vs. GG | Ying Li | 2016 | Chinese | 334 | OS | 1.32 | 4.80 | 0.96 | [[4](#_ENREF_4)] |
| let-71-1(rs10739971) | GC | AA vs. GG+GA | Ying Li | 2016 | Chinese | 334 | OS | 0.66 | 2.88 | 0.96 | [[4](#_ENREF_4)] |
| let-7a-2(rs629367) | NSCLC | AC vs. AA | Zhibin Hu (screening) | 2011 | Chinese | 568 | OS | 0.86 | 1.10 | 0.96 | [[5](#_ENREF_5)] |
| let-7a-2(rs629367) | NSCLC | CC vs. AA | Zhibin Hu (screening) | 2011 | Chinese | 568 | OS | 0.80 | 1.42 | 0.96 | [[5](#_ENREF_5)] |
| let-7a-2(rs629367) | NSCLC | AC+CC vs. AA | Zhibin Hu (screening) | 2011 | Chinese | 568 | OS | 0.85 | 1.08 | 0.96 | [[5](#_ENREF_5)] |
| let-7a-2(rs629367) | NSCLC | AC vs. AA | Zhibin Hu (validation) | 2011 | Chinese | 355 | OS | 0.88 | 1.24 | 0.96 | [[5](#_ENREF_5)] |
| let-7a-2(rs629367) | NSCLC | CC vs. AA | Zhibin Hu (validation) | 2011 | Chinese | 355 | OS | 1.12 | 2.15 | 0.96 | [[5](#_ENREF_5)] |
| let-7a-2(rs629367) | NSCLC | AC+CC vs. AA | Zhibin Hu (validation) | 2011 | Chinese | 355 | OS | 0.91 | 1.26 | 0.96 | [[5](#_ENREF_5)] |
| let-7a-2(rs629367) | NSCLC | AC vs. AA | Kyung Min Shin | 2016 | Korean | 761 | OS | 1.09 | 1.46 | 0.96 | [[3](#_ENREF_3)] |
| let-7a-2(rs629367) | NSCLC | CC vs. AA | Kyung Min Shin | 2016 | Korean | 761 | OS | 0.92 | 1.89 | 0.96 | [[3](#_ENREF_3)] |
| let-7a-2(rs629367) | NSCLC | AC+CC vs. AA | Kyung Min Shin | 2016 | Korean | 761 | OS | 1.07 | 1.42 | 0.96 | [[3](#_ENREF_3)] |
| let-7a-2(rs629367) | NSCLC | AA vs. AC vs. CC | Kyung Min Shin | 2016 | Korean | 761 | OS | 1.04 | 1.32 | 0.96 | [[3](#_ENREF_3)] |
| let-7a-2(rs629367) | NSCLC | AC vs. AA | Kyung Min Shin | 2016 | Korean | 761 | RFS | 1.03 | 1.29 | 0.96 | [[3](#_ENREF_3)] |
| let-7a-2(rs629367) | NSCLC | CC vs. AA | Kyung Min Shin | 2016 | Korean | 761 | RFS | 0.83 | 1.49 | 0.96 | [[3](#_ENREF_3)] |
| let-7a-2(rs629367) | NSCLC | AC+CC vs. AA | Kyung Min Shin | 2016 | Korean | 761 | RFS | 1.00 | 1.26 | 0.96 | [[3](#_ENREF_3)] |
| let-7a-2(rs629367) | NSCLC | AA vs. AC vs. CC | Kyung Min Shin | 2016 | Korean | 761 | RFS | 0.98 | 1.19 | 0.96 | [[3](#_ENREF_3)] |
| let-7a-2(rs629367) | GC | AC vs. AA | Qian Xu | 2014 | Chinese | 150 | OS | 4.48 | 12.60 | 0.96 | [[6](#_ENREF_6)] |
| let-7a-2(rs629367) | GC | CC vs. AA+AC | Qian Xu | 2014 | Chinese | 150 | OS | 4.69 | 11.95 | 0.96 | [[6](#_ENREF_6)] |
| let-7f-2(rs17276588) | NSCLC | GA vs. GG | Kyung Min Shin | 2016 | Korean | 761 | OS | 1.20 | 2.26 | 0.96 | [[3](#_ENREF_3)] |
| let-7f-2(rs17276588) | NSCLC | AA vs. GG | Kyung Min Shin | 2016 | Korean | 761 | OS | 0.97 | 1.33 | 0.96 | [[3](#_ENREF_3)] |
| let-7f-2(rs17276588) | NSCLC | GA+AA vs. GG | Kyung Min Shin | 2016 | Korean | 761 | OS | 1.00 | 1.34 | 0.96 | [[3](#_ENREF_3)] |
| let-7f-2(rs17276588) | NSCLC | GG vs. GA vs. AA | Kyung Min Shin | 2016 | Korean | 761 | OS | 0.99 | 1.16 | 0.96 | [[3](#_ENREF_3)] |
| let-7f-2(rs17276588) | NSCLC | GA vs. GG | Kyung Min Shin | 2016 | Korean | 761 | RFS | 1.10 | 1.71 | 0.96 | [[3](#_ENREF_3)] |
| let-7f-2(rs17276588) | NSCLC | AA vs. GG | Kyung Min Shin | 2016 | Korean | 761 | RFS | 1.08 | 1.39 | 0.96 | [[3](#_ENREF_3)] |
| let-7f-2(rs17276588) | NSCLC | GA+AA vs. GG | Kyung Min Shin | 2016 | Korean | 761 | RFS | 1.09 | 1.37 | 0.96 | [[3](#_ENREF_3)] |
| let-7f-2(rs17276588) | NSCLC | GG vs. GA vs. AA | Kyung Min Shin | 2016 | Korean | 761 | RFS | 1.04 | 1.18 | 0.96 | [[3](#_ENREF_3)] |
| miR-106b-25(rs999885) | HCC | AG vs. AA | Fuzhen Qi | 2014 | Chinese | 331 | OS | 0.79 | 1.05 | 0.96 | [[7](#_ENREF_7)] |
| miR-106b-25(rs999885) | HCC | GG vs. AA | Fuzhen Qi | 2014 | Chinese | 331 | OS | 0.41 | 1.10 | 0.96 | [[7](#_ENREF_7)] |
| miR-106b-25(rs999885) | HCC | AG+GG vs. AA | Fuzhen Qi | 2014 | Chinese | 331 | OS | 0.76 | 1.00 | 0.96 | [[7](#_ENREF_7)] |
| miR-107 (rs2296616) | GAC | TC vs. TT | Shizhi Wang | 2014 | Chinese | 940 | OS | 1.21 | 1.55 | 0.96 | [[8](#_ENREF_8)] |
| miR-107 (rs2296616) | GAC | CC vs. TT | Shizhi Wang | 2014 | Chinese | 940 | OS | 0.90 | 2.81 | 0.96 | [[8](#_ENREF_8)] |
| miR-107(rs78591545) | GAC | CT vs. TT | Shizhi Wang | 2014 | Chinese | 940 | OS | 1.04 | 1.27 | 0.96 | [[8](#_ENREF_8)] |
| miR-107(rs78591545) | GAC | TT vs. CC | Shizhi Wang | 2014 | Chinese | 940 | OS | 1.17 | 1.63 | 0.96 | [[8](#_ENREF_8)] |
| miR-107(rs11185777) | GAC | CT vs. CC | Shizhi Wang | 2014 | Chinese | 940 | OS | 1.02 | 1.28 | 0.96 | [[8](#_ENREF_8)] |
| miR-107(rs11185777) | GAC | TT vs. CC | Shizhi Wang | 2014 | Chinese | 940 | OS | 0.98 | 1.27 | 0.96 | [[8](#_ENREF_8)] |
| miR-1–2(rs9989532) | NSCLC | AG vs. AA | Zhibin Hu (screening) | 2011 | Chinese | 568 | OS | 1.44 | 1.95 | 0.96 | [[5](#_ENREF_5)] |
| miR-1–2(rs9989532) | NSCLC | GG vs. AA | Zhibin Hu (screening) | 2011 | Chinese | 568 | OS | 1.58 | 3.89 | 0.96 | [[5](#_ENREF_5)] |
| miR-1–2(rs9989532) | NSCLC | AG+GG vs. AA | Zhibin Hu (screening) | 2011 | Chinese | 568 | OS | 1.45 | 2.70 | 0.96 | [[5](#_ENREF_5)] |
| miR-1–2(rs9989532) | NSCLC | AG vs. AA | Zhibin Hu (validation) | 2011 | Chinese | 355 | OS | 0.76 | 1.23 | 0.96 | [[5](#_ENREF_5)] |
| miR-1–2(rs9989532) | NSCLC | GG vs. AA | Zhibin Hu (validation) | 2011 | Chinese | 355 | OS | 1.10 | 4.51 | 0.96 | [[5](#_ENREF_5)] |
| miR-1–2(rs9989532) | NSCLC | AG+GG vs. AA | Zhibin Hu (validation) | 2011 | Chinese | 355 | OS | 0.78 | 1.24 | 0.96 | [[5](#_ENREF_5)] |
| miR-124-2(rs298206) | NSCLC | AT vs. AA | Shuangshuang Wu | 2015 | Chinese | 1001 | OS | 1.30 | 1.56 | 0.96 | [[9](#_ENREF_9)] |
| miR-124-2(rs298206) | NSCLC | TT vs. AA | Shuangshuang Wu | 2015 | Chinese | 1001 | OS | 1.08 | 1.44 | 0.96 | [[9](#_ENREF_9)] |
| miR-124-2(rs298206) | NSCLC | AT+TT vs. AA | Shuangshuang Wu | 2015 | Chinese | 1001 | OS | 1.25 | 1.49 | 0.96 | [[9](#_ENREF_9)] |
| miR-124-3(rs6122390) | NSCLC | GA vs. GG | Shuangshuang Wu | 2015 | Chinese | 1001 | OS | 1.27 | 1.53 | 0.96 | [[9](#_ENREF_9)] |
| miR-124-3(rs6122390) | NSCLC | AA vs. GG | Shuangshuang Wu | 2015 | Chinese | 1001 | OS | 1.42 | 1.86 | 0.96 | [[9](#_ENREF_9)] |
| miR-124-3(rs6122390) | NSCLC | GA+AA vs. GG | Shuangshuang Wu | 2015 | Chinese | 1001 | OS | 1.30 | 1.55 | 0.96 | [[9](#_ENREF_9)] |
| miR-125a(rs12976445) | Breast cancer | CT vs. CC | Lianghe Jiao | 2014 | Chinese | 196 | OS | 1.62 | 2.96 | 0.96 | [[10](#_ENREF_10)] |
| miR-125a(rs12976445) | Breast cancer | TT vs. CC | Lianghe Jiao | 2014 | Chinese | 196 | OS | 3.96 | 14.69 | 0.96 | [[10](#_ENREF_10)] |
| miR-125a(rs12976445) | Breast cancer | CT+TT vs. CC | Lianghe Jiao | 2014 | Chinese | 196 | OS | 1.70 | 3.04 | 0.96 | [[10](#_ENREF_10)] |
| miR-125a(rs12976445) | Breast cancer | TT vs. CT+CC | Lianghe Jiao | 2014 | Chinese | 196 | OS | 2.13 | 7.22 | 0.96 | [[10](#_ENREF_10)] |
| miR-125b(rs2241490) | NSCLC | GA vs. GG | Zhibin Hu (screening) | 2011 | Chinese | 568 | OS | 0.94 | 1.20 | 0.96 | [[5](#_ENREF_5)] |
| miR-125b(rs2241490) | NSCLC | AA vs. GG | Zhibin Hu (screening) | 2011 | Chinese | 568 | OS | 1.61 | 2.36 | 0.96 | [[5](#_ENREF_5)] |
| miR-125b(rs2241490) | NSCLC | AA vs. GG+GA | Zhibin Hu (screening) | 2011 | Chinese | 568 | OS | 1.65 | 2.39 | 0.96 | [[5](#_ENREF_5)] |
| miR-125b(rs2241490) | NSCLC | GA vs. GG | Zhibin Hu (validation) | 2011 | Chinese | 355 | OS | 1.18 | 1.64 | 0.96 | [[5](#_ENREF_5)] |
| miR-125b(rs2241490) | NSCLC | AA vs. GG | Zhibin Hu (validation) | 2011 | Chinese | 355 | OS | 1.31 | 2.24 | 0.96 | [[5](#_ENREF_5)] |
| miR-125b(rs2241490) | NSCLC | AA vs. GG+GA | Zhibin Hu (validation) | 2011 | Chinese | 355 | OS | 1.20 | 2.01 | 0.96 | [[5](#_ENREF_5)] |
| miR-1268a (rs28599926) | HCC | CT+TT vs. CC | Xi-Dai Long | 2016 | Chinese | 1299 | OS | 2.12 | 2.41 | 0.96 | [[11](#_ENREF_11)] |
| miR-1268a (rs28599926) | HCC | CT+TT vs. CC | Xi-Dai Long | 2016 | Chinese | 1299 | RFS | 2.86 | 3.43 | 0.96 | [[11](#_ENREF_11)] |
| miR-145(rs353291) | NSCLC | AG vs. AA | Zhibin Hu (screening) | 2011 | Chinese | 568 | OS | 0.74 | 0.94 | 0.96 | [[5](#_ENREF_5)] |
| miR-145(rs353291) | NSCLC | GG vs. AA | Zhibin Hu (screening) | 2011 | Chinese | 568 | OS | 0.78 | 1.08 | 0.96 | [[5](#_ENREF_5)] |
| miR-145(rs353291) | NSCLC | AG+GG vs. AA | Zhibin Hu (screening) | 2011 | Chinese | 568 | OS | 0.75 | 0.94 | 0.96 | [[5](#_ENREF_5)] |
| miR-145(rs353291) | NSCLC | AG vs. AA | Zhibin Hu (validation) | 2011 | Chinese | 355 | OS | 1.54 | 2.21 | 0.96 | [[5](#_ENREF_5)] |
| miR-145(rs353291) | NSCLC | GG vs. AA | Zhibin Hu (validation) | 2011 | Chinese | 355 | OS | 1.23 | 2.03 | 0.96 | [[5](#_ENREF_5)] |
| miR-145(rs353291) | NSCLC | AG+GG vs. AA | Zhibin Hu (validation) | 2011 | Chinese | 355 | OS | 1.47 | 2.07 | 0.96 | [[5](#_ENREF_5)] |
| mir-146(rs2910164) | SCCOP | GG vs. CG+CC | Xingming Chen | 2016 | Chinese | 1008 | DFS | 0.60 | 0.90 | 0.96 | [[12](#_ENREF_12)] |
| mir-146a (rs2910164) | NSCLC | AC vs. CC | Kyong-Ah Yoon | 2012 | Korean | 388 | RFS | 0.52 | 0.89 | 0.96 | [[13](#_ENREF_13)] |
| mir-146a (rs2910164) | NSCLC | AA vs. CC | Kyong-Ah Yoon | 2012 | Korean | 388 | RFS | 0.53 | 1.13 | 0.96 | [[13](#_ENREF_13)] |
| mir-146a (rs2910164) | NSCLC | AC+AA vs. CC | Kyong-Ah Yoon | 2012 | Korean | 388 | RFS | 0.52 | 0.85 | 0.96 | [[13](#_ENREF_13)] |
| mir-146a(rs2910164) | CRC | GC vs. CC | MOON JU JANG | 2011 | Korean | 407 | OS | 1.20 | 1.94 | 0.96 | [[14](#_ENREF_14)] |
| mir-146a(rs2910164) | CRC | GG vs. CC | MOON JU JANG | 2011 | Korean | 407 | OS | 0.85 | 1.90 | 0.96 | [[14](#_ENREF_14)] |
| mir-146a(rs2910164) | CRC | GC+GG vs. CC | MOON JU JANG | 2011 | Korean | 407 | OS | 1.14 | 1.82 | 0.96 | [[14](#_ENREF_14)] |
| mir-146a(rs2910164) | CRC | GG vs. GC+CC | MOON JU JANG | 2011 | Korean | 407 | OS | 0.76 | 1.57 | 0.96 | [[14](#_ENREF_14)] |
| mir-146a(rs2910164) | CRC | GC vs. CC | MOON JU JANG | 2011 | Korean | 407 | RFS | 1.13 | 1.81 | 0.96 | [[14](#_ENREF_14)] |
| mir-146a(rs2910164) | CRC | GG vs. CC | MOON JU JANG | 2011 | Korean | 407 | RFS | 1.49 | 2.97 | 0.96 | [[14](#_ENREF_14)] |
| mir-146a(rs2910164) | CRC | GC+GG vs. CC | MOON JU JANG | 2011 | Korean | 407 | RFS | 1.19 | 1.87 | 0.96 | [[14](#_ENREF_14)] |
| mir-146a(rs2910164) | CRC | GG vs. GC+CC | MOON JU JANG | 2011 | Korean | 407 | RFS | 1.39 | 2.57 | 0.96 | [[14](#_ENREF_14)] |
| miR-146a(rs2910164) | CRC | GC vs. GG | Jinliang Xing | 2012 | Chinese | 408 | OS | 1.13 | 1.89 | 0.96 | [[15](#_ENREF_15)] |
| miR-146a(rs2910164) | CRC | GG vs. CC | Jinliang Xing | 2012 | Chinese | 408 | OS | 1.05 | 2.04 | 0.96 | [[15](#_ENREF_15)] |
| miR-146a(rs2910164) | CRC | GG vs. GC+CC | Jinliang Xing | 2012 | Chinese | 408 | OS | 0.93 | 1.52 | 0.96 | [[15](#_ENREF_15)] |
| miR-146a(rs2910164) | CRC | GC+GG vs. CC | Jinliang Xing | 2012 | Chinese | 408 | OS | 1.14 | 2.00 | 0.96 | [[15](#_ENREF_15)] |
| miR-146a(rs2910164) | CRC | GC vs. GG | Jinliang Xing | 2012 | Chinese | 408 | RFS | 1.10 | 1.69 | 0.96 | [[15](#_ENREF_15)] |
| miR-146a(rs2910164) | CRC | GG vs. CC | Jinliang Xing | 2012 | Chinese | 408 | RFS | 1.06 | 1.85 | 0.96 | [[15](#_ENREF_15)] |
| miR-146a(rs2910164) | CRC | GG vs. GC+CC | Jinliang Xing | 2012 | Chinese | 408 | RFS | 0.94 | 1.43 | 0.96 | [[15](#_ENREF_15)] |
| miR-146a(rs2910164) | CRC | CC vs. GC+GG | Jinliang Xing | 2012 | Chinese | 408 | RFS | 0.89 | 1.46 | 0.96 | [[15](#_ENREF_15)] |
| miR-146a(rs2910164) | NSCLC | CG vs. CC | Mi Jeong Hong | 2013 | Korean | 363 | OS | 0.88 | 1.25 | 0.96 | [[16](#_ENREF_16)] |
| miR-146a(rs2910164) | NSCLC | GG vs. CC | Mi Jeong Hong | 2013 | Korean | 363 | OS | 0.65 | 1.18 | 0.96 | [[16](#_ENREF_16)] |
| miR-146a(rs2910164) | NSCLC | GG vs. CC+CG | Mi Jeong Hong | 2013 | Korean | 363 | OS | 0.70 | 1.22 | 0.96 | [[16](#_ENREF_16)] |
| miR-146a(rs2910164) | NSCLC | CG vs. CC | Mi Jeong Hong | 2013 | Korean | 363 | DFS | 0.94 | 1.29 | 0.96 | [[16](#_ENREF_16)] |
| miR-146a(rs2910164) | NSCLC | GG vs. CC | Mi Jeong Hong | 2013 | Korean | 363 | DFS | 0.74 | 1.22 | 0.96 | [[16](#_ENREF_16)] |
| miR-146a(rs2910164) | NSCLC | GG vs. CC+CG | Mi Jeong Hong | 2013 | Korean | 363 | DFS | 0.76 | 1.23 | 0.96 | [[16](#_ENREF_16)] |
| mir-146a(rs2910164) | NSCLC | CG vs. CC | Zhibin Hu | 2008 | Chinese | 663 | OS | 1.05 | 1.47 | 0.96 | [[17](#_ENREF_17)] |
| mir-146a(rs2910164) | NSCLC | GG vs. CC | Zhibin Hu | 2008 | Chinese | 663 | OS | 1.28 | 1.82 | 0.96 | [[17](#_ENREF_17)] |
| mir-146a(rs2910164) | SCCNOP | GG vs. CG+CC | Chengyuan Wang | 2016 | Chinese | 996 | OS | 0.90 | 1.20 | 0.96 | [[18](#_ENREF_18)] |
| mir-146a(rs2910164) | SCCNOP | GG vs. CG+CC | Chengyuan Wang | 2016 | Chinese | 996 | DFS | 0.90 | 1.20 | 0.96 | [[18](#_ENREF_18)] |
| miR-146a(rs2910164) | NSCLC | CG vs. CC | Xia Lingzi | 2016 | Chinese | 584 | OS | 1.00 | 1.22 | 0.96 | [[19](#_ENREF_19)] |
| miR-146a(rs2910164) | NSCLC | GG vs. CC | Xia Lingzi | 2016 | Chinese | 584 | OS | 0.97 | 1.27 | 0.96 | [[19](#_ENREF_19)] |
| mir-146a(rs2910164) | SCCOP | GG vs. CG+CC | Xiaoxiang Guan | 2013 | Chinese | 281 | OS | 0.50 | 1.00 | 0.96 | [[20](#_ENREF_20)] |
| mir-146a(rs2910164) | SCCOP | GG vs. CG+CC | Xiaoxiang Guan | 2013 | Chinese | 281 | DFS | 0.30 | 0.80 | 0.96 | [[20](#_ENREF_20)] |
| miR-146a(rs2910164) | CRC | CC vs. CG+GG | YEE SOO CHA | 2013 | Korean | 343 | RFS | 2.12 | 3.57 | 0.96 | [[21](#_ENREF_21)] |
| miR-146a(rs2910164) | Bladder cancer | GG vs. CG+CC | Meilin Wang | 2012 | Chinese | 74 | RFS | 0.58 | 0.94 | 0.96 | [[22](#_ENREF_22)] |
| miR-146a(rs2910164) | GC | GG vs. CG+CC | Jing Jiang | 2016 | Chinese | 838 | OS | 1.36 | 1.78 | 0.96 | [[23](#_ENREF_23)] |
| miR-146a(rs2910164) | GC | CG vs. CC | Dae Ho Ahn | 2013 | Korean | 160 | OS | 0.70 | 1.50 | 0.96 | [[24](#_ENREF_24)] |
| miR-146a(rs2910164) | GC | GG vs. CC | Dae Ho Ahn | 2013 | Korean | 160 | OS | 0.40 | 1.30 | 0.96 | [[24](#_ENREF_24)] |
| miR-146a(rs2910164) | GC | CG+GG vs. CC | Dae Ho Ahn | 2013 | Korean | 160 | OS | 0.60 | 1.30 | 0.96 | [[24](#_ENREF_24)] |
| miR-146a(rs2910164) | HCC | GC vs. CC | Won Hee Kim | 2012 | Korean | 67 | OS | 0.90 | 1.72 | 0.96 | [[25](#_ENREF_25)] |
| miR-146a(rs2910164) | HCC | GG vs.CC | Won Hee Kim | 2012 | Korean | 67 | OS | 1.46 | 3.73 | 0.96 | [[25](#_ENREF_25)] |
| miR-146a(rs2910164) | HCC | GC+GG vs. CC | Won Hee Kim | 2012 | Korean | 67 | OS | 0.96 | 1.80 | 0.96 | [[25](#_ENREF_25)] |
| miR-146a(rs2910164) | HCC | GG vs. GC+CC | Won Hee Kim | 2012 | Korean | 67 | OS | 1.57 | 3.54 | 0.96 | [[25](#_ENREF_25)] |
| miR-146a(rs2910164) | ESCC | GC vs. GG | Meenakshi Umar | 2013 | India | 153 | OS | 0.84 | 1.28 | 0.96 | [[26](#_ENREF_26)] |
| miR-146a(rs2910164) | ESCC | GG vs. CC | Meenakshi Umar | 2013 | India | 153 | OS | 2.22 | 5.88 | 0.96 | [[26](#_ENREF_26)] |
| miR-146a(rs2910164) | PTC | CC vs. GC+GG | Marta Kotlarek | 2018 | Polish | 315 | OS | 6.21 | 27.93 | 1.38 | [[27](#_ENREF_27)] |
| mir-149(rs2292832) | CRC | TC vs. TT | MOON JU JANG | 2011 | Korean | 407 | OS | 1.01 | 1.59 | 0.96 | [[14](#_ENREF_14)] |
| mir-149(rs2292832) | CRC | CC vs. TT | MOON JU JANG | 2011 | Korean | 407 | OS | 0.99 | 2.06 | 0.96 | [[14](#_ENREF_14)] |
| mir-149(rs2292832) | CRC | TC+CC vs. TT | MOON JU JANG | 2011 | Korean | 407 | OS | 1.00 | 1.54 | 0.96 | [[14](#_ENREF_14)] |
| mir-149(rs2292832) | CRC | CC vs. TC+TT | MOON JU JANG | 2011 | Korean | 407 | OS | 0.99 | 2.00 | 0.96 | [[14](#_ENREF_14)] |
| mir-149(rs2292832) | CRC | TC vs. TT | MOON JU JANG | 2011 | Korean | 407 | RFS | 1.20 | 1.90 | 0.96 | [[14](#_ENREF_14)] |
| mir-149(rs2292832) | CRC | CC vs. TT | MOON JU JANG | 2011 | Korean | 407 | RFS | 0.90 | 1.91 | 0.96 | [[14](#_ENREF_14)] |
| mir-149(rs2292832) | CRC | TC+CC vs. TT | MOON JU JANG | 2011 | Korean | 407 | RFS | 1.13 | 1.76 | 0.96 | [[14](#_ENREF_14)] |
| mir-149(rs2292832) | CRC | CC vs. TC+TT | MOON JU JANG | 2011 | Korean | 407 | RFS | 0.82 | 1.67 | 0.96 | [[14](#_ENREF_14)] |
| miR-149(rs2292832) | NSCLC | TC vs. TT | Mi Jeong Hong | 2013 | Korean | 363 | OS | 0.66 | 0.94 | 0.96 | [[16](#_ENREF_16)] |
| miR-149(rs2292832) | NSCLC | CC vs. TT | Mi Jeong Hong | 2013 | Korean | 363 | OS | 0.65 | 1.20 | 0.96 | [[16](#_ENREF_16)] |
| miR-149(rs2292832) | NSCLC | TC+CC vs. TT | Mi Jeong Hong | 2013 | Korean | 363 | OS | 0.66 | 0.92 | 0.96 | [[16](#_ENREF_16)] |
| miR-149(rs2292832) | NSCLC | TC vs. TT | Mi Jeong Hong | 2013 | Korean | 363 | DFS | 0.66 | 0.90 | 0.96 | [[16](#_ENREF_16)] |
| miR-149(rs2292832) | NSCLC | CC vs. TT | Mi Jeong Hong | 2013 | Korean | 363 | DFS | 0.59 | 1.01 | 0.96 | [[16](#_ENREF_16)] |
| miR-149(rs2292832) | NSCLC | TC+CC vs. TT | Mi Jeong Hong | 2013 | Korean | 363 | DFS | 0.64 | 0.87 | 0.96 | [[16](#_ENREF_16)] |
| mir-149(rs2292832) | NSCLC | CT vs. CC | Zhibin Hu | 2008 | Chinese | 663 | OS | 1.03 | 1.54 | 0.96 | [[17](#_ENREF_17)] |
| mir-149(rs2292832) | NSCLC | CC vs. TT | Zhibin Hu | 2008 | Chinese | 663 | OS | 0.76 | 1.12 | 0.96 | [[17](#_ENREF_17)] |
| mir-149(rs2292832) | NSCLC | CT+CC vs. TT | Zhibin Hu | 2008 | Chinese | 663 | OS | 0.78 | 0.99 | 0.96 | [[17](#_ENREF_17)] |
| mir-149(rs2292832) | SCCNOP | CC vs. CT+TT | Chengyuan Wang | 2016 | Chinese | 996 | OS | 0.60 | 0.80 | 0.96 | [[18](#_ENREF_18)] |
| mir-149(rs2292832) | SCCNOP | CC vs. CT+TT | Chengyuan Wang | 2016 | Chinese | 996 | DFS | 0.70 | 0.70 | 0.96 | [[18](#_ENREF_18)] |
| miR-149(rs2292832) | NSCLC | CT vs. TT | Xia Lingzi | 2016 | Chinese | 584 | OS | 0.78 | 0.98 | 0.96 | [[19](#_ENREF_19)] |
| miR-149(rs2292832) | NSCLC | CC vs. TT | Xia Lingzi | 2016 | Chinese | 584 | OS | 0.61 | 0.87 | 0.96 | [[19](#_ENREF_19)] |
| miR-149(rs2292832) | NSCLC | CC+CT vs. TT | Xia Lingzi | 2016 | Chinese | 584 | OS | 0.74 | 0.90 | 0.96 | [[19](#_ENREF_19)] |
| miR-149(rs2292832) | NSCLC | CC vs. CT+TT | Xia Lingzi | 2016 | Chinese | 584 | OS | 0.64 | 0.92 | 0.96 | [[19](#_ENREF_19)] |
| mir-149(rs2292832) | SCCOP | CC vs. CT+TT | Xiaoxiang Guan | 2013 | Chinese | 281 | OS | 0.60 | 1.20 | 0.96 | [[20](#_ENREF_20)] |
| mir-149(rs2292832) | SCCOP | CC vs. CT+TT | Xiaoxiang Guan | 2013 | Chinese | 281 | DFS | 1.30 | 3.20 | 0.96 | [[20](#_ENREF_20)] |
| miR-149(rs2292832) | SCCOP | CC vs. CT+TT | Xingming Chen | 2016 | Chinese | 1008 | DFS | 1.00 | 1.30 | 0.96 | [[12](#_ENREF_12)] |
| miR-149(rs2292832) | GC | TC vs. TT | Dae Ho Ahn | 2013 | Korean | 160 | OS | 1.00 | 2.30 | 0.96 | [[24](#_ENREF_24)] |
| miR-149(rs2292832) | GC | CC vs. TT | Dae Ho Ahn | 2013 | Korean | 160 | OS | 2.00 | 5.80 | 0.96 | [[24](#_ENREF_24)] |
| miR-149(rs2292832) | GC | TC+CC vs. TT | Dae Ho Ahn | 2013 | Korean | 160 | OS | 1.20 | 2.50 | 0.96 | [[24](#_ENREF_24)] |
| miR-149(rs2292832) | HCC | CT vs. TT | Won Hee Kim | 2012 | Korean | 67 | OS | 0.58 | 1.12 | 0.96 | [[25](#_ENREF_25)] |
| miR-149(rs2292832) | HCC | CC vs. TT | Won Hee Kim | 2012 | Korean | 67 | OS | 0.18 | 0.78 | 0.96 | [[25](#_ENREF_25)] |
| miR-149(rs2292832) | HCC | CT+CC vs. TT | Won Hee Kim | 2012 | Korean | 67 | OS | 0.50 | 0.95 | 0.96 | [[25](#_ENREF_25)] |
| miR-149(rs2292832) | HCC | CC vs. CT+TT | Won Hee Kim | 2012 | Korean | 67 | OS | 0.24 | 1.02 | 0.96 | [[25](#_ENREF_25)] |
| miR-155(rs767649) | NSCLC | AT vs. AA | Kaipeng Xie | 2015 | Chinese | 1001 | OS | 1.10 | 1.34 | 0.96 | [[28](#_ENREF_28)] |
| miR-155(rs767649) | NSCLC | TT vs. AA | Kaipeng Xie | 2015 | Chinese | 1001 | OS | 1.38 | 1.77 | 0.96 | [[28](#_ENREF_28)] |
| miR-155(rs767649) | NSCLC | AT+AA vs. AA | Kaipeng Xie | 2015 | Chinese | 1001 | OS | 1.17 | 1.41 | 0.96 | [[28](#_ENREF_28)] |
| miR-155(rs767649) | NSCLC | TT vs. AT+AA | Kaipeng Xie | 2015 | Chinese | 1001 | OS | 1.31 | 1.62 | 0.96 | [[28](#_ENREF_28)] |
| miR-16-1/15a(rs9535416) | Breast cancer | AG+AA vs. GG | Jeannette T. Bensen | 2013 | American | 1946 | OS | 1.12 | 1.44 | 0.96 | [[29](#_ENREF_29)] |
| mir-182(rs129197463) | NSCLC | TC vs. CC | Yang Zhao | 2014 | American | 452 | OS | 1.87 | 2.78 | 0.96 | [[30](#_ENREF_30)] |
| mir-182(rs129197463) | NSCLC | TT vs. CC | Yang Zhao | 2014 | American | 452 | OS | 29.29 | 242.65 | 0.96 | [[30](#_ENREF_30)] |
| miR-184(rs919968) | NSCLC | CA vs. CC | Shuangshuang Wu | 2015 | Chinese | 1001 | OS | 1.03 | 1.26 | 0.96 | [[9](#_ENREF_9)] |
| miR-184(rs919968) | NSCLC | AA vs. CC | Shuangshuang Wu | 2015 | Chinese | 1001 | OS | 1.34 | 1.69 | 0.96 | [[9](#_ENREF_9)] |
| miR-184(rs919968) | NSCLC | CA+AA vs. CC | Shuangshuang Wu | 2015 | Chinese | 1001 | OS | 1.12 | 1.34 | 0.96 | [[9](#_ENREF_9)] |
| miR-193b(rs30236) | NSCLC | GA vs. GG | Zhibin Hu (screening) | 2011 | Chinese | 568 | OS | 1.14 | 1.44 | 0.96 | [[5](#_ENREF_5)] |
| miR-193b(rs30236) | NSCLC | AA vs. GG | Zhibin Hu (screening) | 2011 | Chinese | 568 | OS | 1.72 | 2.50 | 0.96 | [[5](#_ENREF_5)] |
| miR-193b(rs30236) | NSCLC | AA vs. GA+GG | Zhibin Hu (screening) | 2011 | Chinese | 568 | OS | 1.62 | 2.30 | 0.96 | [[5](#_ENREF_5)] |
| miR-193b(rs30236) | NSCLC | GA vs. GG | Zhibin Hu (validation) | 2011 | Chinese | 355 | OS | 0.91 | 1.28 | 0.96 | [[5](#_ENREF_5)] |
| miR-193b(rs30236) | NSCLC | AA vs. GG | Zhibin Hu (validation) | 2011 | Chinese | 355 | OS | 0.98 | 1.65 | 0.96 | [[5](#_ENREF_5)] |
| miR-193b(rs30236) | NSCLC | AA vs. GA+GG | Zhibin Hu (validation) | 2011 | Chinese | 355 | OS | 1.02 | 1.68 | 0.96 | [[5](#_ENREF_5)] |
| miR-196(rs11614913) | SCCOP | CC vs. CT+TT | Xingming Chen | 2016 | Chinese | 1008 | DFS | 2.10 | 2.80 | 0.96 | [[12](#_ENREF_12)] |
| miR-196a(rs11614913) | Early Breast cancer | CC vs. TT+TC | SOO JUNG LEE | 2014 | Korean | 452 | OS | 0.94 | 1.88 | 0.96 | [[31](#_ENREF_31)] |
| miR-196a(rs11614913) | Early Breast cancer | CC vs. TT+TC | SOO JUNG LEE | 2014 | Korean | 452 | DFS | 2.35 | 4.11 | 0.96 | [[31](#_ENREF_31)] |
| miR-196a(rs11614913) | Early Breast cancer | CC vs. TT+TC | SOO JUNG LEE | 2014 | Korean | 452 | Distant DFS | 2.40 | 4.66 | 0.96 | [[31](#_ENREF_31)] |
| miR-196a(rs11614913) | NSCLC | CT vs. CC | Mi Jeong Hong | 2013 | Korean | 363 | OS | 0.67 | 0.98 | 0.96 | [[16](#_ENREF_16)] |
| miR-196a(rs11614913) | NSCLC | TT vs. CC | Mi Jeong Hong | 2013 | Korean | 363 | OS | 0.77 | 1.23 | 0.96 | [[16](#_ENREF_16)] |
| miR-196a(rs11614913) | NSCLC | CT+TT vs. CC | Mi Jeong Hong | 2013 | Korean | 363 | OS | 0.70 | 0.99 | 0.96 | [[16](#_ENREF_16)] |
| miR-196a(rs11614913) | NSCLC | CT vs. CC | Mi Jeong Hong | 2013 | Korean | 363 | DFS | 0.68 | 0.95 | 0.96 | [[16](#_ENREF_16)] |
| miR-196a(rs11614913) | NSCLC | TT vs. CC | Mi Jeong Hong | 2013 | Korean | 363 | DFS | 0.61 | 0.93 | 0.96 | [[16](#_ENREF_16)] |
| miR-196a(rs11614913) | NSCLC | CT+TT vs. CC | Mi Jeong Hong | 2013 | Korean | 363 | DFS | 0.66 | 0.90 | 0.96 | [[16](#_ENREF_16)] |
| miR-196a(rs11614913) | RCC | TC vs. TT | Mulong Du | 2014 | Chinese | 311 | OS | 0.48 | 1.14 | 0.96 | [[32](#_ENREF_32)] |
| miR-196a(rs11614913) | RCC | CC vs. TT | Mulong Du | 2014 | Chinese | 311 | OS | 0.32 | 1.67 | 0.96 | [[32](#_ENREF_32)] |
| miR-196a(rs11614913) | RCC | TC+CC vs. TT | Mulong Du | 2014 | Chinese | 311 | OS | 0.40 | 0.89 | 0.96 | [[32](#_ENREF_32)] |
| miR-196a(rs11614913) | RCC | CC vs. TC+TT | Mulong Du | 2014 | Chinese | 311 | OS | 0.36 | 1.62 | 0.96 | [[32](#_ENREF_32)] |
| mir-196a-2 (rs11614913) | HNSCC | TT vs. TC+CC | Brock C. Christensen | 2010 | American | 323 | OS | 1.30 | 2.20 | 0.96 | [[33](#_ENREF_33)] |
| mir-196a2(rs11614913) | CRC | TC vs. TT | MOON JU JANG | 2011 | Korean | 407 | OS | 1.08 | 1.85 | 0.96 | [[14](#_ENREF_14)] |
| mir-196a2(rs11614913) | CRC | CC vs. TT | MOON JU JANG | 2011 | Korean | 407 | OS | 1.07 | 1.99 | 0.96 | [[14](#_ENREF_14)] |
| mir-196a2(rs11614913) | CRC | TC+CC vs. TT | MOON JU JANG | 2011 | Korean | 407 | OS | 1.08 | 1.79 | 0.96 | [[14](#_ENREF_14)] |
| mir-196a2(rs11614913) | CRC | CC vs. TC+TT | MOON JU JANG | 2011 | Korean | 407 | OS | 1.02 | 1.69 | 0.96 | [[14](#_ENREF_14)] |
| mir-196a2(rs11614913) | CRC | TC vs. TT | MOON JU JANG | 2011 | Korean | 407 | RFS | 0.76 | 1.31 | 0.96 | [[14](#_ENREF_14)] |
| mir-196a2(rs11614913) | CRC | CC vs. TT | MOON JU JANG | 2011 | Korean | 407 | RFS | 1.00 | 1.79 | 0.96 | [[14](#_ENREF_14)] |
| mir-196a2(rs11614913) | CRC | TC+CC vs. TT | MOON JU JANG | 2011 | Korean | 407 | RFS | 0.85 | 1.39 | 0.96 | [[14](#_ENREF_14)] |
| mir-196a2(rs11614913) | CRC | CC vs. TC+TT | MOON JU JANG | 2011 | Korean | 407 | RFS | 1.19 | 1.92 | 0.96 | [[14](#_ENREF_14)] |
| mir-196a2(rs11614913) | ESCC | CT vs. CC | Pei-Wen Yang | 2014 | Chinese | 129 | OS | 0.80 | 1.55 | 0.96 | [[34](#_ENREF_34)] |
| mir-196a2(rs11614913) | ESCC | TT vs. CC | Pei-Wen Yang | 2014 | Chinese | 129 | OS | 0.85 | 1.74 | 0.96 | [[34](#_ENREF_34)] |
| mir-196a2(rs11614913) | ESCC | CT vs. CC | Pei-Wen Yang | 2014 | Chinese | 129 | RFS | 0.97 | 1.72 | 0.96 | [[34](#_ENREF_34)] |
| mir-196a2(rs11614913) | ESCC | TT vs. CC | Pei-Wen Yang | 2014 | Chinese | 129 | RFS | 1.01 | 1.88 | 0.96 | [[34](#_ENREF_34)] |
| miR-196a2(rs11614913) | NSCLC | CT vs. TT | Kyong-Ah Yoon | 2012 | Korean | 388 | RFS | 0.66 | 1.14 | 0.96 | [[13](#_ENREF_13)] |
| miR-196a2(rs11614913) | NSCLC | CC vs. TT | Kyong-Ah Yoon | 2012 | Korean | 388 | RFS | 0.67 | 1.29 | 0.96 | [[13](#_ENREF_13)] |
| miR-196a2(rs11614913) | NSCLC | CT+CC vs. TT | Kyong-Ah Yoon | 2012 | Korean | 388 | RFS | 0.67 | 1.10 | 0.96 | [[13](#_ENREF_13)] |
| mir-196a2(rs11614913) | NSCLC | CT vs. TT | Zhibin Hu | 2008 | Chinese | 663 | OS | 1.09 | 1.46 | 0.96 | [[17](#_ENREF_17)] |
| mir-196a2(rs11614913) | NSCLC | CC vs. TT | Zhibin Hu | 2008 | Chinese | 663 | OS | 1.86 | 2.62 | 0.96 | [[17](#_ENREF_17)] |
| mir-196a2(rs11614913) | NSCLC | CC vs. CT+TT | Zhibin Hu | 2008 | Chinese | 663 | OS | 1.76 | 2.33 | 0.96 | [[17](#_ENREF_17)] |
| mir-196a2(rs11614913) | SCCNOP | CC vs. CT+TT | Chengyuan Wang | 2016 | Chinese | 996 | OS | 1.10 | 1.30 | 0.96 | [[18](#_ENREF_18)] |
| mir-196a2(rs11614913) | SCCNOP | CC vs. CT+TT | Chengyuan Wang | 2016 | Chinese | 996 | DFS | 1.10 | 1.30 | 0.96 | [[18](#_ENREF_18)] |
| miR-196a2(rs11614913) | NSCLC | CT vs. TT | Xia Lingzi | 2016 | Chinese | 584 | OS | 0.98 | 1.21 | 0.96 | [[19](#_ENREF_19)] |
| miR-196a2(rs11614913) | NSCLC | CC vs. TT | Xia Lingzi | 2016 | Chinese | 584 | OS | 0.85 | 1.12 | 0.96 | [[19](#_ENREF_19)] |
| miR-196a2(rs11614913) | OSCC | CT+CC vs. TT | Chung-Ji Liu | 2013 | Chinese | 315 | OS | 2.05 | 3.37 | 0.96 | [[35](#_ENREF_35)] |
| mir-196a2(rs11614913) | SCCOP | CT+TT vs. CC | Xiaoxiang Guan | 2013 | Chinese | 281 | OS | 0.40 | 0.80 | 0.96 | [[20](#_ENREF_20)] |
| mir-196a2(rs11614913) | SCCOP | CT+TT vs. CC | Xiaoxiang Guan | 2013 | Chinese | 281 | DFS | 0.30 | 0.70 | 0.96 | [[20](#_ENREF_20)] |
| miR-196a2(rs11614913) | GC | CC vs. CT+TT | Shizhi Wang | 2013 | Chinese | 940 | OS | 0.72 | 0.95 | 0.96 | [[36](#_ENREF_36)] |
| miR-196a2(rs11614913) | NCGC | CC vs. CT+TT | Shizhi Wang | 2013 | Chinese | 940 | OS | 0.57 | 0.83 | 0.96 | [[36](#_ENREF_36)] |
| miR-196a2(rs11614913) | GC | TC vs. TT | Dae Ho Ahn | 2013 | Korean | 160 | OS | 1.40 | 3.60 | 0.96 | [[24](#_ENREF_24)] |
| miR-196a2(rs11614913) | GC | CC vs. TT | Dae Ho Ahn | 2013 | Korean | 160 | OS | 1.80 | 5.00 | 0.96 | [[24](#_ENREF_24)] |
| miR-196a2(rs11614913) | GC | TC+CC vs. TT | Dae Ho Ahn | 2013 | Korean | 160 | OS | 1.60 | 3.70 | 0.96 | [[24](#_ENREF_24)] |
| miR-196a2(rs11614913) | HCC | CT vs. TT | Won Hee Kim | 2012 | Korean | 67 | OS | 1.45 | 2.76 | 0.96 | [[25](#_ENREF_25)] |
| miR-196a2(rs11614913) | HCC | CC vs. TT | Won Hee Kim | 2012 | Korean | 67 | OS | 0.99 | 2.27 | 0.96 | [[25](#_ENREF_25)] |
| miR-196a2(rs11614913) | HCC | CT+CC vs. TT | Won Hee Kim | 2012 | Korean | 67 | OS | 1.30 | 2.41 | 0.96 | [[25](#_ENREF_25)] |
| miR-196a2(rs11614913) | HCC | CC vs. CT+TT | Won Hee Kim | 2012 | Korean | 67 | OS | 0.79 | 1.61 | 0.96 | [[25](#_ENREF_25)] |
| miR-196a2(rs11614913) | HCC | TT vs. CT+CC | Juan Li | 2016 | Chinese | 109 | OS | 0.69 | 0.96 | 0.96 | [[37](#_ENREF_37)] |
| miR-196a2(rs11614913) | ESCC | CT vs. CC | Meenakshi Umar | 2013 | India | 153 | OS | 0.90 | 1.36 | 0.96 | [[26](#_ENREF_26)] |
| miR-196a2(rs11614913) | ESCC | TT vs. CC | Meenakshi Umar | 2013 | India | 153 | OS | 1.04 | 2.61 | 0.96 | [[26](#_ENREF_26)] |
| 196a2(rs11614913) | Brain cancer | TC vs. TT | JAEJOON LIM | 2018 | Korean | 179 | OS | 2.09 | 3.82 | 1.15 | [[38](#_ENREF_38)] |
| 196a2(rs11614913) | Brain cancer | TC+CC vs. TT | JAEJOON LIM | 2018 | Korean | 179 | OS | 1.81 | 3.14 | 1.04 | [[38](#_ENREF_38)] |
| miR-206(rs6920648) | Breast cancer | AG+GG vs. AA | Jeannette T. Bensen | 2013 | American | 1946 | OS | 0.79 | 1.00 | 0.96 | [[29](#_ENREF_29)] |
| miR-218 (rs11134527 ) | GC | GA+GG vs. AA | Yanhua Wu | 2017 | Chinese | 735 | OS | 0.73 | 0.93 | 0.96 | [[39](#_ENREF_39)] |
| miR-218(rs11134527) | Breast cancer | AG vs. AA | Lianghe Jiao | 2014 | Chinese | 196 | OS | 1.46 | 3.22 | 0.96 | [[10](#_ENREF_10)] |
| miR-218(rs11134527) | Breast cancer | GG vs. AA | Lianghe Jiao | 2014 | Chinese | 196 | OS | 1.19 | 2.14 | 0.96 | [[10](#_ENREF_10)] |
| miR-218(rs11134527) | Breast cancer | AG+GG vs. AA | Lianghe Jiao | 2014 | Chinese | 196 | OS | 1.32 | 2.70 | 0.96 | [[10](#_ENREF_10)] |
| miR-218(rs11134527) | Breast cancer | GG vs. AG+AA | Lianghe Jiao | 2014 | Chinese | 196 | OS | 1.25 | 2.17 | 0.96 | [[10](#_ENREF_10)] |
| miR-218(rs11134527) | ESCC | AG vs. AA | Lin Jiang | 2014 | Chinese | 706 | OS | 0.99 | 1.24 | 0.96 | [[40](#_ENREF_40)] |
| miR-218(rs11134527) | ESCC | GG vs. AA | Lin Jiang | 2014 | Chinese | 706 | OS | 0.73 | 1.06 | 0.96 | [[40](#_ENREF_40)] |
| miR-218(rs11134527) | ESCC | GG vs. AG+AA | Lin Jiang | 2014 | Chinese | 706 | OS | 0.73 | 1.04 | 0.96 | [[40](#_ENREF_40)] |
| miR-218-1(rs3775815) | NSCLC | CA vs. CC | Shuangshuang Wu | 2015 | Chinese | 1001 | OS | 0.79 | 0.94 | 0.96 | [[9](#_ENREF_9)] |
| miR-218-1(rs3775815) | NSCLC | AA vs. CC | Shuangshuang Wu | 2015 | Chinese | 1001 | OS | 0.59 | 0.92 | 0.96 | [[9](#_ENREF_9)] |
| miR-218-1(rs3775815) | NSCLC | CA+AA vs. CC | Shuangshuang Wu | 2015 | Chinese | 1001 | OS | 0.76 | 0.91 | 0.96 | [[9](#_ENREF_9)] |
| miR-218-2(rs4867902) | NSCLC | AG vs. AA | Shuangshuang Wu | 2015 | Chinese | 1001 | OS | 1.24 | 1.49 | 0.96 | [[9](#_ENREF_9)] |
| miR-218-2(rs4867902) | NSCLC | GG vs. AA | Shuangshuang Wu | 2015 | Chinese | 1001 | OS | 1.53 | 2.01 | 0.96 | [[9](#_ENREF_9)] |
| miR-218-2(rs4867902) | NSCLC | AG+GG vs. AA | Shuangshuang Wu | 2015 | Chinese | 1001 | OS | 1.30 | 1.54 | 0.96 | [[9](#_ENREF_9)] |
| miR-219-1(rs107822) | NSCLC | GA vs. AA | Chang Zheng | 2017 | Chinese | 405 | OS | 1.11 | 1.53 | 0.96 | [[41](#_ENREF_41)] |
| miR-219-1(rs107822) | NSCLC | GG vs. AA | Chang Zheng | 2017 | Chinese | 405 | OS | 1.38 | 1.96 | 0.96 | [[41](#_ENREF_41)] |
| miR-219-1(rs213210) | GC | CC+CT vs. TT | Yanhua Wu | 2017 | Chinese | 735 | OS | 1.01 | 1.32 | 0.96 | [[39](#_ENREF_39)] |
| miR-219-1(rs213210) | NSCLC | AG vs. AA | Kyong-Ah Yoon | 2012 | Korean | 388 | RFS | 1.16 | 2.08 | 0.96 | [[13](#_ENREF_13)] |
| miR-219-1(rs213210) | NSCLC | GG vs. AA | Kyong-Ah Yoon | 2012 | Korean | 388 | RFS | 1.18 | 2.31 | 0.96 | [[13](#_ENREF_13)] |
| miR-219-1(rs213210) | NSCLC | AG+GG vs. AA | Kyong-Ah Yoon | 2012 | Korean | 388 | RFS | 1.17 | 2.03 | 0.96 | [[13](#_ENREF_13)] |
| miR-219-1(rs213210) | NSCLC | CT vs. TT | Chang Zheng | 2017 | Chinese | 405 | OS | 1.34 | 1.84 | 0.96 | [[41](#_ENREF_41)] |
| miR-219-1(rs213210) | NSCLC | CC vs. TT | Chang Zheng | 2017 | Chinese | 405 | OS | 1.33 | 1.91 | 0.96 | [[41](#_ENREF_41)] |
| miR-219-1(rs421446) | NSCLC | TC vs. CC | Chang Zheng | 2017 | Chinese | 405 | OS | 0.83 | 1.09 | 0.96 | [[41](#_ENREF_41)] |
| miR-219-1(rs421446) | NSCLC | TT vs. CC | Chang Zheng | 2017 | Chinese | 405 | OS | 0.88 | 1.26 | 0.96 | [[41](#_ENREF_41)] |
| mir-26a-1(rs7372209) | ESCC | CT vs. CC | Pei-Wen Yang | 2014 | Chinese | 504 | OS | 1.13 | 1.41 | 0.96 | [[34](#_ENREF_34)] |
| mir-26a-1(rs7372209) | ESCC | TT vs. CC | Pei-Wen Yang | 2014 | Chinese | 504 | OS | 1.04 | 1.51 | 0.96 | [[34](#_ENREF_34)] |
| mir-26a-1(rs7372209) | ESCC | CT vs. CC | Pei-Wen Yang | 2014 | Chinese | 504 | RFS | 1.17 | 1.45 | 0.96 | [[34](#_ENREF_34)] |
| mir-26a-1(rs7372209) | ESCC | TT vs. CC | Pei-Wen Yang | 2014 | Chinese | 504 | RFS | 1.06 | 1.51 | 0.96 | [[34](#_ENREF_34)] |
| mir-26a-1(rs7372209) | NSCLC | CT vs. CC | Kyong-Ah Yoon | 2012 | Korean | 388 | RFS | 1.16 | 1.91 | 0.96 | [[13](#_ENREF_13)] |
| mir-26a-1(rs7372209) | NSCLC | TT vs. CC | Kyong-Ah Yoon | 2012 | Korean | 388 | RFS | 1.14 | 2.62 | 0.96 | [[13](#_ENREF_13)] |
| mir-26a-1(rs7372209) | NSCLC | CT+TT vs. CC | Kyong-Ah Yoon | 2012 | Korean | 388 | RFS | 1.16 | 1.86 | 0.96 | [[13](#_ENREF_13)] |
| miR-27a(rs895819) | Breast cancer | CT+CC vs. TT | Ning Zhan | 2013 | Chinese | 62 | OS | 0.55 | 8.44 | 0.96 | [[42](#_ENREF_42)] |
| miR-27a(rs895819) | Breast cancer | CT+CC vs. TT | Ning Zhan | 2013 | Chinese | 62 | RFS | 0.59 | 5.22 | 0.96 | [[42](#_ENREF_42)] |
| miR-27a(rs895819) | NSCLC | CT vs. TT | Kyong-Ah Yoon | 2012 | Korean | 388 | RFS | 1.03 | 1.72 | 0.96 | [[13](#_ENREF_13)] |
| miR-27a(rs895819) | NSCLC | CC vs. TT | Kyong-Ah Yoon | 2012 | Korean | 388 | RFS | 0.81 | 1.85 | 0.96 | [[13](#_ENREF_13)] |
| miR-27a(rs895819) | NSCLC | CT+CC vs. TT | Kyong-Ah Yoon | 2012 | Korean | 388 | RFS | 0.98 | 1.60 | 0.96 | [[13](#_ENREF_13)] |
| miR-27a(rs895819) | NSCLC | CT vs. TT | Ji-Yong Ma | 2015 | Chinese | 542 | OS | 1.24 | 1.61 | 0.96 | [[43](#_ENREF_43)] |
| miR-27a(rs895819) | NSCLC | CC vs. TT | Ji-Yong Ma | 2015 | Chinese | 542 | OS | 1.22 | 1.63 | 0.96 | [[43](#_ENREF_43)] |
| miR-27a(rs895819) | NSCLC | CT+CC vs. TT | Ji-Yong Ma | 2015 | Chinese | 542 | OS | 1.25 | 1.56 | 0.96 | [[43](#_ENREF_43)] |
| miR-27a(rs895819) | NSCLC | CC vs. CT+TT | Ji-Yong Ma | 2015 | Chinese | 542 | OS | 1.13 | 1.47 | 0.96 | [[43](#_ENREF_43)] |
| miR-27a(rs895819) | NSCLC | CT vs. TT | Ji-Yong Ma | 2015 | Chinese | 542 | RFS | 1.11 | 1.42 | 0.96 | [[43](#_ENREF_43)] |
| miR-27a(rs895819) | NSCLC | CC vs. TT | Ji-Yong Ma | 2015 | Chinese | 542 | RFS | 0.95 | 1.25 | 0.96 | [[43](#_ENREF_43)] |
| miR-27a(rs895819) | NSCLC | CT+CC vs. TT | Ji-Yong Ma | 2015 | Chinese | 542 | RFS | 1.08 | 1.33 | 0.96 | [[43](#_ENREF_43)] |
| miR-27a(rs895819) | NSCLC | CC vs. CT+TT | Ji-Yong Ma | 2015 | Chinese | 542 | RFS | 0.90 | 1.16 | 0.96 | [[43](#_ENREF_43)] |
| miR-27a(rs895819) | NSCLC | CT+CC vs. TT | Jiali Xu | 2013 | Chinese | 576 | OS | 1.71 | 2.26 | 0.96 | [[44](#_ENREF_44)] |
| miR-27a(rs895819) | NSCLC | CT vs. TT | Jiali Xu | 2013 | Chinese | 576 | OS | 1.72 | 2.31 | 0.96 | [[44](#_ENREF_44)] |
| miR-27a(rs895819) | NSCLC | CC vs. TT | Jiali Xu | 2013 | Chinese | 576 | OS | 1.66 | 2.80 | 0.96 | [[44](#_ENREF_44)] |
| miR-27a(rs895819) | CRC | CT vs. TT | Jinliang Xing | 2012 | Chinese | 408 | OS | 0.88 | 1.41 | 0.96 | [[15](#_ENREF_15)] |
| miR-27a(rs895819) | CRC | CC vs. TT | Jinliang Xing | 2012 | Chinese | 408 | OS | 0.66 | 1.70 | 0.96 | [[15](#_ENREF_15)] |
| miR-27a(rs895819) | CRC | TC+CC vs. TT | Jinliang Xing | 2012 | Chinese | 408 | OS | 0.85 | 1.34 | 0.96 | [[15](#_ENREF_15)] |
| miR-27a(rs895819) | CRC | CC vs. TC+TT | Jinliang Xing | 2012 | Chinese | 408 | OS | 0.71 | 1.76 | 0.96 | [[15](#_ENREF_15)] |
| miR-27a(rs895819) | CRC | CT vs. TT | Jinliang Xing | 2012 | Chinese | 408 | RFS | 0.75 | 1.13 | 0.96 | [[15](#_ENREF_15)] |
| miR-27a(rs895819) | CRC | CC vs. TT | Jinliang Xing | 2012 | Chinese | 408 | RFS | 0.76 | 1.64 | 0.96 | [[15](#_ENREF_15)] |
| miR-27a(rs895819) | CRC | TC+CC vs. TT | Jinliang Xing | 2012 | Chinese | 408 | RFS | 0.75 | 1.11 | 0.96 | [[15](#_ENREF_15)] |
| miR-27a(rs895819) | CRC | CC vs. TC+TT | Jinliang Xing | 2012 | Chinese | 408 | RFS | 0.87 | 1.85 | 0.96 | [[15](#_ENREF_15)] |
| miR-29c(rs2724377) | NSCLC | AG vs. AA | Zhibin Hu (screening) | 2011 | Chinese | 568 | OS | 1.01 | 1.29 | 0.96 | [[5](#_ENREF_5)] |
| miR-29c(rs2724377) | NSCLC | GG vs. AA | Zhibin Hu (screening) | 2011 | Chinese | 568 | OS | 1.66 | 2.73 | 0.96 | [[5](#_ENREF_5)] |
| miR-29c(rs2724377) | NSCLC | GG vs. AA+AG | Zhibin Hu (screening) | 2011 | Chinese | 568 | OS | 1.65 | 2.70 | 0.96 | [[5](#_ENREF_5)] |
| miR-29c(rs2724377) | NSCLC | AG vs. AA | Zhibin Hu (validation) | 2011 | Chinese | 355 | OS | 0.74 | 1.05 | 0.96 | [[5](#_ENREF_5)] |
| miR-29c(rs2724377) | NSCLC | GG vs. AA | Zhibin Hu (validation) | 2011 | Chinese | 355 | OS | 1.21 | 1.34 | 0.96 | [[5](#_ENREF_5)] |
| miR-29c(rs2724377) | NSCLC | GG vs. AA+AG | Zhibin Hu (validation) | 2011 | Chinese | 355 | OS | 1.35 | 3.68 | 0.96 | [[5](#_ENREF_5)] |
| miR-30c (rs928508) | GC | AG+GG vs. AA | Yong-ping Mu | 2012 | Chinese | 92 | OS | 0.51 | 0.98 | 0.96 | [[45](#_ENREF_45)] |
| mir-30c-1(rs16827546) | ESCC | CT vs. CC | Pei-Wen Yang | 2014 | Chinese | 129 | RFS | 0.96 | 1.76 | 0.96 | [[34](#_ENREF_34)] |
| miR-30c-1(rs928508) | NSCLC | AG vs. AA | Zhibin Hu (screening) | 2011 | Chinese | 568 | OS | 0.81 | 1.05 | 0.96 | [[5](#_ENREF_5)] |
| miR-30c-1(rs928508) | NSCLC | GG vs. AA | Zhibin Hu (screening) | 2011 | Chinese | 568 | OS | 0.59 | 0.82 | 0.96 | [[5](#_ENREF_5)] |
| miR-30c-1(rs928508) | NSCLC | AG vs. AA | Zhibin Hu (validation) | 2011 | Chinese | 355 | OS | 0.64 | 0.92 | 0.96 | [[5](#_ENREF_5)] |
| miR-30c-1(rs928508) | NSCLC | GG vs. AA | Zhibin Hu (validation) | 2011 | Chinese | 355 | OS | 0.82 | 1.26 | 0.96 | [[5](#_ENREF_5)] |
| miR-30c-1(rs928508) | NSCLC | AG vs. AA | Zhibin Hu | 2011 | Chinese | 923 | OS | 0.76 | 0.93 | 0.96 | [[5](#_ENREF_5)] |
| miR-30c-1(rs928508) | NSCLC | GG vs. AA | Zhibin Hu | 2011 | Chinese | 923 | OS | 0.69 | 0.89 | 0.96 | [[5](#_ENREF_5)] |
| miR-30c-1(rs928508) | NSCLC | AG+GG vs. AA | Zhibin Hu | 2011 | Chinese | 923 | OS | 0.73 | 0.89 | 0.96 | [[5](#_ENREF_5)] |
| miR-31(rs13283671) | NSCLC | AG vs. AA | Zhibin Hu (screening) | 2011 | Chinese | 568 | OS | 1.01 | 1.28 | 0.96 | [[5](#_ENREF_5)] |
| miR-31(rs13283671) | NSCLC | GG vs. AA | Zhibin Hu (screening) | 2011 | Chinese | 568 | OS | 0.53 | 0.91 | 0.96 | [[5](#_ENREF_5)] |
| miR-31(rs13283671) | NSCLC | GG vs. AA+AG | Zhibin Hu (screening) | 2011 | Chinese | 568 | OS | 0.53 | 0.90 | 0.96 | [[5](#_ENREF_5)] |
| miR-31(rs13283671) | NSCLC | AG vs. AA | Zhibin Hu (validation) | 2011 | Chinese | 355 | OS | 0.96 | 1.34 | 0.96 | [[5](#_ENREF_5)] |
| miR-31(rs13283671) | NSCLC | GG vs. AA | Zhibin Hu (validation) | 2011 | Chinese | 355 | OS | 1.79 | 3.18 | 0.96 | [[5](#_ENREF_5)] |
| miR-31(rs13283671) | NSCLC | GG vs. AA+AG | Zhibin Hu (validation) | 2011 | Chinese | 355 | OS | 1.82 | 1.82 | 0.96 | [[5](#_ENREF_5)] |
| miR-33(rs9620000) | NSCLC | AG vs. AA | Zhibin Hu (screening) | 2011 | Chinese | 568 | OS | 1.10 | 1.49 | 0.96 | [[5](#_ENREF_5)] |
| miR-33(rs9620000) | NSCLC | GG vs. AA | Zhibin Hu (screening) | 2011 | Chinese | 568 | OS | 6.60 | 20.90 | 0.96 | [[5](#_ENREF_5)] |
| miR-33(rs9620000) | NSCLC | AG vs. AA | Zhibin Hu (validation) | 2011 | Chinese | 355 | OS | 1.23 | 1.82 | 0.96 | [[5](#_ENREF_5)] |
| miR-34 (rs4938723) | HCC | TC vs. TT | Myung Su Son | 2013 | Korean | 157 | OS | 0.93 | 1.40 | 0.96 | [[46](#_ENREF_46)] |
| miR-34 (rs4938723) | HCC | CC vs. TT | Myung Su Son | 2013 | Korean | 157 | OS | 0.73 | 1.50 | 0.96 | [[46](#_ENREF_46)] |
| miR-34 (rs4938723) | HCC | TT vs. TC+CC | Myung Su Son | 2013 | Korean | 157 | OS | 0.88 | 1.30 | 0.96 | [[46](#_ENREF_46)] |
| miR-34 (rs4938723) | HCC | TT+TC vs. CC | Myung Su Son | 2013 | Korean | 157 | OS | 0.78 | 1.52 | 0.96 | [[46](#_ENREF_46)] |
| miR-34b/c(rs4938723) | GC | CC+CT vs. TT | Yanhua Wu | 2017 | Chinese | 735 | OS | 0.92 | 1.14 | 0.96 | [[39](#_ENREF_39)] |
| miR-34b/c(rs4938723) | Breast cancer | CC vs. TC+TT | Jeannette T. Bensen | 2013 | American | 1946 | OS | 0.65 | 1.02 | 0.96 | [[29](#_ENREF_29)] |
| miR-367(rs13136737) | NSCLC | CA vs. CC | Zhibin Hu (screening) | 2011 | Chinese | 568 | OS | 1.27 | 1.62 | 0.96 | [[5](#_ENREF_5)] |
| miR-367(rs13136737) | NSCLC | AA vs. CC | Zhibin Hu (screening) | 2011 | Chinese | 568 | OS | 1.64 | 2.32 | 0.96 | [[5](#_ENREF_5)] |
| miR-367(rs13136737) | NSCLC | CA+AA vs. CC | Zhibin Hu (screening) | 2011 | Chinese | 568 | OS | 1.34 | 1.69 | 0.96 | [[5](#_ENREF_5)] |
| miR-367(rs13136737) | NSCLC | CA vs. CC | Zhibin Hu (validation) | 2011 | Chinese | 355 | OS | 0.95 | 1.31 | 0.96 | [[5](#_ENREF_5)] |
| miR-367(rs13136737) | NSCLC | AA vs. CC | Zhibin Hu (validation) | 2011 | Chinese | 355 | OS | 0.82 | 1.37 | 0.96 | [[5](#_ENREF_5)] |
| miR-367(rs13136737) | NSCLC | CA+AA vs. CC | Zhibin Hu (validation) | 2011 | Chinese | 355 | OS | 0.92 | 1.25 | 0.96 | [[5](#_ENREF_5)] |
| miR-378(rs1076064) | NSCLC | AG vs. AA | Zhibin Hu (screening) | 2011 | Chinese | 568 | OS | 0.77 | 1.00 | 0.96 | [[5](#_ENREF_5)] |
| miR-378(rs1076064) | NSCLC | GG vs. AA | Zhibin Hu (screening) | 2011 | Chinese | 568 | OS | 0.67 | 0.91 | 0.96 | [[5](#_ENREF_5)] |
| miR-378(rs1076064) | NSCLC | AG+GG vs. AA | Zhibin Hu (screening) | 2011 | Chinese | 568 | OS | 0.73 | 0.94 | 0.96 | [[5](#_ENREF_5)] |
| miR-378(rs1076064) | NSCLC | AG vs. AA | Zhibin Hu (validation) | 2011 | Chinese | 355 | OS | 1.05 | 1.54 | 0.96 | [[5](#_ENREF_5)] |
| miR-378(rs1076064) | NSCLC | GG vs. AA | Zhibin Hu (validation) | 2011 | Chinese | 355 | OS | 1.22 | 1.91 | 0.96 | [[5](#_ENREF_5)] |
| miR-378(rs1076064) | NSCLC | AG+GG vs. AA | Zhibin Hu (validation) | 2011 | Chinese | 355 | OS | 1.10 | 1.58 | 0.96 | [[5](#_ENREF_5)] |
| miR-378(rs1076064) | HCC | AG vs. AA | Jiaze An | 2014 | Chinese | 331 | OS | 0.78 | 1.05 | 0.96 | [[47](#_ENREF_47)] |
| miR-378(rs1076064) | HCC | GG vs. AA | Jiaze An | 2014 | Chinese | 331 | OS | 0.48 | 0.96 | 0.69 | [[47](#_ENREF_47)] |
| miR-379(rs61991156) | GC | AA vs. AG+GG | Na Cao | 2018 | Chinese | 217 | OS | 2.02 | 2.72 | 1.38 | [[48](#_ENREF_48)] |
| mir-423 (rs6505162) | ESCC | CA vs. CC | Pei-Wen Yang | 2014 | Chinese | 129 | OS | 1.33 | 2.20 | 0.96 | [[34](#_ENREF_34)] |
| mir-423 (rs6505162) | ESCC | AA vs. CC | Pei-Wen Yang | 2014 | Chinese | 129 | OS | 1.86 | 13.92 | 0.96 | [[34](#_ENREF_34)] |
| mir-423 (rs6505162) | ESCC | CA vs. CC | Pei-Wen Yang | 2014 | Chinese | 129 | RFS | 1.09 | 1.69 | 0.96 | [[34](#_ENREF_34)] |
| mir-423 (rs6505162) | ESCC | AA vs. CC | Pei-Wen Yang | 2014 | Chinese | 129 | RFS | 0.87 | 6.40 | 0.96 | [[34](#_ENREF_34)] |
| mir-423 (rs6505162) | CRC | CA vs. CC | Jinliang Xing | 2012 | Chinese | 408 | OS | 2.18 | 3.51 | 0.96 | [[15](#_ENREF_15)] |
| mir-423 (rs6505162) | CRC | AA vs. CC | Jinliang Xing | 2012 | Chinese | 408 | OS | 1.79 | 4.28 | 0.96 | [[15](#_ENREF_15)] |
| mir-423 (rs6505162) | CRC | CA+AA vs. CC | Jinliang Xing | 2012 | Chinese | 408 | OS | 2.12 | 3.34 | 0.96 | [[15](#_ENREF_15)] |
| mir-423 (rs6505162) | CRC | AA vs. CA+CC | Jinliang Xing | 2012 | Chinese | 408 | OS | 1.29 | 2.99 | 0.96 | [[15](#_ENREF_15)] |
| mir-423 (rs6505162) | CRC | CA vs. CC | Jinliang Xing | 2012 | Chinese | 408 | RFS | 1.73 | 2.59 | 0.96 | [[15](#_ENREF_15)] |
| mir-423 (rs6505162) | CRC | AA vs. CC | Jinliang Xing | 2012 | Chinese | 408 | RFS | 1.02 | 2.36 | 0.96 | [[15](#_ENREF_15)] |
| mir-423 (rs6505162) | CRC | CA+AA vs. CC | Jinliang Xing | 2012 | Chinese | 408 | RFS | 1.59 | 2.36 | 0.96 | [[15](#_ENREF_15)] |
| mir-423 (rs6505162) | CRC | AA vs. CA+CC | Jinliang Xing | 2012 | Chinese | 408 | RFS | 0.82 | 1.87 | 0.96 | [[15](#_ENREF_15)] |
| mir-423 (rs6505162) | NSCLC | AC vs. CC | Kyong-Ah Yoon | 2012 | Korean | 388 | RFS | 1.18 | 1.95 | 0.96 | [[13](#_ENREF_13)] |
| mir-423 (rs6505162) | NSCLC | AA vs. CC | Kyong-Ah Yoon | 2012 | Korean | 388 | RFS | 0.69 | 2.24 | 0.96 | [[13](#_ENREF_13)] |
| mir-423 (rs6505162) | NSCLC | AC+AA vs. CC | Kyong-Ah Yoon | 2012 | Korean | 388 | RFS | 1.09 | 1.77 | 0.96 | [[13](#_ENREF_13)] |
| miR-423(rs6505162) | Breast cancer | AC vs. CC | Lianghe Jiao | 2014 | Chinese | 196 | OS | 0.64 | 2.64 | 0.96 | [[10](#_ENREF_10)] |
| miR-423(rs6505162) | Breast cancer | AA vs. CC | Lianghe Jiao | 2014 | Chinese | 196 | OS | 0.66 | 1.22 | 0.96 | [[10](#_ENREF_10)] |
| miR-423(rs6505162) | Breast cancer | AC+AA vs. CC | Lianghe Jiao | 2014 | Chinese | 196 | OS | 0.66 | 1.18 | 0.96 | [[10](#_ENREF_10)] |
| miR-423(rs6505162) | Breast cancer | AA vs. AC+CC | Lianghe Jiao | 2014 | Chinese | 196 | OS | 0.72 | 2.97 | 0.96 | [[10](#_ENREF_10)] |
| miR-423(rs6505162) | NSCLC | AC vs. CC | Xia Lingzi | 2016 | Chinese | 584 | OS | 1.02 | 1.28 | 0.96 | [[19](#_ENREF_19)] |
| miR-423(rs6505162) | NSCLC | AA vs. CC | Xia Lingzi | 2016 | Chinese | 584 | OS | 0.95 | 1.80 | 0.96 | [[19](#_ENREF_19)] |
| miR-423(rs6505162) | SCCOP | TT vs. CT+CC | Xingming Chen | 2016 | Chinese | 1008 | DFS | 1.10 | 1.80 | 0.96 | [[12](#_ENREF_12)] |
| miR-423(rs6505162) | ESCC | CA vs. CC | Meenakshi Umar | 2013 | India | 153 | OS | 1.06 | 1.69 | 0.96 | [[26](#_ENREF_26)] |
| miR-423(rs6505162) | ESCC | AA vs. CC | Meenakshi Umar | 2013 | India | 153 | OS | 1.10 | 1.93 | 0.96 | [[26](#_ENREF_26)] |
| mir-4302(rs11048315) | NSCLC | AG vs. GG | Yang Zhao | 2014 | American | 452 | OS | 0.59 | 0.84 | 0.96 | [[30](#_ENREF_30)] |
| mir-4302(rs11048315) | NSCLC | AA vs. GG | Yang Zhao | 2014 | American | 452 | OS | 0.88 | 2.80 | 0.96 | [[30](#_ENREF_30)] |
| mir-4422(rs17111728) | NSCLC | TC vs. TT | Yang Zhao | 2014 | American | 526 | OS | 0.63 | 0.86 | 0.96 | [[30](#_ENREF_30)] |
| mir-4422(rs17111728) | NSCLC | CC vs. TT | Yang Zhao | 2014 | American | 526 | OS | 0.25 | 1.01 | 0.96 | [[30](#_ENREF_30)] |
| mir-4741(rs7227168) | NSCLC | TC vs. CC | Yang Zhao | 2014 | American | 526 | OS | 1.49 | 1.91 | 0.96 | [[30](#_ENREF_30)] |
| mir-4741(rs7227168) | NSCLC | TT vs. CC | Yang Zhao | 2014 | American | 526 | OS | 1.31 | 2.60 | 0.96 | [[30](#_ENREF_30)] |
| mir-4742(rs7522956 ) | NSCLC | AC vs. AA | Yang Zhao | 2014 | American | 452 | OS | 1.30 | 1.73 | 0.96 | [[30](#_ENREF_30)] |
| mir-4742(rs7522956 ) | NSCLC | CC vs. AA | Yang Zhao | 2014 | American | 452 | OS | 2.20 | 3.77 | 0.96 | [[30](#_ENREF_30)] |
| miR-492(rs2289030) | CRC | GC vs. CC | Jinliang Xing | 2012 | Chinese | 408 | OS | 0.72 | 1.18 | 0.96 | [[15](#_ENREF_15)] |
| miR-492(rs2289030) | CRC | GG vs. CC | Jinliang Xing | 2012 | Chinese | 408 | OS | 1.05 | 2.40 | 0.96 | [[15](#_ENREF_15)] |
| miR-492(rs2289030) | CRC | GC+GG vs. CC | Jinliang Xing | 2012 | Chinese | 408 | OS | 0.77 | 1.23 | 0.96 | [[15](#_ENREF_15)] |
| miR-492(rs2289030) | CRC | GG vs. GC+CC | Jinliang Xing | 2012 | Chinese | 408 | OS | 1.19 | 2.68 | 0.96 | [[15](#_ENREF_15)] |
| miR-492(rs2289030) | CRC | GC vs. CC | Jinliang Xing | 2012 | Chinese | 408 | RFS | 0.83 | 1.26 | 0.96 | [[15](#_ENREF_15)] |
| miR-492(rs2289030) | CRC | GG vs. CC | Jinliang Xing | 2012 | Chinese | 408 | RFS | 0.95 | 2.03 | 0.96 | [[15](#_ENREF_15)] |
| miR-492(rs2289030) | CRC | GC+GG vs. CC | Jinliang Xing | 2012 | Chinese | 408 | RFS | 0.85 | 1.26 | 0.96 | [[15](#_ENREF_15)] |
| miR-492(rs2289030) | CRC | GG vs. GC+CC | Jinliang Xing | 2012 | Chinese | 408 | RFS | 1.02 | 2.15 | 0.96 | [[15](#_ENREF_15)] |
| miR-492(rs2289030) | NSCLC | CG vs. GG | Kyong-Ah Yoon | 2012 | Korean | 388 | RFS | 1.12 | 1.83 | 0.96 | [[13](#_ENREF_13)] |
| miR-492(rs2289030) | NSCLC | GG vs. CC | Kyong-Ah Yoon | 2012 | Korean | 388 | RFS | 1.79 | 7.14 | 0.96 | [[13](#_ENREF_13)] |
| miR-492(rs2289030) | NSCLC | GG vs. GC+CC | Kyong-Ah Yoon | 2012 | Korean | 388 | RFS | 0.96 | 1..54 | 0.96 | [[13](#_ENREF_13)] |
| miR-492(rs2289030) | SCCOP | CC vs. CG+GG | Xingming Chen | 2016 | Chinese | 1008 | DFS | 0.90 | 1.30 | 0.96 | [[12](#_ENREF_12)] |
| miR-492(rs2289030) | HCC | CG vs. CC | Guopeng Yu | 2016 | Chinese | 362 | OS | 0.73 | 0.97 | 0.96 | [[49](#_ENREF_49)] |
| miR-492(rs2289030) | HCC | GG vs. CC | Guopeng Yu | 2016 | Chinese | 362 | OS | 0.85 | 1.19 | 0.96 | [[49](#_ENREF_49)] |
| miR-492(rs2289030) | HCC | GG vs. CG+CC | Guopeng Yu | 2016 | Chinese | 362 | OS | 0.90 | 1.26 | 0.96 | [[49](#_ENREF_49)] |
| miR-492(rs2289030) | HCC | CG+GG vs. CC | Guopeng Yu | 2016 | Chinese | 362 | OS | 0.73 | 0.96 | 0.96 | [[49](#_ENREF_49)] |
| miR-499(rs3746444) | NSCLC | CT vs. TT | Mi Jeong Hong | 2013 | Korean | 363 | OS | 1.02 | 1.49 | 0.96 | [[16](#_ENREF_16)] |
| miR-499(rs3746444) | NSCLC | CC vs. TT | Mi Jeong Hong | 2013 | Korean | 363 | OS | 0.61 | 1.68 | 0.96 | [[16](#_ENREF_16)] |
| miR-499(rs3746444) | NSCLC | CC vs. CT+TT | Mi Jeong Hong | 2013 | Korean | 363 | OS | 0.61 | 1.66 | 0.96 | [[16](#_ENREF_16)] |
| miR-499(rs3746444) | NSCLC | CT vs. TT | Mi Jeong Hong | 2013 | Korean | 363 | DFS | 0.98 | 1.38 | 0.96 | [[16](#_ENREF_16)] |
| miR-499(rs3746444) | NSCLC | CC vs. TT | Mi Jeong Hong | 2013 | Korean | 363 | DFS | 0.76 | 1.75 | 0.96 | [[16](#_ENREF_16)] |
| miR-499(rs3746444) | NSCLC | CC vs. CT+TT | Mi Jeong Hong | 2013 | Korean | 363 | DFS | 0.77 | 1.75 | 0.96 | [[16](#_ENREF_16)] |
| mir-499(rs3746444) | NSCLC | CT vs. TT | Zhibin Hu | 2008 | Chinese | 663 | OS | 1.11 | 1.46 | 0.96 | [[17](#_ENREF_17)] |
| mir-499(rs3746444) | NSCLC | CC vs. TT | Zhibin Hu | 2008 | Chinese | 663 | OS | 1.24 | 2.83 | 0.96 | [[17](#_ENREF_17)] |
| mir-499(rs3746444) | SCCNOP | TT vs. CT+CC | Chengyuan Wang | 2016 | Chinese | 996 | OS | 0.60 | 0.90 | 0.96 | [[18](#_ENREF_18)] |
| mir-499(rs3746444) | SCCNOP | TT vs. CT+CC | Chengyuan Wang | 2016 | Chinese | 996 | DFS | 0.50 | 0.70 | 0.96 | [[18](#_ENREF_18)] |
| mir-499(rs3746444) | SCCOP | TT vs. CT+CC | Xiaoxiang Guan | 2013 | Chinese | 281 | OS | 1.00 | 2.00 | 0.96 | [[20](#_ENREF_20)] |
| mir-499(rs3746444) | SCCOP | TT vs. CT+CC | Xiaoxiang Guan | 2013 | Chinese | 281 | DFS | 0.80 | 1.90 | 0.96 | [[20](#_ENREF_20)] |
| miR-499(rs3746444) | SCCOP | TT vs. CT+CC | Xingming Chen | 2016 | Chinese | 1008 | DFS | 1.40 | 1.70 | 0.96 | [[12](#_ENREF_12)] |
| miR-499(rs3746444) | GC | CT vs. TT | Dae Ho Ahn | 2013 | Korean | 160 | OS | 1.50 | 3.20 | 0.96 | [[24](#_ENREF_24)] |
| miR-499(rs3746444) | GC | CC vs. TT | Dae Ho Ahn | 2013 | Korean | 160 | OS | 1.60 | 7.40 | 0.96 | [[24](#_ENREF_24)] |
| miR-499(rs3746444) | GC | CT+CC vs. TT | Dae Ho Ahn | 2013 | Korean | 160 | OS | 1.50 | 3.10 | 0.96 | [[24](#_ENREF_24)] |
| miR-499(rs3746444) | HCC | CT vs. TT | Won Hee Kim | 2012 | Korean | 67 | OS | 0.65 | 1.18 | 0.96 | [[25](#_ENREF_25)] |
| miR-499(rs3746444) | ESCC | CT vs. TT | Meenakshi Umar | 2013 | India | 153 | OS | 0.89 | 1.35 | 0.96 | [[26](#_ENREF_26)] |
| miR-499(rs3746444) | ESCC | CC vs. TT | Meenakshi Umar | 2013 | India | 153 | OS | 0.83 | 1.87 | 0.96 | [[26](#_ENREF_26)] |
| mir-499a(rs3746444) | CRC | CT vs. TT | MOON JU JANG | 2011 | Korean | 407 | OS | 1.02 | 1.62 | 0.96 | [[14](#_ENREF_14)] |
| mir-499a(rs3746444) | CRC | CC vs. TT | MOON JU JANG | 2011 | Korean | 407 | OS | 1.14 | 4.68 | 0.96 | [[14](#_ENREF_14)] |
| mir-499a(rs3746444) | CRC | CT+CC vs. TT | MOON JU JANG | 2011 | Korean | 407 | OS | 1.03 | 1.62 | 0.96 | [[14](#_ENREF_14)] |
| mir-499a(rs3746444) | CRC | CC vs. CT+TT | MOON JU JANG | 2011 | Korean | 407 | OS | 1.13 | 4.60 | 0.96 | [[14](#_ENREF_14)] |
| mir-499a(rs3746444) | CRC | CT vs. TT | MOON JU JANG | 2011 | Korean | 407 | RFS | 0.94 | 1.53 | 0.96 | [[14](#_ENREF_14)] |
| mir-499a(rs3746444) | CRC | CC vs. TT | MOON JU JANG | 2011 | Korean | 407 | RFS | 1.71 | 5.56 | 0.96 | [[14](#_ENREF_14)] |
| mir-499a(rs3746444) | CRC | CT+CC vs. TT | MOON JU JANG | 2011 | Korean | 407 | RFS | 0.98 | 1.58 | 0.96 | [[14](#_ENREF_14)] |
| mir-499a(rs3746444) | CRC | CC vs. CT+TT | MOON JU JANG | 2011 | Korean | 407 | RFS | 1.75 | 5.62 | 0.96 | [[14](#_ENREF_14)] |
| mir-5197(rs2042253) | NSCLC | TC vs. TT | Yang Zhao | 2014 | American | 526 | OS | 0.79 | 0.97 | 0.96 | [[30](#_ENREF_30)] |
| mir-5197(rs2042253) | NSCLC | CC vs. TT | Yang Zhao | 2014 | American | 526 | OS | 0.65 | 1.01 | 0.96 | [[30](#_ENREF_30)] |
| miR-604(rs2368392) | CRC | TC vs. CC | Jinliang Xing | 2012 | Chinese | 408 | OS | 1.17 | 1.88 | 0.96 | [[15](#_ENREF_15)] |
| miR-604(rs2368392) | CRC | TT vs. CC | Jinliang Xing | 2012 | Chinese | 408 | OS | 1.46 | 3.11 | 0.96 | [[15](#_ENREF_15)] |
| miR-604(rs2368392) | CRC | TC+TT vs. CC | Jinliang Xing | 2012 | Chinese | 408 | OS | 1.22 | 1.91 | 0.96 | [[15](#_ENREF_15)] |
| miR-604(rs2368392) | CRC | TT vs. TC+CC | Jinliang Xing | 2012 | Chinese | 408 | OS | 1.37 | 2.84 | 0.96 | [[15](#_ENREF_15)] |
| miR-604(rs2368392) | CRC | TC vs. CC | Jinliang Xing | 2012 | Chinese | 408 | RFS | 0.97 | 1.47 | 0.96 | [[15](#_ENREF_15)] |
| miR-604(rs2368392) | CRC | TT vs. CC | Jinliang Xing | 2012 | Chinese | 408 | RFS | 1.47 | 2.77 | 0.96 | [[15](#_ENREF_15)] |
| miR-604(rs2368392) | CRC | TC+TT vs. CC | Jinliang Xing | 2012 | Chinese | 408 | RFS | 1.06 | 1.55 | 0.96 | [[15](#_ENREF_15)] |
| miR-604(rs2368392) | CRC | TT vs. TC+CC | Jinliang Xing | 2012 | Chinese | 408 | RFS | 1.49 | 2.74 | 0.96 | [[15](#_ENREF_15)] |
| miR-605(rs2043556) | CRC | AG vs. GG | Jinliang Xing | 2012 | Chinese | 408 | OS | 1.01 | 1.61 | 0.96 | [[15](#_ENREF_15)] |
| miR-605(rs2043556) | CRC | AA vs. GG | Jinliang Xing | 2012 | Chinese | 408 | OS | 1.47 | 3.62 | 0.96 | [[15](#_ENREF_15)] |
| miR-605(rs2043556) | CRC | AG+AA vs. GG | Jinliang Xing | 2012 | Chinese | 408 | OS | 1.06 | 1.66 | 0.96 | [[15](#_ENREF_15)] |
| miR-605(rs2043556) | CRC | AA vs. AG+GG | Jinliang Xing | 2012 | Chinese | 408 | OS | 1.46 | 3.50 | 0.96 | [[15](#_ENREF_15)] |
| miR-605(rs2043556) | CRC | AG vs. GG | Jinliang Xing | 2012 | Chinese | 408 | RFS | 0.86 | 1.28 | 0.96 | [[15](#_ENREF_15)] |
| miR-605(rs2043556) | CRC | AA vs. GG | Jinliang Xing | 2012 | Chinese | 408 | RFS | 0.93 | 2.11 | 0.96 | [[15](#_ENREF_15)] |
| miR-605(rs2043556) | CRC | AG+AA vs. GG | Jinliang Xing | 2012 | Chinese | 408 | RFS | 0.87 | 1.27 | 0.96 | [[15](#_ENREF_15)] |
| miR-605(rs2043556) | CRC | AA vs. AG+GG | Jinliang Xing | 2012 | Chinese | 408 | RFS | 1.00 | 2.23 | 0.96 | [[15](#_ENREF_15)] |
| MiR-608 (rs4919510) | CRC | CG vs. GG | Jinliang Xing | 2012 | Chinese | 408 | OS | 0.67 | 1.10 | 0.96 | [[15](#_ENREF_15)] |
| MiR-608 (rs4919510) | CRC | CC vs. GG | Jinliang Xing | 2012 | Chinese | 408 | OS | 0.54 | 1.16 | 0.96 | [[15](#_ENREF_15)] |
| MiR-608 (rs4919510) | CRC | CG+CC vs. GG | Jinliang Xing | 2012 | Chinese | 408 | OS | 0.64 | 1.03 | 0.96 | [[15](#_ENREF_15)] |
| MiR-608 (rs4919510) | CRC | CC vs. CG+GG | Jinliang Xing | 2012 | Chinese | 408 | OS | 0.69 | 1.03 | 0.96 | [[15](#_ENREF_15)] |
| MiR-608 (rs4919510) | CRC | CG vs. GG | Jinliang Xing | 2012 | Chinese | 408 | RFS | 0.62 | 0.95 | 0.96 | [[15](#_ENREF_15)] |
| MiR-608 (rs4919510) | CRC | CC vs. GG | Jinliang Xing | 2012 | Chinese | 408 | RFS | 0.58 | 1.08 | 0.96 | [[15](#_ENREF_15)] |
| MiR-608 (rs4919510) | CRC | CG+CC vs. GG | Jinliang Xing | 2012 | Chinese | 408 | RFS | 0.61 | 0.92 | 0.96 | [[15](#_ENREF_15)] |
| MiR-608 (rs4919510) | CRC | CC vs. CG+GG | Jinliang Xing | 2012 | Chinese | 408 | RFS | 0.78 | 1.38 | 0.96 | [[15](#_ENREF_15)] |
| mir-608(rs4919510) | ESCC | GC vs. GG | Pei-Wen Yang | 2014 | Chinese | 504 | OS | 0.71 | 0.91 | 0.96 | [[34](#_ENREF_34)] |
| mir-608(rs4919510) | ESCC | CC vs. GG | Pei-Wen Yang | 2014 | Chinese | 504 | OS | 1.04 | 1.42 | 0.96 | [[34](#_ENREF_34)] |
| mir-608(rs4919510) | ESCC | CC+GG vs. GC | Pei-Wen Yang | 2014 | Chinese | 504 | OS | 1.42 | 1.76 | 0.96 | [[34](#_ENREF_34)] |
| mir-608(rs4919510) | ESCC | GC vs. GG | Pei-Wen Yang | 2014 | Chinese | 504 | RFS | 0.76 | 0.95 | 0.96 | [[34](#_ENREF_34)] |
| mir-608(rs4919510) | ESCC | CC vs. GG | Pei-Wen Yang | 2014 | Chinese | 504 | RFS | 0.99 | 1.32 | 0.96 | [[34](#_ENREF_34)] |
| mir-608(rs4919510) | ESCC | CC+GG vs. GC | Pei-Wen Yang | 2014 | Chinese | 504 | RFS | 1.31 | 1.60 | 0.96 | [[34](#_ENREF_34)] |
| miR-608(rs4919510) | NSCLC | CG vs. GG | Xia Lingzi | 2016 | Chinese | 584 | OS | 0.98 | 1.25 | 0.96 | [[19](#_ENREF_19)] |
| miR-608(rs4919510) | NSCLC | CC vs. GG | Xia Lingzi | 2016 | Chinese | 584 | OS | 1.02 | 1.38 | 0.96 | [[19](#_ENREF_19)] |
| miR-608(rs4919510) | HCC | CG vs. GG | Xiao-Pin Ma | 2016 | Chinese | 362 | OS | 0.81 | 1.10 | 0.96 | [[50](#_ENREF_50)] |
| miR-608(rs4919510) | HCC | CC vs. GG | Xiao-Pin Ma | 2016 | Chinese | 362 | OS | 0.62 | 0.93 | 0.96 | [[50](#_ENREF_50)] |
| miR-608(rs4919510) | HCC | CC vs. CG+GG | Xiao-Pin Ma | 2016 | Chinese | 362 | OS | 0.71 | 1.02 | 0.96 | [[50](#_ENREF_50)] |
| miR-608(rs4919510) | HCC | CG+CCvs. GG | Xiao-Pin Ma | 2016 | Chinese | 362 | OS | 0.76 | 1.01 | 0.96 | [[50](#_ENREF_50)] |
| mir-608(rs4919510) | CRC | CG vs. CC | Brı ´d M. Ryan | 2012 | American | 245 | OS | 1.06 | 1.60 | 0.96 | [[51](#_ENREF_51)] |
| mir-608(rs4919510) | CRC | GG vs. CC | Brı ´d M. Ryan | 2012 | American | 245 | OS | 1.23 | 2.60 | 0.96 | [[51](#_ENREF_51)] |
| miR-608(s4919510) | Breast cancer | CG vs. CC | Lianghe Jiao | 2014 | Chinese | 196 | OS | 1.32 | 2.41 | 0.96 | [[10](#_ENREF_10)] |
| miR-608(s4919510) | Breast cancer | GG vs. CC | Lianghe Jiao | 2014 | Chinese | 196 | OS | 1.42 | 3.11 | 0.96 | [[10](#_ENREF_10)] |
| miR-608(s4919510) | Breast cancer | CC vs. CG+GG | Lianghe Jiao | 2014 | Chinese | 196 | OS | 0.75 | 1.32 | 0.96 | [[10](#_ENREF_10)] |
| miR-608(s4919510) | Breast cancer | CG+CC vs.GG | Lianghe Jiao | 2014 | Chinese | 196 | OS | 0.83 | 1.64 | 0.96 | [[10](#_ENREF_10)] |
| mir-612(rs550894) | NSCLC | AC vs. CC | Yang Zhao | 2014 | American | 526 | OS | 0.74 | 0.97 | 0.96 | [[30](#_ENREF_30)] |
| mir-612(rs550894) | NSCLC | AA vs. CC | Yang Zhao | 2014 | American | 526 | OS | 0.30 | 0.95 | 0.96 | [[30](#_ENREF_30)] |
| miR-938(rs2505901) | GC | CT+TT vs. TT | Yanhua Wu | 2017 | Chinese | 735 | OS | 0.71 | 1.05 | 0.96 | [[39](#_ENREF_39)] |
| Note: HR: hazard ratio; HCC: hepatocellular carcinoma; NSCLC: non-small cell lung cancer; GC: gastric cancer; GAC: gastric adenocarcinoma; NCGC: non-cardia gastric cancer; SCCOP: squamous cell carcinoma of the nonoropharynx; CRC: colorectal cancer; ESCC: esophageal squamous cell carcinoma; HNSCC: head and neck squamous cell carcinoma; OSCC: oral squamous cell carcinoma; PTC: papillary Thyroid carcinoma. | | | | | | | | | | | |

| **Supplementary Table 2. Detailed information for NEWCASTLE - OTTAWA QUALITY ASSESSMENT SCALE** | | | | | | | | | | | |
| --- | --- | --- | --- | --- | --- | --- | --- | --- | --- | --- | --- |
| **Author** | **Year** | **Record number** | **Selection** | | | | **Comparability** | **Exposure/Outcome** | | | **Number^*^** |
|  | | | 1 | 2 | 3 | 4 |  | 1 | 2 | 3 |  |
| Brı ´d M. Ryan | 2012 | 313 | ***** | ***** | ***** | ***** | *** *** | ***** | ***** |  | **8** |
| Brock C. Christensen | 2010 | 359 | ***** | ***** | ***** | ***** | *** *** | ***** | ***** |  | **8** |
| Chang Zheng | 2017 | 62 | ***** | ***** |  | ***** | *** *** | ***** | ***** |  | **7** |
| Chengyuan Wang | 2016 | 116 | ***** | ***** |  | ***** | *** *** | ***** | ***** |  | **7** |
| Chung-Ji Liu | 2013 | 290 | ***** | ***** |  | ***** | *** *** | ***** | ***** |  | **7** |
| Dae Ho Ahn | 2013 | 295 | ***** | ***** |  | ***** | ****** | ***** | ***** |  | **7** |
| Fuzhen Qi | 2014 | 22 | ***** | ***** |  | ***** | ****** | ***** | ***** |  | **7** |
| Guopeng Yu | 2016 | 12 | ***** | ***** |  | ***** | ***** | ***** | ***** |  | **6** |
| Jeannette T. Bensen | 2013 | 439 | ***** | ***** | ***** | ***** | ***** | ***** | ***** |  | **7** |
| Jiali Xu | 2013 | 240 | ***** | ***** |  | ***** |  | ***** | ***** |  | **5** |
| Jiaze An | 2014 | 19 | ***** | ***** |  | ***** | *** *** | ***** | ***** |  | **7** |
| Jing Jiang | 2016 | 107 | ***** | ***** | ***** | ***** | ***** | ***** | ***** |  | **7** |
| Jinliang Xing | 2012 | 330 | ***** | ***** |  | ***** | ***** | ***** | ***** |  | **6** |
| Ji-Yong Ma | 2015 | 167 | ***** | ***** | ***** | ***** | ****** | ***** | ***** |  | **8** |
| Juan Li | 2016 | 34 | ***** | ***** | ***** | ***** | ****** | ***** | ***** |  | **8** |
| Kaipeng Xie | 2013 | 23 | ***** | ***** |  | ***** | ***** | ***** | ***** |  | **6** |
| Kaipeng Xie | 2015 | 138 | ***** | ***** | ***** | ***** | ***** | ***** | ***** |  | **7** |
| Kyong-Ah Yoon | 2012 | 303 | ***** | ***** |  | ***** | ***** | ***** | ***** |  | **6** |
| Kyung Min Shin | 2016 | 134 | ***** | ***** |  | ***** | ***** | ***** | ***** |  | **6** |
| Lianghe Jiao | 2014 | 175 | ***** | ***** | ***** | ***** | ***** | ***** | ***** |  | **7** |
| Lin Jiang | 2014 | 187 | ***** | ***** |  | ***** | *** *** | ***** | ***** |  | **7** |
| Meenakshi Umar | 2013 | 309 | ***** | ***** |  | ***** | *** *** | ***** | ***** |  | **7** |
| Meilin Wang | 2012 | 302 | ***** | ***** |  | ***** | ***** | ***** | ***** |  | **6** |
| Mi Jeong Hong | 2013 | 272 | ***** | ***** |  | ***** | ****** | ***** | ***** |  | **7** |
| MOON JU JANG | 2011 | 447 | ***** | ***** |  | ***** | ***** | ***** | ***** |  | **6** |
| Mulong Du | 2014 | 220 | ***** | ***** |  | ***** | *** *** | ***** | ***** |  | **7** |
| Myung Su Son | 2013 | 25 | ***** | ***** | ***** | ***** | ***** | ***** | ***** |  | **6** |
| Ning Zhan | 2013 | 250 | ***** | ***** | ***** | ***** | *** *** | ***** | ***** |  | **8** |
| Olusola O. Faluyi | 2017 | 79 | ***** | ***** |  | ***** | *** *** | ***** | ***** |  | **7** |
| Pei-Wen Yang | 2014 | 212 | ***** | ***** |  | ***** |  | ***** | ***** |  | **5** |
| Qian Xu | 2014 | 214 | ***** | ***** | ***** | ***** | ***** | ***** | ***** |  | **7** |
| Shizhi Wang | 2013 | 273 | ***** | ***** | ***** | ***** | ***** | ***** | ***** |  | **7** |
| Shuangshuang Wu | 2015 | 132 | ***** | ***** |  | ***** | ***** | ***** | ***** |  | **6** |
| SOO JUNG LEE | 2014 | 207 | ***** | ***** |  | ***** | ***** | ***** | ***** |  | **6** |
| Won Hee Kim | 2012 | 26 | ***** | ***** | ***** | ***** | ***** | ***** | ***** |  | **7** |
| Xia Lingzi | 2016 | 89 | ***** | ***** |  | ***** | ***** | ***** | ***** |  | **6** |
| Xiao-Pin Ma | 2016 | 11 | ***** | ***** |  | ***** | ***** | ***** | ***** |  | **6** |
| Xiaoxiang Guan | 2013 | 283 | ***** | ***** |  | ***** |  | ***** | ***** |  | **5** |
| Xi-Dai Long | 2016 | 14 | ***** | ***** |  | ***** | ***** | ***** | ***** |  | **6** |
| Xingming Chen | 2016 | 112 | ***** | ***** |  | ***** |  | ***** | ***** |  | **5** |
| Yang Zhao | 2014 | 200 | ***** | ***** |  | ***** |  | ***** | ***** |  | **5** |
| Yanhua Wu | 2017 | 74 |  | ***** | ***** | ***** |  | ***** | ***** |  | **5** |
| YEE SOO CHA | 2013 | 254 | ***** | ***** | ***** | ***** | *** *** | ***** | ***** |  | **8** |
| Yong-ping Mu | 2012 | 327 | ***** | ***** | ***** | ***** | *** *** | ***** | ***** |  | **8** |
| Z.Y. Sui | 2016 | 10 | ***** | ***** | ***** | ***** | *** *** | ***** | ***** |  | **8** |
| Zhibin Hu | 2008 | 376 | ***** | ***** |  | ***** | ***** | ***** | ***** |  | **6** |
| Zhibin Hu | 2011 | 469 | ***** | ***** |  | ***** | ***** | ***** | ***** |  | **6** |
| Ying Li | 2016 | 483 | ***** | ***** | ***** | ***** |  | ***** | ***** |  | **6** |
| Shizhi Wang | 2014 | 168 | ***** | ***** | ***** | ***** | ****** | ***** | ***** |  | **8** |
| JAEJOON LIM | 2018 | 1048 | ***** | ***** |  | ***** | ***** | ***** | ***** |  | **6** |
| Na Cao | 2018 | 776 | ***** | ***** |  | ***** | ****** | ***** | ***** |  | **7** |
| Marta Kotlarek | 2018 | 647 | ***** | ***** |  | ***** |  | ***** | ***** |  | **5** |
| Number* : means the number of " *" in each line.A article can be involved in a meta-analysis when the number is more than 5. | | | | | | | | | | | |

| **Supplementary Table 3. The results of Begg's and Egger's test for the publication bias** | | | | | | |
| --- | --- | --- | --- | --- | --- | --- |
|  |  | Begg's test | |  | Egger's test | |
| ncRNAs | Comparision type | Z value | P value |  | t value | P value |
| OS |  |  |  |  |  |  |
| let-7i(rs10877887) | CT+CC vs. TT | 0.00 | 1.000 |  | NA | NA |
| Let-7a-1(rs10739971) | GA vs. GG | 0.00 | 1.000 |  | NA | NA |
|  | AA vs. GG | 0.00 | 1.000 |  | NA | NA |
|  | GA+AA vs. GG | 0.00 | 1.000 |  | NA | NA |
| let-7a-2(rs629367) | AC vs. AA | 1.02 | 0.308 |  | 2.95 | 0.100 |
|  | CC vs. AA | 0.00 | 1.000 |  | 0.75 | 0.589 |
|  | AC+CC vs. AA | 0.00 | 1.000 |  | 0.47 | 0.719 |
| miR-218(rs11134527) | AG vs. AA | 0.00 | 1.000 |  | NA | NA |
|  | GG vs. AA | 0.00 | 1.000 |  | NA | NA |
|  | AG+GG vs. AA | 0.00 | 1.000 |  | NA | NA |
|  | GG vs. AG+AA | 0.00 | 1.000 |  | NA | NA |
| mir-26a-1(rs7372290) | CT vs. CC | 0.00 | 1.000 |  | NA | NA |
|  | TT vs. CC | 0.00 | 1.000 |  | NA | NA |
| miR-27a(rs895819) | CT vs. TT | 0.00 | 1.000 |  | -0.76 | 0.585 |
|  | CC vs. TT | 0.00 | 1.000 |  | -0.38 | 0.769 |
|  | CT+CC vs. TT | 1.02 | 0.308 |  | -0.7 | 0.557 |
|  | CC vs. CT+TT | 0.00 | 1.000 |  | NA | NA |
| miR-34b/c(rs4938723) | TC+CC vs. TT | 0.00 | 1.000 |  | NA | NA |
|  | CC vs. TC+TT | 0.00 | 1.000 |  | NA | NA |
| miR-423(rs6505162) | AC vs. CC | 0.24 | 0.806 |  | 0.41 | 0.708 |
|  | AA vs. CC | 0.73 | 0.462 |  | 1.07 | 0.362 |
|  | AC+AA vs. CC | 0.00 | 1.000 |  | NA | NA |
|  | AA vs. AC+CC | 0.00 | 1.000 |  | NA | NA |
| miR-492(rs2289030) | GC vs. CC | 0.00 | 1.000 |  | NA | NA |
|  | GG vs. CC | 0.00 | 1.000 |  | NA | NA |
|  | GG vs. CG+CC | 0.00 | 1.000 |  | NA | NA |
|  | GC+GG vs. CC | 0.00 | 1.000 |  | NA | NA |
| miR-499(rs3746444) | CT vs. TT | 0.38 | 0.707 |  | -0.42 | 0.693 |
|  | CC vs. TT | 0.24 | 0.806 |  | 0.58 | 0.603 |
|  | CC vs. CT+TT | 0.00 | 1.000 |  | NA | NA |
|  | CT+CC vs. TT | 0.00 | 1.000 |  | NA | NA |
|  | TT vs. CT+CC | 0.00 | 1.000 |  | NA | NA |
| miR-608(rs4919510) | CG vs. GG | -0.34 | 1.000 |  | -0.56 | 0.634 |
|  | CC vs. GG | 0.34 | 0.734 |  | -1.74 | 0.225 |
|  | CG vs. CC | 0.00 | 1.000 |  | NA | NA |
|  | GG vs. CC | 0.00 | 1.000 |  | NA | NA |
|  | CC vs. CG+GG | 0.00 | 1.000 |  | 0.27 | 0.830 |
|  | CG+CC vs.GG | 0.00 | 1.000 |  | -0.08 | 0.949 |
| miR-30c(rs928508) | AG+GG vs. AA | 0.00 | 1.000 |  | NA | NA |
| miR-378(rs1076064) | AG vs. AA | 1.04 | 0.296 |  | 3.35 | 0.185 |
|  | GG vs. AA | 0.00 | 1.000 |  | 0.72 | 0.602 |
| miR-146(rs2910164) | CG vs. CC | 0.75 | 0.452 |  | -0.75 | 0.495 |
|  | GG vs. CC | 0.37 | 0.711 |  | -0.18 | 0.864 |
|  | GC vs. GG | 0.00 | 1.000 |  | NA | NA |
|  | GC+GG vs. CC | 0.24 | 0.806 |  | 1.02 | 0.381 |
|  | GG vs. GC+CC | 0.30 | 0.764 |  | -1.04 | 0.346 |
| miR-149(rs2292832) | CT vs. TT | 0.24 | 0.806 |  | 0.16 | 0.881 |
|  | CC vs. TT | 0.38 | 0.707 |  | 0.19 | 0.855 |
|  | CT+CC vs. TT | 0.00 | 1.000 |  | 0.36 | 0.737 |
|  | CC vs. CT+TT | -0.24 | 1.000 |  | -0.2 | 0.857 |
| miR-196a2(rs11614913) | CT vs. TT | 0.30 | 0.764 |  | 0.63 | 0.557 |
|  | CC vs. TT | 0.00 | 1.000 |  | -0.13 | 0.905 |
|  | CT+CC vs. TT | 0.75 | 0.452 |  | -1.23 | 0.287 |
|  | CC vs. CT+TT | 0.87 | 0.386 |  | -0.9 | 0.402 |
|  | TC vs. CC | 0.00 | 1.000 |  | 0.31 | 0.808 |
|  | TT vs. CC | 1.04 | 0.296 |  | 3.81 | 0.164 |
|  | TC+TT vs. CC | 0.00 | 1.000 |  | NA | NA |
|  | TT vs. TC+CC | 0.00 | 1.000 |  | NA | NA |
| RFS |  |  |  |  |  |  |
| miR-27a(rs895819) | CT vs. TT | 0.00 | 1.000 |  | -0.75 | 0.592 |
|  | CC vs. TT | 0.00 | 1.000 |  | -4.05 | 0.154 |
|  | CT+CC vs.TT | 0.34 | 0.734 |  | -1.11 | 0.383 |
|  | CC vs. CT+TT | 0.00 | 1.000 |  | NA | NA |
| miR-423(rs6505162) | AC vs. CC | 0.00 | 1.000 |  | -0.77 | 0.582 |
|  | AA vs. CC | 0.00 | 1.000 |  | -0.54 | 0.682 |
|  | AC+AA vs. CC | 0.00 | 1.000 |  | NA | NA |
| miR-492(rs2289030) | GG vs. CC | 0.00 | 1.000 |  | NA | NA |
|  | GG vs. GC+CC | 0.00 | 1.000 |  | NA | NA |
| miR-608(rs4919510) | CG vs. GG | 0.00 | 1.000 |  | NA | NA |
|  | CC vs. GG | 0.00 | 1.000 |  | NA | NA |
| miR-146(rs2910164) | GC vs. CC | 0.00 | 1.000 |  | -0.54 | 0.683 |
|  | GG vs. CC | -0.34 | 1.000 |  | 0.00 | 1.000 |
|  | GG vs. GC+CC | 1.02 | 0.308 |  | -2.80 | 0.108 |
|  | GC+GG vs. CC | 0.00 | 1.000 |  | 0.46 | 0.723 |
|  | CC vs. GC+GG | 0.00 | 1.000 |  | NA | NA |
| miR-196a2(rs11614913) | CT vs. TT | 0.00 | 1.000 |  | NA | NA |
|  | CC vs. TT | 0.00 | 1.000 |  | NA | NA |
|  | CT+CC vs. TT | 0.00 | 1.000 |  | NA | NA |
| DFS |  |  |  |  |  |  |
| miR-499(rs3746444) | TT vs. CT+CC | 0.00 | 1.000 |  | 0.15 | 0.908 |
| miR-146(rs2910164) | GG vs. GC+CC | 0.00 | 1.000 |  | NA | NA |
| miR-149(rs2292832) | CC vs. CT+TT | 0.00 | 1.000 |  | 1.74 | 0.331 |
| miR-196a2(rs11614913) | CC vs. CT+TT | 0.00 | 1.000 |  | 0.74 | 0.596 |
|  | CT+TT vs. CC | 0.00 | 1.000 |  | NA | NA |
| Note: OS: overall survival; RFS: relapse-free survival; DFS: disease-free survival. | | | | | | |

| **Supplementary Table 4. Stratified data of the included articles in this meta-analysis** | | | | | | | |
| --- | --- | --- | --- | --- | --- | --- | --- |
| **Variables** | **miRNAs** | **cancer** | **model** | **outcome** | **HR** | **95% UPPER** | **95% LOWER** |
| Gender |  |  |  |  |  |  |  |
| Male | let-7i(rs10877887) | HCC | CT+CC vs. TT | OS | 1.14 | 1.40 | 0.94 |
|  | let-7a-2(rs1143770) | NSCLC | CT+TT vs. CC | OS | 0.65 | 0.90 | 0.47 |
|  | let-7a-1(rs10739971) | GC | GA vs. GG | OS | 0.92 | 1.64 | 0.52 |
|  | let-7a-1(rs10739971) | GC | AA vs. GG | OS | 0.86 | 1.79 | 0.41 |
|  | let-7a-1(rs10739971) | GC | GA+AA vs. GG | OS | 0.91 | 1.57 | 0.52 |
|  | let-7a-1(rs10739971) | GC | AA vs. GA+GG | OS | 0.91 | 1.71 | 0.49 |
|  | miR-106b-25(rs999885) | HCC | AG+GG vs. AA | OS | 0.72 | 0.93 | 0.55 |
|  | miR-149(rs2292832) | NSCLC | TC+CC vs. TT | OS | 0.76 | 1.10 | 0.53 |
|  | miR-196a(rs11614913) | NSCLC | CT+TT vs. CC | OS | 0.78 | 1.16 | 0.53 |
|  | miR-196a(rs11614913) | GC | CC vs. CT+TT | OS | 0.56 | 0.86 | 0.36 |
|  | miR-27a(rs895819) | NSCLC | CT+CC vs. TT | OS | 2.05 | 2.87 | 1.46 |
|  | miR-30c-1(rs928508) | NSCLC | AG+GG vs. AA | OS | 0.77 | 0.96 | 0.62 |
|  | miR-492(rs2289030) | HCC | CG vs. CC | OS | 0.67 | 0.92 | 0.49 |
|  | miR-492(rs2289030) | HCC | GG vs. CC | OS | 0.72 | 1.09 | 0.48 |
|  | miR-492(rs2289030) | HCC | CG+GG vs. CC | OS | 0.65 | 0.88 | 0.47 |
|  | miR-608(rs4919510) | HCC | CG vs. GG | OS | 0.77 | 1.08 | 0.56 |
|  | miR-608(rs4919510) | HCC | CC vs. GG | OS | 0.63 | 0.97 | 0.41 |
|  | miR-608(rs4919510) | HCC | CC vs. CG+GG | OS | 0.74 | 1.09 | 0.51 |
|  | let-7a-2(rs1143770) | NSCLC | CT+TT vs. CC | DFS | 0.73 | 0.96 | 0.55 |
|  | miR-149(rs2292832) | NSCLC | TC+CC vs. TT | DFS | 0.71 | 0.99 | 0.50 |
|  | miR-196a(rs11614913) | NSCLC | CT+TT vs. CC | DFS | 0.72 | 1.04 | 0.50 |
|  | miR-146a(rs2910164) | Bladder cancer | GC+CC vs. GG | RFS | 0.62 | 1.03 | 0.37 |
|  | miR-423(rs6505162) | CRC | CA+AA vs. CC | RFS | 2.17 | 3.74 | 1.26 |
|  | miR-608(rs4919510) | CRC | CG+CC vs. GG | RFS | 0.47 | 0.81 | 0.27 |
|  |  |  |  |  |  |  |  |
| Female | let-7i(rs10877887) | HCC | CT+CC vs. TT | OS | 1.76 | 3.22 | 0.96 |
|  | let-7a-2(rs1143770) | NSCLC | CT+TT vs. CC | OS | 0.90 | 2.02 | 0.40 |
|  | let-7a-1(rs10739971) | GC | GA vs. GG | OS | 0.47 | 1.02 | 0.22 |
|  | let-7a-1(rs10739971) | GC | AA vs. GG | OS | 0.24 | 0.86 | 0.07 |
|  | let-7a-1(rs10739971) | GC | GA+AA vs. GG | OS | 0.40 | 0.83 | 0.19 |
|  | let-7a-1(rs10739971) | GC | AA vs. GA+GG | OS | 0.39 | 1.29 | 0.12 |
|  | miR-106b-25(rs999885) | HCC | AG+GG vs. AA | OS | 0.99 | 2.13 | 0.46 |
|  | miR-149(rs2292832) | NSCLC | TC+CC vs. TT | OS | 0.32 | 0.82 | 0.13 |
|  | miR-196a(rs11614913) | NSCLC | CT+TT vs. CC | OS | 0.34 | 0.85 | 0.13 |
|  | miR-196a(rs11614913) | GC | CC vs. CT+TT | OS | 0.62 | 1.25 | 31.00 |
|  | miR-27a(rs895819) | NSCLC | CT+CC vs. TT | OS | 1.19 | 2.09 | 0.67 |
|  | miR-30c-1(rs928508) | NSCLC | AG+GG vs. AA | OS | 0.62 | 0.91 | 0.42 |
|  | miR-492(rs2289030) | HCC | CG vs. CC | OS | 1.13 | 2.27 | 0.57 |
|  | miR-492(rs2289030) | HCC | GG vs. CC | OS | 1.97 | 4.19 | 0.93 |
|  | miR-492(rs2289030) | HCC | CG+GG vs. CC | OS | 1.31 | 2.55 | 0.67 |
|  | miR-608(rs4919510) | HCC | CG vs. GG | OS | 0.88 | 1.83 | 0.43 |
|  | miR-608(rs4919510) | HCC | CC vs. GG | OS | 0.46 | 1.37 | 0.16 |
|  | miR-608(rs4919510) | HCC | CC vs. CG+GG | OS | 0.51 | 1.31 | 0.20 |
|  | let-7a-2(rs1143770) | NSCLC | CT+TT vs. CC | DFS | 0.76 | 1.32 | 0.43 |
|  | miR-149(rs2292832) | NSCLC | TC+CC vs. TT | DFS | 0.48 | 0.95 | 0.24 |
|  | miR-196a(rs11614913) | NSCLC | CT+TT vs. CC | DFS | 0.49 | 0.98 | 0.24 |
|  | miR-146a(rs2910164) | Bladder cancer | GC+CC vs. GG | RFS | 0.52 | 2.31 | 0.12 |
|  | miR-423(rs6505162) | CRC | CA+AA vs. CC | RFS | 1.12 | 2.13 | 0.60 |
|  | miR-608(rs4919510) | CRC | CG+CC vs. GG | RFS | 0.94 | 1.79 | 0.50 |
| Smoking |  |  |  |  |  |  |  |
| Yes | let-7i(rs10877887) | HCC | CT+CC vs. TT | OS | 1.25 | 1.77 | 0.88 |
|  | let-7a-2(rs1143770) | NSCLC | CT+TT vs. CC | OS | 0.60 | 0.83 | 0.43 |
|  | let-7a-1(rs10739971) | GC | GA vs. GG | OS | 1.43 | 4.36 | 0.47 |
|  | let-7a-1(rs10739971) | GC | AA vs. GG | OS | 1.92 | 6.81 | 0.54 |
|  | let-7a-1(rs10739971) | GC | GA+AA vs. GG | OS | 1.56 | 4.46 | 0.53 |
|  | let-7a-1(rs10739971) | GC | AA vs. GA+GG | OS | 1.49 | 3.75 | 0.59 |
|  | miR-106b-25(rs999885) | HCC | AG+GG vs. AA | OS | 0.68 | 0.92 | 0.50 |
|  | miR-146a(rs2910164) | SCCNOP | GG vs. CG+CC | OS | 0.90 | 1.10 | 0.70 |
|  | miR-149(rs2292832) | NSCLC | TC+CC vs. TT | OS | 0.72 | 1.04 | 0.50 |
|  | miR-149(rs2292832) | SCCNOP | CC vs. CT+TT | OS | 0.70 | 0.90 | 0.50 |
|  | miR-149(rs2292832) | NSCLC | CC vs. TT | OS | 0.62 | 0.98 | 0.39 |
|  | miR-196a(rs11614913) | NSCLC | CT+TT vs. CC | OS | 0.74 | 1.10 | 0.50 |
|  | miR-196a2(rs11614913) | SCCNOP | CC vs. CT+TT | OS | 1.00 | 1.30 | 0.80 |
|  | miR-27a(rs895819) | NSCLC | CT+CC vs. TT | OS | 2.08 | 3.08 | 1.40 |
|  | miR-30c-1(rs928508) | NSCLC | AG+GG vs. AA | OS | 0.76 | 0.96 | 0.60 |
|  | miR-492(rs2289030) | HCC | CG vs. CC | OS | 0.64 | 1.15 | 0.36 |
|  | miR-492(rs2289030) | HCC | GG vs. CC | OS | 1.34 | 2.19 | 0.82 |
|  | miR-492(rs2289030) | HCC | CG+GG vs. CC | OS | 0.8 | 1.21 | 0.54 |
|  | miR-499(rs3746444) | SCCNOP | TT vs. CT+CC | OS | 0.7 | 0.90 | 0.50 |
|  | let-7a-2(rs1143770) | NSCLC | CT+TT vs. CC | DFS | 0.67 | 0.89 | 0.51 |
|  | miR-146a(rs2910164) | SCCNOP | GG vs. CG+CC | DFS | 0.80 | 1.10 | 0.60 |
|  | miR-149(rs2292832) | NSCLC | TC+CC vs. TT | DFS | 0.65 | 0.92 | 0.24 |
|  | miR-149(rs2292832) | SCCNOP | CC vs. CT+TT | DFS | 0.70 | 0.90 | 0.60 |
|  | miR-196a(rs11614913) | NSCLC | CT+TT vs. CC | DFS | 0.66 | 0.94 | 0.46 |
|  | miR-196a2(rs11614913) | SCCNOP | CC vs. CT+TT | DFS | 0.90 | 1.20 | 0.70 |
|  | miR-499(rs3746444) | SCCNOP | TT vs. CT+CC | DFS | 0.50 | 0.90 | 0.40 |
|  | miR-146a(rs2910164) | Bladder cancer | GC+CC vs. GG | RFS | 0.82 | 1.66 | 0.40 |
|  | miR-423(rs6505162) | CRC | CA+AA vs. CC | RFS | 2.70 | 6.31 | 1.15 |
|  | miR-608(rs4919510) | CRC | CG+CC vs. GG | RFS | 0.34 | 0.88 | 0.13 |
|  |  |  |  |  |  |  |  |
| No | let-7i(rs10877887) | HCC | CT+CC vs. TT | OS | 1.11 | 1.40 | 0.89 |
|  | let-7a-2(rs1143770) | NSCLC | CT+TT vs. CC | OS | 1.29 | 2.83 | 0.58 |
|  | let-7a-1(rs10739971) | GC | GA vs. GG | OS | 0.76 | 1.54 | 0.38 |
|  | let-7a-1(rs10739971) | GC | AA vs. GG | OS | 0.36 | 1.25 | 0.10 |
|  | let-7a-1(rs10739971) | GC | GA+AA vs. GG | OS | 0.65 | 1.30 | 0.33 |
|  | let-7a-1(rs10739971) | GC | AA vs. GA+GG | OS | 0.41 | 1.33 | 0.13 |
|  | miR-106b-25(rs999885) | HCC | AG+GG vs. AA | OS | 0.92 | 1.43 | 0.60 |
|  | miR-146a(rs2910164) | SCCNOP | GG vs. CG+CC | OS | 1.30 | 2.20 | 0.70 |
|  | miR-149(rs2292832) | NSCLC | TC+CC vs. TT | OS | 0.46 | 1.06 | 0.20 |
|  | miR-149(rs2292832) | SCCNOP | CC vs. CT+TT | OS | 0.90 | 1.30 | 0.40 |
|  | miR-149(rs2292832) | NSCLC | CC vs. TT | OS | 0.38 | 0.69 | 0.21 |
|  | miR-149(rs2292832) | NSCLC | CT vs. TT | OS | 0.54 | 0.77 | 0.38 |
|  | miR-196a(rs11614913) | NSCLC | CT+TT vs. CC | OS | 0.44 | 1.08 | 0.18 |
|  | miR-196a2(rs11614913) | SCCNOP | CC vs. CT+TT | OS | 2.00 | 3.80 | 1.10 |
|  | miR-27a(rs895819) | NSCLC | CT+CC vs. TT | OS | 1.21 | 1.87 | 0.78 |
|  | miR-30c-1(rs928508) | NSCLC | AG+GG vs. AA | OS | 0.76 | 1.05 | 0.55 |
|  | miR-492(rs2289030) | HCC | CG vs. CC | OS | 0.74 | 1.04 | 0.52 |
|  | miR-492(rs2289030) | HCC | GG vs. CC | OS | 0.63 | 1.05 | 0.38 |
|  | miR-492(rs2289030) | HCC | CG+GG vs. CC | OS | 0.62 | 0.92 | 0.42 |
|  | miR-499(rs3746444) | SCCNOP | TT vs. CT+CC | OS | 0.9 | 1.40 | 0.30 |
|  | let-7a-2(rs1143770) | NSCLC | CT+TT vs. CC | DFS | 0.99 | 0.58 | 1.70 |
|  | miR-146a(rs2910164) | SCCNOP | GG vs. CG+CC | DFS | 1.40 | 2.50 | 0.80 |
|  | miR-149(rs2292832) | NSCLC | TC+CC vs. TT | DFS | 0.64 | 1.23 | 0.33 |
|  | miR-149(rs2292832) | SCCNOP | CC vs. CT+TT | DFS | 1.00 | 1.70 | 0.50 |
|  | miR-196a(rs11614913) | NSCLC | CT+TT vs. CC | DFS | 0.61 | 1.22 | 0.30 |
|  | miR-196a2(rs11614913) | SCCNOP | CC vs. CT+TT | DFS | 2.00 | 3.50 | 2.00 |
|  | miR-499(rs3746444) | SCCNOP | TT vs. CT+CC | DFS | 0.80 | 1.40 | 0.60 |
|  | miR-146a(rs2910164) | Bladder cancer | GC+CC vs. GG | RFS | 0.41 | 0.80 | 0.21 |
|  | miR-423(rs6505162) | CRC | CA+AA vs. CC | RFS | 1.44 | 2.27 | 0.92 |
|  | miR-608(rs4919510) | CRC | CG+CC vs. GG | RFS | 0.69 | 1.11 | 0.43 |
| Drinking |  |  |  |  |  |  |  |
| Yes | let-7i(rs10877887) | HCC | CT+CC vs. TT | OS | 1.29 | 1.79 | 0.92 |
|  | let-7a-1(rs10739971) | GC | GA vs. GG | OS | 0.80 | 2.58 | 0.25 |
|  | let-7a-1(rs10739971) | GC | AA vs. GG | OS | 0.90 | 3.22 | 0.25 |
|  | let-7a-1(rs10739971) | GC | GA+AA vs. GG | OS | 0.84 | 2.53 | 0.28 |
|  | let-7a-1(rs10739971) | GC | AA vs. GA+GG | OS | 1.08 | 2.82 | 0.41 |
|  | miR-106b-25(rs999885) | HCC | AG+GG vs. AA | OS | 0.70 | 0.94 | 0.52 |
|  | miR-492(rs2289030) | HCC | CG vs. CC | OS | 0.59 | 0.89 | 0.40 |
|  | miR-492(rs2289030) | HCC | GG vs. CC | OS | 1.01 | 1.49 | 0.68 |
|  | miR-492(rs2289030) | HCC | CG+GG vs. CC | OS | 0.65 | 0.94 | 0.44 |
|  | miR-423(rs6505162) | CRC | CA+AA vs. CC | RFS | 7.88 | 50.07 | 1.24 |
|  | miR-608(rs4919510) | CRC | CG+CC vs. GG | RFS | 0.46 | 1.90 | 0.11 |
|  |  |  |  |  |  |  |  |
| No | let-7i(rs10877887) | HCC | CT+CC vs. TT | OS | 1.17 | 0.93 | 1.48 |
|  | let-7a-1(rs10739971) | GC | GA vs. GG | OS | 0.98 | 1.93 | 0.50 |
|  | let-7a-1(rs10739971) | GC | AA vs. GG | OS | 0.55 | 1.92 | 0.16 |
|  | let-7a-1(rs10739971) | GC | GA+AA vs. GG | OS | 0.90 | 1.74 | 0.46 |
|  | let-7a-1(rs10739971) | GC | AA vs. GA+GG | OS | 0.56 | 1.81 | 0.17 |
|  | miR-106b-25(rs999885) | HCC | AG+GG vs. AA | OS | 0.88 | 1.39 | 0.56 |
|  | miR-492(rs2289030) | HCC | CG vs. CC | OS | 0.92 | 1.43 | 0.60 |
|  | miR-492(rs2289030) | HCC | GG vs. CC | OS | 0.63 | 1.28 | 0.31 |
|  | miR-492(rs2289030) | HCC | CG+GG vs. CC | OS | 0.86 | 1.31 | 0.57 |
|  | miR-423(rs6505162) | CRC | CA+AA vs. CC | RFS | 1.50 | 2.30 | 0.98 |
|  | miR-608(rs4919510) | CRC | CG+CC vs. GG | RFS | 0.64 | 1.00 | 0.41 |
| Family History | |  |  |  |  |  |  |
| Yes | let-7a-1(rs10739971) | GC | GA vs. GG | OS | 1.05 | 11.55 | 0.10 |
|  | let-7a-1(rs10739971) | GC | AA vs. GG | OS | 5.25 | 58.6 | 0.47 |
|  | let-7a-1(rs10739971) | GC | GA+AA vs. GG | OS | 1.69 | 15.08 | 0.19 |
|  | let-7a-1(rs10739971) | GC | AA vs. GA+GG | OS | 5.09 | 30.78 | 0.84 |
|  | miR-125a(rs12976445) | Breast cancer | TT+CT vs. CC | OS | 1.04 | 3.27 | 0.33 |
|  | miR-125a(rs12976445) | Breast cancer | TT vs. CT+CC | OS | 2.36 | 18.32 | 0.31 |
|  | miR-492(rs2289030) | HCC | CG vs. CC | OS | 0.48 | 0.85 | 0.27 |
|  | miR-492(rs2289030) | HCC | GG vs. CC | OS | 0.74 | 1.54 | 0.36 |
|  | miR-492(rs2289030) | HCC | CG+GG vs. CC | OS | 0.50 | 1.88 | 0.46 |
|  | miR-608(rs4919510) | HCC | CG vs. GG | OS | 1.05 | 1.87 | 0.59 |
|  | miR-608(rs4919510) | HCC | CC vs. GG | OS | 0.56 | 1.32 | 0.24 |
|  | miR-608(rs4919510) | HCC | CC vs. CG+GG | OS | 0.54 | 1.14 | 0.25 |
|  |  |  |  |  |  |  |  |
| No | let-7a-1(rs10739971) | GC | GA vs. GG | OS | 0.88 | 1.6 | 0.48 |
|  | let-7a-1(rs10739971) | GC | AA vs. GG | OS | 0.55 | 1.35 | 0.23 |
|  | let-7a-1(rs10739971) | GC | GA+AA vs. GG | OS | 0.79 | 1.41 | 0.44 |
|  | let-7a-1(rs10739971) | GC | AA vs. GA+GG | OS | 0.59 | 1.30 | 0.27 |
|  | miR-125a(rs12976445) | Breast cancer | TT+CT vs. CC | OS | 2.00 | 3.81 | 1.04 |
|  | miR-125a(rs12976445) | Breast cancer | TT vs. CT+CC | OS | 3.67 | 15.30 | 0.88 |
|  | miR-492(rs2289030) | HCC | CG vs. CC | OS | 0.79 | 1.12 | 0.55 |
|  | miR-492(rs2289030) | HCC | GG vs. CC | OS | 0.94 | 1.38 | 0.64 |
|  | miR-492(rs2289030) | HCC | CG+GG vs. CC | OS | 0.79 | 1.11 | 0.57 |
|  | miR-608(rs4919510) | HCC | CG vs. GG | OS | 0.73 | 1.05 | 0.51 |
|  | miR-608(rs4919510) | HCC | CC vs. GG | OS | 0.67 | 1.07 | 0.42 |
|  | miR-608(rs4919510) | HCC | CC vs. CG+GG | OS | 0.82 | 1.23 | 0.55 |
| Lymphatic metastasis | |  |  |  |  |  |  |
| Yes | let-7a-1(rs10739971) | GC | GA vs. GG | OS | 0.78 | 1.28 | 0.48 |
|  | let-7a-1(rs10739971) | GC | AA vs. GG | OS | 0.78 | 1.54 | 0.39 |
|  | let-7a-1(rs10739971) | GC | GA+AA vs. GG | OS | 0.78 | 1.25 | 0.49 |
|  | let-7a-1(rs10739971) | GC | AA vs. GA+GG | OS | 0.91 | 1.68 | 0.49 |
|  | miR-125a(rs12976445) | Breast cancer | TT+CT vs. CC | OS | 1.72 | 3.20 | 0.92 |
|  | miR-125a(rs12976445) | Breast cancer | TT vs. CT+CC | OS | 2.94 | 8.26 | 1.04 |
|  |  |  |  |  |  |  |  |
| No | let-7a-1(rs10739971) | GC | GA vs. GG | OS | 0.71 | 2.43 | 0.21 |
|  | let-7a-1(rs10739971) | GC | AA vs. GG | OS | 0.58 | 2.60 | 0.13 |
|  | let-7a-1(rs10739971) | GC | GA+AA vs. GG | OS | 0.67 | 2.13 | 0.21 |
|  | let-7a-1(rs10739971) | GC | AA vs. GA+GG | OS | 0.74 | 2.63 | 0.21 |
|  | miR-125a(rs12976445) | Breast cancer | TT+CT vs. CC | OS | 1.78 | 4.92 | 0.65 |
|  | miR-125a(rs12976445) | Breast cancer | TT vs. CT+CC | OS | 3.68 | 28.26 | 0.48 |
| Note: HR: hazard ratio; HCC: hepatocellular carcinoma; NSCLC: non-small cell lung cancer; GC: gastric cancer; SCCNOP: squamous cell carcinoma of the nonoropharynx; CRC: colorectal cancer. | | | | | | | |

**Reference**

**1. Sui ZY, Li J, Cheng GL, Wang SF: A single nucleotide polymorphism in the promoter region (rs10877887) of let-7 is associated with hepatocellular carcinoma in a Chinese population. *Genetics and molecular research : GMR* 2016, 15(2).**

**2. Xie K, Liu J, Zhu L, Liu Y, Pan Y, Wen J, Ma H, Zhai X, Hu Z: A potentially functional polymorphism in the promoter region of let-7 family is associated with survival of hepatocellular carcinoma. *Cancer epidemiology* 2013, 37(6):998-1002.**

**3. Shin KM, Jung DK, Hong MJ, Kang HJ, Lee WK, Yoo SS, Lee SY, Cha SI, Lee J, Kim CH *et al*: The pri-let-7a-2 rs1143770C>T is associated with prognosis of surgically resected non-small cell lung cancer. *Gene* 2016, 577(2):148-152.**

**4. Li Y, Xu Q, Liu J, He C, Yuan Q, Xing C, Yuan Y: Pri-let-7a-1 rs10739971 polymorphism is associated with gastric cancer prognosis and might affect mature let-7a expression. *OncoTargets and therapy* 2016, 9:3951-3962.**

**5. Hu Z, Shu Y, Chen Y, Chen J, Dong J, Liu Y, Pan S, Xu L, Xu J, Wang Y *et al*: Genetic polymorphisms in the precursor MicroRNA flanking region and non-small cell lung cancer survival. *American journal of respiratory and critical care medicine* 2011, 183(5):641-648.**

**6. Xu Q, Dong Q, He C, Liu W, Sun L, Liu J, Xing C, Li X, Wang B, Yuan Y: A new polymorphism biomarker rs629367 associated with increased risk and poor survival of gastric cancer in chinese by up-regulated miRNA-let-7a expression. *PloS one* 2014, 9(4):e95249.**

**7. Qi F, Huang M, Pan Y, Liu Y, Liu J, Wen J, Xie K, Shen H, Ma H, Miao Y *et al*: A genetic variant in the promoter region of miR-106b-25 cluster predict clinical outcome of HBV-related hepatocellular carcinoma in Chinese. *PloS one* 2014, 9(1):e85394.**

**8. Wang S, Lv C, Jin H, Xu M, Kang M, Chu H, Tong N, Wu D, Zhu H, Gong W *et al*: A common genetic variation in the promoter of miR-107 is associated with gastric adenocarcinoma susceptibility and survival. *Mutation research* 2014, 769:35-41.**

**9. Wu S, Shen W, Pan Y, Zhu M, Xie K, Geng L, Wang Y, Liang Y, Xu J, Cao S *et al*: Genetic Variations in Key MicroRNAs are Associated With the Survival of Nonsmall Cell Lung Cancer. *Medicine* 2015, 94(47):e2084.**

**10. Jiao L, Zhang J, Dong Y, Duan B, Yu H, Sheng H, Huang J, Gao H: Association between miR-125a rs12976445 and survival in breast cancer patients. *American journal of translational research* 2014, 6(6):869-875.**

**11. Long XD, Huang XY, Yao JG, Liao P, Tang YJ, Ma Y, Xia Q: Polymorphisms in the precursor microRNAs and aflatoxin B1-related hepatocellular carcinoma. *Molecular carcinogenesis* 2016, 55(6):1060-1072.**

**12. Chen X, Sturgis EM, Wang C, Cao X, Li Y, Wei Q, Li G: Significance of microRNA-related variants in susceptibility to recurrence of oropharyngeal cancer patients after definitive radiotherapy. *Oncotarget* 2016, 7(23):35015-35025.**

**13. Yoon KA, Yoon H, Park S, Jang HJ, Zo JI, Lee HS, Lee JS: The prognostic impact of microRNA sequence polymorphisms on the recurrence of patients with completely resected non-small cell lung cancer. *The Journal of thoracic and cardiovascular surgery* 2012, 144(4):794-807.**

**14. Jang MJ, Kim JW, Min KT, Jeon YJ, Oh D, Kim NK: Prognostic significance of microRNA gene polymorphisms in patients with surgically resected colorectal cancer. *Experimental and therapeutic medicine* 2011, 2(6):1127-1132.**

**15. Xing J, Wan S, Zhou F, Qu F, Li B, Myers RE, Fu X, Palazzo JP, He X, Chen Z *et al*: Genetic polymorphisms in pre-microRNA genes as prognostic markers of colorectal cancer. *Cancer epidemiology, biomarkers & prevention : a publication of the American Association for Cancer Research, cosponsored by the American Society of Preventive Oncology* 2012, 21(1):217-227.**

**16. Hong MJ, Choi YY, Jang JA, Jung HJ, Lee SY, Lee WK, Yoo SS, Lee J, Cha SI, Kim CH *et al*: Association between genetic variants in pre-microRNAs and survival of early-stage NSCLC. *Journal of thoracic oncology : official publication of the International Association for the Study of Lung Cancer* 2013, 8(6):703-710.**

**17. Hu Z, Chen J, Tian T, Zhou X, Gu H, Xu L, Zeng Y, Miao R, Jin G, Ma H *et al*: Genetic variants of miRNA sequences and non-small cell lung cancer survival. *The Journal of clinical investigation* 2008, 118(7):2600-2608.**

**18. Wang C, Sturgis EM, Chen X, Zheng H, Wei Q, Li G: Pre-miRNA variants as predictors of clinical outcome in patients with squamous cell carcinomas of the nonoropharynx. *Oncotarget* 2016, 7(18):26444-26453.**

**19. Lingzi X, Zhihua Y, Xuelian L, Yangwu R, Haibo Z, Yuxia Z, Baosen Z: Genetic variants in microRNAs predict non-small cell lung cancer prognosis in Chinese female population in a prospective cohort study. *Oncotarget* 2016, 7(50):83101-83114.**

**20. Guan X, Sturgis EM, Song X, Liu Z, El-Naggar AK, Wei Q, Li G: Pre-microRNA variants predict HPV16-positive tumors and survival in patients with squamous cell carcinoma of the oropharynx. *Cancer letters* 2013, 330(2):233-240.**

**21. Chae YS, Kim JG, Lee SJ, Kang BW, Lee YJ, Park JY, Jeon HS, Park JS, Choi GS: A miR-146a polymorphism (rs2910164) predicts risk of and survival from colorectal cancer. *Anticancer research* 2013, 33(8):3233-3239.**

**22. Wang M, Chu H, Li P, Yuan L, Fu G, Ma L, Shi D, Zhong D, Tong N, Qin C *et al*: Genetic variants in miRNAs predict bladder cancer risk and recurrence. *Cancer research* 2012, 72(23):6173-6182.**

**23. Jiang J, Jia ZF, Cao DH, Wu YH, Sun ZW, Cao XY: Association of the miR-146a rs2910164 polymorphism with gastric cancer susceptibility and prognosis. *Future oncology (London, England)* 2016, 12(19):2215-2226.**

**24. Ahn DH, Rah H, Choi YK, Jeon YJ, Min KT, Kwack K, Hong SP, Hwang SG, Kim NK: Association of the miR-146aC>G, miR-149T>C, miR-196a2T>C, and miR-499A>G polymorphisms with gastric cancer risk and survival in the Korean population. *Molecular carcinogenesis* 2013, 52 Suppl 1:E39-51.**

**25. Kim WH, Min KT, Jeon YJ, Kwon CI, Ko KH, Park PW, Hong SP, Rim KS, Kwon SW, Hwang SG *et al*: Association study of microRNA polymorphisms with hepatocellular carcinoma in Korean population. *Gene* 2012, 504(1):92-97.**

**26. Umar M, Upadhyay R, Prakash G, Kumar S, Ghoshal UC, Mittal B: Evaluation of common genetic variants in pre-microRNA in susceptibility and prognosis of esophageal cancer. *Molecular carcinogenesis* 2013, 52 Suppl 1:E10-18.**

**27. Kotlarek M, Kubiak A, Czetwertynska M, Swierniak M, Gierlikowski W, Kolanowska M, Bakula-Zalewska E, Jhiang SM, Jazdzewski K, Wojcicka A: The rs2910164 Genetic Variant of miR-146a-3p Is Associated with Increased Overall Mortality in Patients with Follicular Variant Papillary Thyroid Carcinoma. *International journal of molecular sciences* 2018, 19(3).**

**28. Xie K, Ma H, Liang C, Wang C, Qin N, Shen W, Gu Y, Yan C, Zhang K, Dai N *et al*: A functional variant in miR-155 regulation region contributes to lung cancer risk and survival. *Oncotarget* 2015, 6(40):42781-42792.**

**29. Bensen JT, Tse CK, Nyante SJ, Barnholtz-Sloan JS, Cole SR, Millikan RC: Association of germline microRNA SNPs in pre-miRNA flanking region and breast cancer risk and survival: the Carolina Breast Cancer Study. *Cancer causes & control : CCC* 2013, 24(6):1099-1109.**

**30. Zhao Y, Wei Q, Hu L, Chen F, Hu Z, Heist RS, Su L, Amos CI, Shen H, Christiani DC: Polymorphisms in MicroRNAs are associated with survival in non-small cell lung cancer. *Cancer epidemiology, biomarkers & prevention : a publication of the American Association for Cancer Research, cosponsored by the American Society of Preventive Oncology* 2014, 23(11):2503-2511.**

**31. Lee SJ, Seo JW, Chae YS, Kim JG, Kang BW, Kim WW, Jung JH, Park HY, Jeong JY, Park JY: Genetic polymorphism of miR-196a as a prognostic biomarker for early breast cancer. *Anticancer research* 2014, 34(6):2943-2949.**

**32. Du M, Lu D, Wang Q, Chu H, Tong N, Pan X, Qin C, Yin C, Wang M, Zhang Z: Genetic variations in microRNAs and the risk and survival of renal cell cancer. *Carcinogenesis* 2014, 35(7):1629-1635.**

**33. Christensen BC, Avissar-Whiting M, Ouellet LG, Butler RA, Nelson HH, McClean MD, Marsit CJ, Kelsey KT: Mature microRNA sequence polymorphism in MIR196A2 is associated with risk and prognosis of head and neck cancer. *Clinical cancer research : an official journal of the American Association for Cancer Research* 2010, 16(14):3713-3720.**

**34. Yang PW, Huang YC, Hsieh CY, Hua KT, Huang YT, Chiang TH, Chen JS, Huang PM, Hsu HH, Kuo SW *et al*: Association of miRNA-related genetic polymorphisms and prognosis in patients with esophageal squamous cell carcinoma. *Annals of surgical oncology* 2014, 21 Suppl 4:S601-609.**

**35. Liu CJ, Tsai MM, Tu HF, Lui MT, Cheng HW, Lin SC: miR-196a overexpression and miR-196a2 gene polymorphism are prognostic predictors of oral carcinomas. *Annals of surgical oncology* 2013, 20 Suppl 3:S406-414.**

**36. Wang S, Tao G, Wu D, Zhu H, Gao Y, Tan Y, Wang M, Gong W, Zhou Y, Zhou J *et al*: A functional polymorphism in MIR196A2 is associated with risk and prognosis of gastric cancer. *Molecular carcinogenesis* 2013, 52 Suppl 1:E87-95.**

**37. Li J, Cheng G, Wang S: A Single-Nucleotide Polymorphism of miR-196a2T>C rs11614913 Is Associated with Hepatocellular Carcinoma in the Chinese Population. *Genetic testing and molecular biomarkers* 2016, 20(4):213-215.**

**38. Lim J, Kim JO, Park HS, Han IB, Kwack K, Kim NK, Cho K: Associations of miR146aC>G, miR149C>T, miR196a2C>T and miR499A>G polymorphisms with brain tumors. *Oncology reports* 2018, 40(3):1813-1823.**

**39. Wu Y, Jia Z, Cao D, Wang C, Wu X, You L, Wen S, Pan Y, Cao X, Jiang J: Predictive Value of MiR-219-1, MiR-938, MiR-34b/c, and MiR-218 Polymorphisms for Gastric Cancer Susceptibility and Prognosis. *Disease markers* 2017, 2017:4731891.**

**40. Jiang L, Wang C, Sun C, Xu Y, Ding Z, Zhang X, Huang J, Yu H: The impact of pri-miR-218 rs11134527 on the risk and prognosis of patients with esophageal squamous cell carcinoma. *International journal of clinical and experimental pathology* 2014, 7(9):6206-6212.**

**41. Zheng C, Li X, Xia L, Fang X, Quan X, Yin Z, Zhao Y, Zhou B: Polymorphisms of pri-miR-219-1 are associated with the susceptibility and prognosis of non-small cell lung cancer in a Northeast Chinese population. *Oncotarget* 2017, 8(34):56533-56541.**

**42. Zhang N, Huo Q, Wang X, Chen X, Long L, Jiang L, Ma T, Yang Q: A genetic variant in pre-miR-27a is associated with a reduced breast cancer risk in younger Chinese population. *Gene* 2013, 529(1):125-130.**

**43. Ma JY, Yan HJ, Yang ZH, Gu W: Rs895819 within miR-27a might be involved in development of non small cell lung cancer in the Chinese Han population. *Asian Pacific journal of cancer prevention : APJCP* 2015, 16(5):1939-1944.**

**44. Xu J, Yin Z, Shen H, Gao W, Qian Y, Pei D, Liu L, Shu Y: A genetic polymorphism in pre-miR-27a confers clinical outcome of non-small cell lung cancer in a Chinese population. *PloS one* 2013, 8(11):e79135.**

**45. Mu YP, Su XL: Polymorphism in pre-miR-30c contributes to gastric cancer risk in a Chinese population. *Medical oncology (Northwood, London, England)* 2012, 29(3):1723-1732.**

**46. Son MS, Jang MJ, Jeon YJ, Kim WH, Kwon CI, Ko KH, Park PW, Hong SP, Rim KS, Kwon SW *et al*: Promoter polymorphisms of pri-miR-34b/c are associated with hepatocellular carcinoma. *Gene* 2013, 524(2):156-160.**

**47. An J, Liu J, Liu L, Liu Y, Pan Y, Huang M, Qi F, Wen J, Xie K, Ma H *et al*: A genetic variant in primary miR-378 is associated with risk and prognosis of hepatocellular carcinoma in a Chinese population. *PloS one* 2014, 9(4):e93707.**

**48. Cao N, Li M, Han J, Wang Y, Wang X: rs61991156 in miR-379 is associated with low capability of glycolysis of gastric cancer by enhanced regulation of PKM2. *Cancer cell international* 2018, 18:92.**

**49. Yu G, Xiao Q, Ma XP, Chen X, Shi Z, Zhang LY, Chen H, Zhang P, Ding DL, Huang HX *et al*: miR-492G>C polymorphism (rs2289030) is associated with overall survival of hepatocellular carcinoma patients. *Tumour biology : the journal of the International Society for Oncodevelopmental Biology and Medicine* 2016, 37(7):8961-8972.**

**50. Ma XP, Yu G, Chen X, Xiao Q, Shi Z, Zhang LY, Chen H, Zhang P, Ding DL, Huang HX *et al*: MiR-608 rs4919510 is associated with prognosis of hepatocellular carcinoma. *Tumour biology : the journal of the International Society for Oncodevelopmental Biology and Medicine* 2016, 37(7):9931-9942.**

**51. Ryan BM, McClary AC, Valeri N, Robinson D, Paone A, Bowman ED, Robles AI, Croce C, Harris CC: rs4919510 in hsa-mir-608 is associated with outcome but not risk of colorectal cancer. *PloS one* 2012, 7(5):e36306.**
